# Nutritional Strategies for Optimizing Health, Sports Performance, and Recovery for Female Athletes and Other Physically Active Women: A Systematic Review

**DOI:** 10.1093/nutrit/nuae082

**Published:** 2024-07-12

**Authors:** Mar Larrosa, Angel Gil-Izquierdo, Liliana Guadalupe González-Rodríguez, María José Muñoz Alférez, Alejandro F San Juan, Ángela Sánchez-Gómez, Natalia Calvo-Ayuso, Juan José Ramos-Álvarez, Diego Fernández-Lázaro, Raúl Lopez-Grueso, Inmaculada López-León, Javier Moreno-Lara, Diego Domínguez-Balmaseda, Román Illescas-Quiroga, Eduardo Cuenca, Teba López, Juan José Montoya, Daiana Priscila Rodrigues-de-Souza, Elena Carrillo-Alvarez, Arturo Casado, Belén Rodriguez-Doñate, Mireia Porta-Oliva, Catalina Santiago, Támara Iturriaga, Beatriz De Lucas, Ángela García Solaesa, María del Pilar Montero-López, Elvira Benítez De Gracia, Pablo Veiga-Herreros, Alejandro Muñoz-López, Eva Orantes-Gonzalez, José Carlos Barbero-Alvarez, Ruth Cabeza-Ruiz, Ángel Carnero-Diaz, Isabel Sospedra, Luis Miguel Fernández-Galván, José Miguel Martínez-Sanz, Francisco Javier Martín-Almena, Margarita Pérez, Eduardo J Guerra-Hernández, Álvaro López-Samanes, Antonio Jesús Sánchez-Oliver, Raúl Domínguez

**Affiliations:** Departamento de Nutrición y Ciencia de los Alimentos, Facultad de Farmacia, Universidad Complutense de Madrid, 28040 Madrid, Spain; Research Group on Food and Nutrition (ALINUT), University of Alicante, 03690 Alicante, Spain; Quality, Safety, and Bioactivity of Plant Foods Group, Department of Food Science and Technology, CEBAS-CSIC, University of Murcia, 30100 Murcia, Spain; Departamento de Nutrición y Ciencia de los Alimentos, Facultad de Farmacia, Universidad Complutense de Madrid, 28040 Madrid, Spain; VALORNUT Research Group, Complutense University of Madrid, 28040 Madrid, Spain; Department of Physiology (Faculty of Pharmacy, Cartuja University Campus), Institute of Nutrition and Food Technology “José Mataix”, University of Granada, 18071 Granada, Spain; Department of Health and Human Performance, Faculty of Physical Activity and Sports Sciences (INEF), Universidad Politécnica de Madrid, 28040 Madrid, Spain; Departamento de Enfermería, Farmacología y Fisioterapia, 14004 Córdoba, Spain; Departamento de Enfermería y Fisioterapia, Campus de Ponferrada, Universidad de León, 24401 Ponferrada, Spain; School of Sport Medicine, Department of Radiology, Rehabilitation and Physiotherapy, Complutense University Madrid, 28040 Madrid, Spain; Department of Cellular Biology, Genetics, Histology and Pharmacology, Faculty of Health Sciences, University of Valladolid, 42004 Soria, Spain; Neurobiology Research Group, Faculty of Medicine, University of Valladolid, 47005 Valladolid, Spain; Facultad de Ciencias de la Salud, Universidad Isabel I, 09003 Burgos, Spain; Departamento de Motricidad Humana y Rendimiento Deportivo, University of Seville, 41013 Seville, Spain; Departamento de Motricidad Humana y Rendimiento Deportivo, University of Seville, 41013 Seville, Spain; Facultad de Ciencias de la Actividad Física, Deporte y Fisioterapia, Universidad Europea de Madrid, 28670 Villaviciosa de Odón, Spain; Departamento de Enfermería y Fisioterapia, University of Alcala, 28805 Alcalá de Henares, Spain; Academia de Guardias y Suboficiales de la Guardia Civil, 23440 Baeza, Spain; Academia de Guardias y Suboficiales de la Guardia Civil, 23440 Baeza, Spain; School of Sport Medicine, Department of Radiology, Rehabilitation and Physiotherapy, Complutense University Madrid, 28040 Madrid, Spain; Departamento de Enfermería, Farmacología y Fisioterapia, 14004 Córdoba, Spain; Maimonides Biomedical Research Institute of Córdoba (IMIBIC), 14004 Córdoba, Spain; Global Research on Wellbeing (GRoW) Research Group, Blanquerna School of Health Sciences, University Ramon Llull, 08025 Barcelona, Spain; Centro de Investigación en Ciencias del Deporte, Universidad Rey Juan Carlos, 28943 Fuenlabrada, Spain; Comité Paralímpico Español, 528040 Madrid, Spain; Tu Gestor de Salud, 28001 Madrid, Spain; Faculty of Food Technology, Autonomous University of Barcelona (UAB), Bellaterra, Spain; FC Barcelona Medical Department, FC Barcelona, 08028 Barcelona, Spain; Catalan School of Kinanthropometry, INEFC, 0838 Barcelona, Spain; Facultad de Ciencias de la Actividad Física, Deporte y Fisioterapia, Universidad Europea de Madrid, 28670 Villaviciosa de Odón, Spain; Facultad de Ciencias de la Actividad Física, Deporte y Fisioterapia, Universidad Europea de Madrid, 28670 Villaviciosa de Odón, Spain; Facultad de Ciencias de la Actividad Física, Deporte y Fisioterapia, Universidad Europea de Madrid, 28670 Villaviciosa de Odón, Spain; Faculty of Health Sciences, Catholic University of Ávila, 05005 Ávila, Spain; Biology Department, Universidad Autonoma de Madrid, 28049 Madrid, Spain; Facultad de Ciencias de la Salud, Universidad Alfonso X El Sabio, 28691 Villanueva de la Cañada, Spain; Facultad de Ciencias de la Salud, Universidad Alfonso X El Sabio, 28691 Villanueva de la Cañada, Spain; Departamento de Motricidad Humana y Rendimiento Deportivo, University of Seville, 41013 Seville, Spain; Department of Sports and Computer Science, Faculty of Sports, University of Pablo de Olavide, 41013 Seville, Spain; University of Granada, Campus of Melilla, 52005 Melilla, Spain; Departamento de Motricidad Humana y Rendimiento Deportivo, University of Seville, 41013 Seville, Spain; Departamento de Educación Física y Deportiva, University of Seville, 41013 Seville, Spain; Nursing Department, Faculty of Health Sciences, University of Alicante, 03690 San Vicente del Raspeig, Spain; LIFE Research Group, University Jaume I, 12071 Castellón de la Plana, Spain; Nursing Department, Faculty of Health Sciences, University of Alicante, 03690 San Vicente del Raspeig, Spain; Faculty of Health Sciences, Catholic University of Ávila, 05005 Ávila, Spain; Department of Health and Human Performance, Faculty of Physical Activity and Sports Sciences (INEF), Universidad Politécnica de Madrid, 28040 Madrid, Spain; Departamento de Nutrición y Bromatología, Facultad de Farmacia, Universidad de Granada, 18011 Granada, Spain; Faculty of Human and Social Sciences, Universidad Pontificia Comillas, 28049 Madrid, Spain; Departamento de Motricidad Humana y Rendimiento Deportivo, University of Seville, 41013 Seville, Spain; Studies Research Group in Neuromuscular Responses (GEPREN), University of Lavras, 37203-202 Lavras, Brazil; Departamento de Motricidad Humana y Rendimiento Deportivo, University of Seville, 41013 Seville, Spain; Studies Research Group in Neuromuscular Responses (GEPREN), University of Lavras, 37203-202 Lavras, Brazil

**Keywords:** female athlete, sport nutrition, macronutrient, micronutrients, supplement

## Abstract

**Context:**

Despite the progress toward gender equality in events like the Olympic Games and other institutionalized competitions, and the rising number of women engaging in physical exercise programs, scientific studies focused on establishing specific nutritional recommendations for female athletes and other physically active women are scarce.

**Objective:**

This systematic review aimed to compile the scientific evidence available for addressing the question “What dietary strategies, including dietary and supplementation approaches, can improve sports performance, recovery, and health status in female athletes and other physically active women?”

**Data Sources:**

The Pubmed, Web of Science, and Scopus databases were searched.

**Data Extraction:**

The review process involved a comprehensive search strategy using keywords connected by Boolean connectors. Data extracted from the selected studies included information on the number of participants and their characteristics related to sport practice, age, and menstrual function.

**Data Analysis:**

A total of 71 studies were included in this review: 17 focused on the analysis of dietary manipulation, and 54 focused on the effects of dietary supplementation. The total sample size was 1654 participants (32.5% categorized as competitive athletes, 30.7% as highly/moderately trained, and 37.2% as physically active/recreational athletes). The risk of bias was considered moderate, mainly for reasons such as a lack of access to the study protocol, insufficient description of how the hormonal phase during the menstrual cycle was controlled for, inadequate dietary control during the intervention, or a lack of blinding of the researchers.

**Conclusion:**

Diets with high carbohydrate (CHO) content enhance performance in activities that induce muscle glycogen depletion. In addition, pre-exercise meals with a high glycemic index or rich in CHOs increase CHO metabolism. Ingestion of 5–6 protein meals interspersed throughout the day, with each intake exceeding 25 g of protein favors anabolism of muscle proteins. Dietary supplements taken to enhance performance, such as caffeine, nitric oxide precursors, β-alanine, and certain sport foods supplements (such as CHOs, proteins, or their combination, and micronutrients in cases of nutritional deficiencies), may positively influence sports performance and/or the health status of female athletes and other physically active women.

**Systematic Review Registration:**

PROSPERO registration no. CRD480674.

## INTRODUCTION

Physical exercise, aimed at promoting health, preventing injuries, improving physical performance, or for leisure is a significant phenomenon in society. For example, last year 57.3% of Spaniards reported engaging in regular physical exercise, and the gender gap has narrowed (63.1% of men vs 52.5% of women). Women’s participation in physical exercise and sport has increased by over 20% since 2010.^1^ Additionally, at the international competitive level, the representation of women in the Olympic Games has risen from 34% in Atlanta 1996 to 48% in Tokyo 2020, and there is a commitment to achieve gender equality (ie, 50%) in Paris in 2024.^2^

Boys and girls exhibit similar physical conditioning until adolescence, when sexual maturation causes divergence. Male gonads increase the synthesis of inhibin (a protein) and testosterone (a hormone), which together play a significant role in sex-based differences.^3^ Testosterone stimulates a reduction in fat mass and an increase in muscle anabolism,^3^ making muscle more responsive to hypertrophy and muscle fiber strength.^4^ Girls, on the other hand, experience an increase in the production of 2 hormones: progesterone (only in the second post-ovulatory phase of the ovarian cycle and during pregnancy) and estradiol (which promotes fat accumulation in synergism with insulin). These hormonal differences result in adolescent and adult women having higher levels of adipose tissue, more noticeably in the chest and gluteo-femoral area.^3^ Epstein et al^5^ found there was 50% less muscle tissue in the upper limbs and 30% less in the lower limbs in women compared with men.

Anthropometric and hormonal differences make male athletes stronger, more powerful, and faster in explosive efforts, resulting in differences of over 30% in the world records in the heaviest weightlifting categories.^3^ However, in addition to hormonal differences, there are disparities between the sexes in mitochondrial function, substrate utilization, immune response, iron metabolism, thermoregulation, hydration, appetite control, energy availability, and endocrine function.^6^ On the other hand, women have a higher proportion of type I muscle fibers and more favorable mitochondrial function, which could be advantageous in endurance and ultra-endurance sports.^7^ This higher proportion of type I muscle fibers could explain the lower difference in muscle strength for lower limbs than for upper limbs.^8^ Compared with men, women oxidize more lipids and fewer carbohydrates (CHOs) and amino acids at the same exercise intensity.^3^ When comparing world records in swimming, there is a sex-based difference of 13.2% in the shortest distance (50 m) and of 5.7% in the longest distance (1500 m).^3^

To establish the optimal training loads for and nutritional requirements of female athletes, it is essential to consider the many differences in this population during their reproductive years and throughout the menstrual cycle (MC).^9^ During the first half of the MC (the follicular phase), estrogen levels progressively increase, reaching a peak that precedes the pre-ovulatory phase, during which follicle-stimulating hormones and luteinizing hormones reach their highest levels. The second half of the MC (the luteal phase) is characterized by a decrease in estrogens to their lowest levels and an increase in progesterone levels; a second peak of estradiol levels occurs before a reduction at the end of the MC.^10^ These hormonal variations throughout the MC can influence the female athlete’s metabolism. Estradiol promotes the bioavailability of fatty acids and lipolysis, while reducing the rate of gluconeogenesis, whereas progesterone: (i) decreases CHO sensitivity, mediated by IGF-1 in the muscle, and reduces liver glycogenolysis; and (ii) increases protein catabolism, with higher protein oxidation occurring both at rest and during exercise.^6^ Over time, the hormonal response in the luteal phase diminishes, and during menopause the estradiol to progesterone ratio becomes imbalanced, resulting in insulin resistance and anabolic resistance in the muscle, decreased muscle mass and bone mineral density (BMD), and an increase in fat mass.^11^

A well-planned diet can optimize health and the physical and cognitive performance of athletes.^12^ Optimal strategies for sports nutrition include dietary manipulation and supplementation strategies.^13^ Intrinsic characteristics of female athletes lead to specific responses to various training protocols and nutrition strategies, both of which can interact with the response and adaptions to physical exercise. However, it has recently been reported that only 6% of the studies focusing on the effects of CHO supplement strategies in athletes have included women in their sample populations.^14^ Another study found that, among studies assessing with a high level of scientific rigor the effects of dietary supplements on response and adaptions to physical exercise, those with samples composed exclusively of women represented somewhere between 0% and 8%.^15^ The present review is, to the authors’ knowledge, the first systematic review that has analyzed the effects of various nutritional and dietary strategies (including dietary manipulation and dietary supplementation) on performance, recovery, and health status parameters in female athletes and other physically active women.

## METHODS

### Protocol and registration

This systematic review has been prepared in accordance with the Preferred Reporting Items for Systematic Reviews and Meta-Analyses (PRISMA) guidelines (Supplementary Material S1).^16^ The current protocol was registered in the International Prospective Register of Systematic Reviews (PROSPERO) database (no. CRD480674).

### Literature search strategy

A systematic literature search was conducted using the following databases: Pubmed, Scopus, and Web of Science, including all the results published between January 1, 2000 and July 3, 2023. The search strategy was restricted to studies published in English, Portuguese, or Spanish, excluding manuscripts published in other languages. The search was conducted using keywords connected by Boolean connectors. The search strategy used in the Pubmed and Scopus databases was as follows: (concept 1) Women OR female AND (concept 2) sport OR exercise OR athlet* OR “physical active*” AND (concept 3) nutri* OR micronutrients OR macronutrients OR aminoacids OR “ergogenic aid” OR CHO OR carbohydrates OR glucose OR lipids OR fats OR proteins OR minerals OR fiber OR vitamins OR Diet* OR probiotics OR prebiotics OR symbiotics* OR nutraceutic* OR “functional foods” OR supplement* OR “non-nutrient” OR phytochemical AND (concept 4) “health status” OR healthy OR “sport performance” OR conditioning OR “muscle performance” OR endurance OR strength OR speed OR agility OR “international concurrence” OR competition OR biochemical OR physiolog* OR contest OR “sport biomarker” OR DOMS OR “hormonal behaviour” OR “nutritional status” OR “bone health” OR hematolog* OR “oxidative stress” OR inflammat* OR microbio*. This search strategy was adapted for the Web of Science database. In Supplementary Material S2, the full search strategy used in the 3 databases can be found.

### Eligibility criteria

The research question for this systematic review was: “What dietary strategies, including both dietary and supplementation approaches, can improve performance and/or health status in female athletes and other physically active women? In seeking to answer that question, inclusion criteria were established based on the PICOS criteria (see Table 1).^17^

**Table 1.** PICOS Table for Inclusion of Studies

Population	(i) The sample must exclusively consist of women.(ii) The individuals within the sample should be explicitly referred to as “female athletes” in the manuscript or meet the criteria of being classified as “physically active” or exceeding the exercise recommendations of the American College of Sports Medicine (ACSM).
Intervention	Inclusion of a dietary/nutritional intervention that should involve the manipulation of a macronutrient, a micronutrient, water, and/or the use of supplements
Comparison	Inclusion of either a placebo or control group (parallel group study design) or experimental condition (crossover study design)
Outcomes	Any parameter related to sports performance and/or health status
Study design	Randomized controlled trials

### Study selection

Covidence systematic review software was employed for the screening and recording of decisions. As a part of this process, 2 authors (I.L.-L. and E.C.) independently reviewed all the titles and abstracts identified in the search in the 3 databases. These reviewers excluded all manuscripts that did not align with the objectives of this review. In a subsequent phase, another 2 authors (J.M.-L. and L.G-R.) thoroughly revised the full texts and checked whether the manuscript fulfilled all the inclusion criteria. During the first (title and abstract screening) and second phases (full-text review), a third reviewer (A.S.-G.) was consulted to assist in resolving any discrepancies. Finally, manuscripts that included only a probabilistic magnitude-based inference were removed.

### Data extraction

Two authors (A.J.S.-O. and A.L.-S.) extracted data independently using the Covidence systematic review software (https://www.covidence.org/). Any discrepancy was resolved through discussion, and a third author (R.D.) was consulted if consensus was required. The information from each study that was included in the systematic review consisted of the following:

- Sample (trained or competitive athletes and other physically active women): (i) the number of participants and their main characteristics related to their sport practice; (ii) age of participants
- Menstrual function: (i) information about menstrual function (eumenorrheic, eumenorrheic, or menopause); (ii) information about possible control for the menstrual phase (follicular and luteal phases, with possible subdivisions of early, mid, or late phases in both the follicular and luteal phases) for the trials or assessment sessions
- Experimental group (parallel groups study design) or experimental conditions (crossover study design): (i) definition of each experimental group/condition; (ii) sample size of each experimental group/condition
- Intervention: (i) nomenclature of each intervention; (ii) characteristics of each experimental intervention; (iii) duration (acute: 1 day; days: <1 week; weeks: ≥1 week)
- Outcomes: all the outcomes assessed related to performance and/or health status
- Results: the outcomes specified for which statistical differences were found: (i) between experimental conditions (crossover study design); (ii) for the time of each group and in the interaction time-intervention (parallel groups study design).

### Risk-of-bias assessment

For this systematic review, the modified Cochrane risk-of-bias for randomized trials tool (RoB 2) was used.^18^ This scale, which is included in the Covidence tool, was employed to assess the potential risk of bias in each study. Based on the major domains of bias, the risks of bias were categorized as “low risk,” “unclear risk,” or “high risk.” Two authors (J.J.R.-A. and R.I.) conducted independent assessments of the quality of the studies and evaluated the risk of bias. Any disagreements between the reviewers were resolved by consultation with a third researcher (A.C.). The risk of bias was considered moderate, mainly for reasons such as a lack of access to the study protocol, insufficient description of how the hormonal phase during the menstrual cycle was controlled, inadequate dietary control during the intervention, or a lack of blinding of the researchers.

## RESULTS

### Literature search and study selection

**Figure 1.**
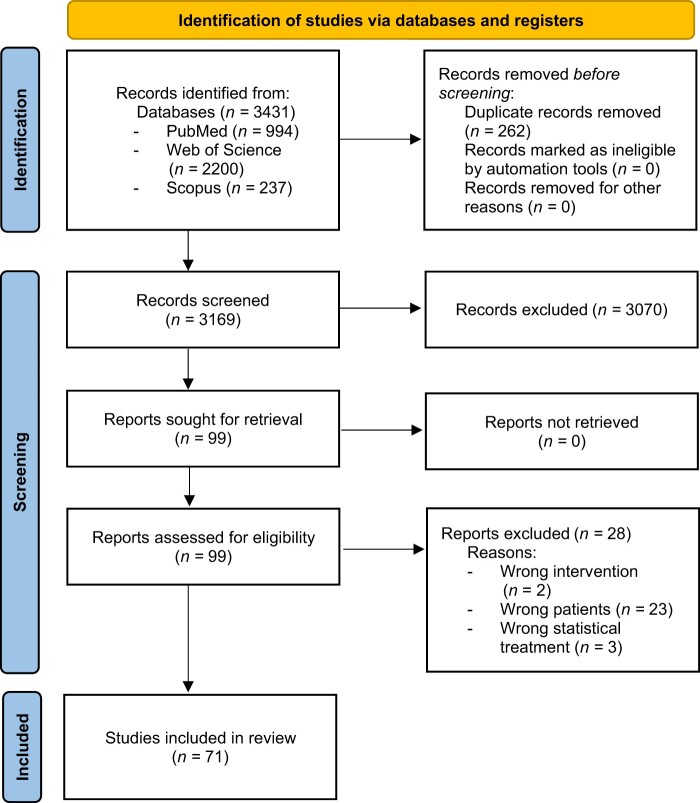
PRISMA Flow Diagram

A flow diagram illustrating the article selection process is provided in Figure 1. Initially, a total of 3431 articles were identified, which were trimmed to 3169 articles after eliminating duplicates (*n* = 262). Subsequently, a total of 3169 titles and abstracts were screened. Of these, 99 full-text articles were identified as being potentially relevant to this study. However, 25 manuscripts did not fulfil the eligibility criteria. A total of 74 articles met all the inclusion criteria, but 3 were removed based on the statistical analysis used. Hence, 71 articles were finally included in this systematic review.

### Characteristics of the studies included in the systematic review

Seventeen studies were focused on the analysis of dietary interventions: 7 of these studies had a parallel group design^18–25^ and 11 had a crossover design.^26–36^ The total sample size for these studies was 393 participants. Among them, 102 participants (26.0%) were categorized as competitive athletes, 65 were categorized as being highly or moderately trained (16.5%), and 226 (57.5%) were categorized as physically active or recreative athletes. Some studies^19^^,^^23^^,^^26–31^^,^^33^ included a sample group consisting of eumenorrheic participants, although 1 of these studies did not control for MC variations during trials^30^; 1 study specifically selected oligomenorrheic and amenorrheic women,^24^ another was focused on menopause,^23^ and the remaining studies did not control for menstrual function.

Of the studies focused on dietary manipulations, 6 were focused on analyzing the effects of various levels of dietary CHO ingestion^26–28^ or pre-exercise meals,^32^ including the impact of the glycemic index (GI),^30^^,^^31^ on health or performance outcomes. Two studies specifically analyzed the effects of dietary manipulation on CHO bioavailability.^19^^,^^29^ In relation to pre-exercise meals, 2 studies quantified the effect of calcium-enriched meals,^34^^,^^35^ while another quantified the effect of oatmeal ingestion.^36^ The effect of a high-protein diet was assessed in 3 studies.^20–22^ Other studies assessed the effects of a hyperenergetic diet,^24^ a diet enriched in polyunsaturated fatty acids (PUFAs) in combination with resistance training,^23^ or a high-antioxidant diet.^25^ Another study evaluated the use of milk as a dietary source enhancing recovery.^33^

A total of 53 studies were focused on the analysis of dietary supplement interventions. Regarding the type of experimental design, 20 adopted a parallel group design^37–56^ and 30 followed a crossover design.^57–86^ The total sample size of these studies was 1261 participants. Among them, 436 women (34.6%) were categorized as competitive athletes, 476 were categorized as being highly or moderately trained (37.7%), and 349 (27.7%) were categorized as physically active or recreational athletes. Twelve studies^40^^,^^44^^,^^49^^,^^51^^,^^61^^,^^64^^,^^65^^,^^69^^,^^72^^,^^84^^,^^87^^,^^88^ included a sample population of eumenorrheic participants. However, 3 of these studies did not control for MC variations in the trials.^64^^,^^73^^,^^74^ One study selected specifically menopausal women,^89^ and the other studies did not control for menstrual function.

According to Maughan et al,^87^ supplements can be classified into those used to prevent or treat deficiencies, those used as a practical source of energy and nutrients, and those used to directly or indirectly improve sports performance.

In the present review, 6 studies^37–42^ were classified as investigating the prevention or treatment of deficiencies: 3 studies analyzing the effects of iron supplementation,^37–39^ including 1 investigating the effects of iron plus symbiotic supplementation^38^; 1 study analyzing the effects of folic acid supplementation^40^; and 2 studies on the isolated and combined effects of vitamins C and E.^41^^,^^42^

We classified a total of 7 studies as investigating the effects of supplements used as a practical source of energy and nutrients^44–47^^,^^57^^,^^58^: 2 studies focused on the effect of α-lactalbumin^58^; 3 studies focused on protein supplementation,^44–46^ 1 of them in combination with creatine^45^; 1 study assessed the effects of various types of CHOs;^43^ and 1 study compared the effects of supplementation with CHO vs supplementation with a combination of CHO and protein.^57^

The majority (28) of the studies focused on dietary supplements directly aimed at improving performance.^48–52^^,^^59–78^ Among these, 10 studies investigated the potential effects of caffeine on sports performance.^59–68^ Five studies explored the effects of supplementation with nitric oxide (NO) precursors^72–76^ and β-alanine,^50^^,^^51^^,^^88–90^ and other supplements such as creatine,^48^^,^^49^^,^^70^^,^^71^ p-synephrine,^69^ sodium bicarbonate,^77^ or multi-ingredient formulations.^52^^,^^78^

Finally, 12 studies were undertaken to assess the effects of dietary supplements that might indirectly enhance performance.^53–56^^,^^86^ In this category, colostrum bovine was the most studied supplement (4 studies),^80–83^ with the remainder of the studies exploring the effects of other supplements such as fish oil,^54^ probiotics,^53^ curcumin,^85^ avocado pulp,^79^*Opuntia ficus indica*,^84^ tart cherry,^55^ chromium picolinate,^56^ and melatonin.^86^

### Summarized results of the studies based on the manipulation of diet

In Supplementary Material S3, the characteristics and main results of the studies with interventions based on dietary manipulation are summarized. Among the studies that manipulated CHO ingestion in the diet, 1 study reported an increase in glycogen level pre-exercise, enhancement of CHO oxidation during exercise, and higher net glycogen consumption, leading to an improved result for time to exhaustion (TTE) above 80% VO_2max_.^26^ Another study reported a selective effect on CHO oxidation in the follicular phase of the MC.^28^ In contrast, Dolins et al^27^ did not find differences in scores on a high-intensity endurance test when comparing groups of participants with differing CHO content in their diets, and Wynne et al^32^ did not find significant differences in participant performance with a higher CHO content in the meal previous to a simulated match, compared with a control group. Regarding GI, it has been reported that a high-GI dietary meal pre-exercise favors CHO oxidation at submaximal intensities.^30^^,^^31^ Additionally, it has been reported that the intake of oatmeal with milk pre-exercise could diminish the level of reactive oxygen species (ROS) post-exercise,^36^ and no gut discomfort or differences in performance were reported when compared with the effects of ingestion of a pre-exercise meal fortified in calcium.^34^^,^^35^ Furthermore, a diet with very low CHO bioavailability (ie, a ketogenic diet), has been reported to positively influence body composition (reduction of percentage fat mass and increasing lean body mass), but not neuromuscular adaptations. Similar results were found when physically active participants undertook training in a fasted state.^29^

Studies focused on a high-protein diet have not found an interaction between the duration of the intervention and performance variables, but they have confirmed that such a diet does not have a negative impact on health.^20^^,^^22^ However, it has been reported that a high-protein diet (2.0 g/kg/day) has more beneficial effects than a low-protein diet (1.0 g/kg/day).^21^ Similarly, it has been reported that a high-PUFA diet was associated with an increase in the cross-sectional area of muscle fibers, compared with a control diet.

Other interventions, such as a high-antioxidant diet^25^ or a hyperenergetic diet in athletes with oligomenorrhea and amenorrhea^24^ have not yielded positive results. On the other hand, milk consumption (355 ml) ingested before sleep has been reported to influence the metabolic response at submaximal intensities, with a potentiation of CHO metabolism.^33^

### Summarized results of the studies based on the manipulation of dietary supplements

The characteristics and main results of the studies with interventions based on dietary supplements are summarized in the Supplementary Materials, including supplements (i) for preventing and treating deficiencies (Supplementary Materials S4), (ii) as a practical form of energy and nutrients (Supplementary Materials S5), and (iii) for directly (Supplementary Materials S6) or (iv) indirectly improving performance (Supplementary Materials S7). Of the studies investigating dietary supplements for preventing or treating deficiencies, 3 studies that focused on iron supplementation reported a positive effect from this supplement in terms of increasing hemoglobin levels^38^ and log serum ferritin^39^ in competitive athletes. Additionally, a positive effect on total energy expenditure during 4-km time trials (TTs) was observed in iron-depleted rowers after iron supplementation.^38^ These positive effects on the main iron metabolism indicators (ie, hemoglobin and soluble transferrin receptor) have also been observed in soldiers after an intensive training program, when they had been pretreated with iron supplementation.^37^ Regarding vitamin supplementation, folic acid may be associated with a small enhancement of blood flow activity,^40^ while vitamins with antioxidant effects, such as vitamins C and E, have failed to show enhancement of physical performance or facilitation of recovery post-exercise.^41^^,^^42^

Regarding the use of supplements for supporting energy and nutrient bioavailability for physical exercise, it has been reported that CHO ingestion during the acute recovery phase post-exercise that induces muscle glycogen is effective for increasing performance in subsequent maximal efforts.^43^ It has been reported that intake of a combination of CHO and protein during exercise could have more beneficial effects than intake of CHO alone, for TTE at 75% VO_2max_ after 3 hours of submaximal cycling.^57^ Based on the results obtained from 3 studies, ingestion of egg (a natural form of protein supply)^44^ and whey protein post-exercise at low dosages (from 15 to 25 g)^45^^,^^46^ could be effective in improving both physical performance and body composition (in terms of increased lean body mass). In 2 studies conducted with α-lactalbumin supplementation, positive effects have been reported in competitive athletes regarding variables related to sleep quality^47^^,^^58^ as well as endurance performance.^58^

Regarding supplements for enhancing performance, the most important finding is that 9 out of 10 studies analyzing the effectiveness of caffeine (at dosages ranging from 3 to 6 mg/kg of caffeine administered 45 to 60 minutes pre-exercise) have reported an ergogenic effect for at least one of the assessed physical variables,^59–62^^,^^64–68^ especially in explosive and high-intensity efforts. Regarding the MC, the 2 studies published have reported an ergogenic effect from caffeine ingested during both the early and late follicular phases,^59^^,^^60^ but 1 of the studies did not find statistically significant differences in the luteal phase.^61^ Similarly, greater effects of caffeine have been observed in the morning,^66^ while the ergogenic effects appear similar at dosages ranging from 3 to 6 mg/kg,^67^ with no additional efficacy reported after combining with CHO mouth rinsing.^68^ However, 1 study reported a negative effect of caffeine on sleep post-supplementation.^64^

Regarding NO precursors, a study performed with elite hockey players failed to show enhancement in physical conditioning in a simulated match after beetroot juice supplementation (BRJ).^74^ However, the other 4 studies conducted in physically active or moderately trained women reported ergogenic effects of BRJ^72^^,^^73^ and citrulline malate^75^^,^^76^ in explosive and high-intensity efforts. Regarding alkalizing supplements, an acute study using supplementation with sodium bicarbonate (${\text{NaHCO}}_{3}^{-}$) found there was increased pH and ${\text{NaHCO}}_{3}^{-}$ in the blood of elite water polo squad players, but it was not effective in enhancing performance outputs in a simulated match.^77^ Three out of the 4 studies conducted using β-alanine as a supplement reported an ergogenic effect on physical performance, including endurance,^51^ high-intensity efforts,^50^^,^^75^ and explosive effort.^75^

Regarding creatine supplementation, a study of less than 1 week of supplementation duration failed to show an incremental improvement in physical performance,^49^ while longer studies reported effects on body composition, including an increase in body mass and/or lean body mass, with^70^ or without^71^ concurrent increases in physical performance. When creatine supplementation was combined with plyometric training, a greater effect was observed compared with only plyometric training.^48^

Other studies have reported conflicting results regarding the effectiveness of multi-ingredients supplements,^52^^,^^78^ and 1 study has reported non-ergogenic effects of p-synephrine.^69^

Regarding dietary supplements with a potential indirect effect on improving performance, colostrum bovine supplement has been assessed in 4 studies conducted on high-level athletes.^80–83^ One of these studies reported that this supplement had a positive effect on plasma buffer capacity in elite women rowers,^82^ and a second study in the same sport reported increased mechanical work done and performance in a 4-minute rowing TT.^83^ The other 2 studies, focused on inflammatory and iron metabolism markers, reported that colostrum bovine supplement had an ameliorating effect on the inflammatory response to exercise: a reduction in IL-6^81^ and creatine kinase in 1 study, and an increase in IL-10^80^ in the other, post-exercise. Other effects on the inflammatory response post-exercise have been investigated in a further 3 studies: increased performance was associated with some supplements (eg, curcumin supplementation was associated with increased VO_2max_–),^85^ but not with other supplements, such as fish oil^54^ or *Opuntia ficus indica*.^84^ Additionally, ergogenics effects have been reported for both the probiotic DE111^53^ and tart cherry.^55^ In this way, probiotic DE111^53^ increased strength andinduce to a reduction of body fat mass while tart cherry supplementation^55^ induce to higher neuromuscular function in physically active women.^53^ The intake of avocado pulp has been shown to enhance recovery post-exercise.^79^ Conversely, other supplements such as chromium picolinate^56^ and melatonin^86^ have not demonstrated similar effects.

### Risk-of-bias assessment

Overall, the experiments exhibited “unclear” risk of bias. Our assessments predominantly indicated unclear risk of bias for 55 studies (77%), while 12 studies fell into the category of high risk (17%) and 4 studies (5%) exhibited a low risk of bias (see Supplementary Material S8). This was primarily due to a lack of reporting of protocols prior to the trials. Without this issue, the majority of interventions would have scored a low risk of bias. Among the 71 RCTs, 10 studies had an unclear risk of bias related to lack of a dietary control group; 40 studies were assessed as having risk of bias related to absence of information about controlling for MC phase; 25 studies were assessed as having risk of bias related to a lack of information on the protocols; finally, 17 studies were assessed as having risk of bias related to the blinding of researchers. Generally, the studies scored better (were assessed as having “low risk”) on items related to deviations from the intended interventions, missing outcome data, and participant randomization.

## DISCUSSION

The first finding of this systematic review was that there is a very small number of studies that have been undertaken focused on optimizing health, performance, and recovery in female athletes and other physically active women, relative to the number of studies focused on male athletes. Thus, dietary strategy recommendations based on scientific evidence for women^13^^,^^87^^,^^91–93^ are currently overshadowed by those available for physically active men athletes. The second important finding was that only 22 studies (31%) (including 2 studies with menopausal/oligomenorrheic/amenorrheic athletes) considered the MC when undertaking trials, and only 4 studies compared the effects of various nutritional interventions at different phases of the MC. Due to the limitations of the available data, the current dietary recommendations for female athletes (including those discussed in the present systematic review) may have limited biological validity and applicability in physically active women and female athletes.^15^ The American College of Sports Medicine declared that the benefits of dietary supplements should be considered as an adjunct to a well-designed nutritional plan, as a possible optimizing complement.^13^ The dietary recommendations for female athletes, however, represent a starting point in new lines of research that may provide information about the physiological effects of dietary manipulation and supplementation, and the best way to apply dietary manipulation and supplementation, within the framework of female physiology and adapted to the various sports disciplines. Nevertheless, 76% of the studies included in the present systematic review assessed the effects of dietary supplements.

### Effects of various types of dietary manipulation in female athletes

In the 1970s, a pioneering study established a direct relationship between fatigue, as determined by TTE running at an intensity of approximately 80% VO_2max_ in both continuous and intermittent procedures, and the complete depletion of muscle glycogen stores.^94^ In a subsequent study, a dual relationship was established: (i) higher CHO content in the diet resulted in higher muscle glycogen content; (ii) higher pre-exercise muscle glycogen content improved performance for TTE running at approximately 80% VO_2max_.^95^ These studies are of considerable importance, as their findings showed the benefit of increasing the CHO content of an athlete’s diet. In fact, the initial recommendations for an athlete’s nutrition prioritized the establishment of a high-CHO (HCHO) diet. Athletes were advised to increase their CHO consumption based on their energy cost of training or competition, environmental factors, and sex (with men requiring a higher amount of CHO than women).^96^

For decades, CHOs have played an important role in the dietary and nutritional interventions and planning for athletes. Over time, various recommendations for CHO intake have been established, based on the type and duration of training and competition. In recent years, there has been a growing interest in the application of CHO manipulation strategies as part of nutritional prioritization and as a key element of training planning.

Examples of such strategies are HCHO or low-CHO diets, fasting, or GI-based diets. These strategies have been relatively understudied in female athletes. Glycemic index is a term used to classify dietary sources of CHO, based on the rate at which blood glucose level is increased after ingestion, compared with the rate after ingesting glucose.^97^ A study was conducted by Stevenson et al^30^ in 8 active and eumenorrheic women. They compared the effects of pre-exercise mixed meals containing CHOs with either a high GI (HGI) or a low GI (LGI) on substrate utilization during rest and exercise. A breakfast was provided 3 hours prior to exercise, including 2 g of CHOs per kilogram of fat mass, with the GI for one group being 78 (HGI) and that of the other group being 44 (LGI). Their results demonstrated that GI can influence substrate oxidation during subsequent exercise: there was a higher rate of fat oxidation in the group that ingested a LGI breakfast. In addition, GI was found to have a significant effect on hyperglycemia and hyperinsulinemia response (higher in HGI than LGI) during the postprandial period in women.

One of the studies included in this systematic review confirmed this pioneering finding in the field of sports nutrition. Walker et al^26^ found that 3 days following a HCHO diet, followed by 3 days with a large volume of training while following a mixed diet (48% of energy provided by CHO), during the luteal phase, increased muscle glycogen content before exercise in comparison with a mixed diet alone in female endurance athletes. Furthermore, the HCHO diet induced a metabolic response characterized by an increased respiratory exchange ratio (RER), increased rate of CHO oxidation, higher blood lactate concentration, and an extended TTE at an intensity of above 80–82% VO_2max_. These results substantiate for women the previously reported relationship established for male athletes between the CHO content of the diet and fatigue in efforts where glycogen depletion is a limiting factor of fatigue.^94^^,^^95^

In another study, Dolins et al^27^ analyzed the effect of diets with different CHO content (3 vs 5 vs 8 g/kg/day) on TTE at an intensity of 90% VO_2max _in female endurance cyclists. They did not report differences in the metabolic response, measured as either RER or in performance. The absence of change in the metabolic response being detected in that study is expected, because the intensity used (upper second ventilatory threshold) can only be sustained through CHO metabolism.^98^ The intensity of the effort was modulating the metabolic response more significantly than the muscle glycogen content was. The relatively short duration of the effort (∼4 minutes) meant that glycogen availability was not a limiting factor for performance. In fact, these results could be in agreement with the findings of McLay et al,^28^ who investigated muscle glycogen levels pre-exercise (following ingestion of 8.4 vs 5.2 g/kg/day of CHO) and found a subsequent increase in the rate of CHO utilization during submaximal exercise in moderately trained female cyclists (at intensities of between 45% and 75% VO_2max_). The McLay et al^28^ study did not demonstrate a statistical significant enhancement of performance in the time taken to cover a 16-km TT, although 6 out of 8 participants showed an improvement in performance after ingesting a HCHO diet. Their study validated 2 hypotheses: (i) HCHO diets increase muscle glycogen and the metabolic use of CHO as a fuel source, and (ii) an increase in muscle glycogen content after a HCHO diet is not effective in improving performance in trials characterized by a lack of depletion of muscle glycogen content. They found that neither the metabolic nor the physical response differed between the midfollicular and midluteal phases; however, the most important finding was that HCHO diets do not increase performance during physical efforts where there is an insufficient capacity for depletion of muscle glycogen stores in female athletes.

Zeng et al^36^ investigated the influence of CHO ingestion in the meal preceding exercise and reported a positive effect of including oatmeal (1 g/kg) combined with milk, in comparison with only milk. There was an increase in blood glucose during high-intensity interval training (HIIT) and a reduction in ROS post-exercise. These effects may be attributed to greater glycogen availability during exercise, which could reduce metabolic stress in the acute recovery period post-exercise. Oatmeal is a dietary source enriched in CHO with a low GI (LGI). Two different studies have reported the effects of an isocaloric breakfast (2 g/kg/day) consumed 3 hours pre-exercise, comparing the effects of LGI and HGI meals.^30^^,^^31^ In one of these studies, which involved 7 physically active women, the HGI meal resulted in an increase in blood glucose and insulin levels pre-exercise, with a trend towards increased CHO metabolism.^31^ The second study, conducted with a large sample (28 participants), confirmed this response to the HGI meal pre-exercise and reported a statistically significant increase in the rate of CHO utilization and blood glucose levels during 60 minutes of running at 65% VO_2max_.^30^ In another study of the effects of a meal intake pre-exercise on physical performance, Wynne et al^32^ compared the effects of an isocaloric ingestion of 2 g/kg vs 4 g/kg of CHO on performance in a simulated match, consisting of 2 halves of 35 minutes each, involving national soccer players. They did not report any statistically significant difference after the HCHO meal pre-exercise. Taking into account that, during the days preceding the trials, participants ingested a low-CHO or a HCHO diet, and the duration of the trial, this study confirmed that an increase in CHO ingestion in a pre-exercise meal was not effective for enhancing performance.^32^ However, it was effective in modulating metabolic responses, with a prioritization of CHO metabolism.^30^

Breakfast provides a good opportunity for athletes to incorporate dairy into their diets. Dairy products are rich dietary sources of calcium, a mineral essential for female athletes, not only for structural bone health but also for blood clotting, muscle contraction, nerve transmission, protein utilization, and cellular communication. Calcium regulation is controlled by parathyroid hormone, calcitonin, and vitamin D. The ingestion of calcium before exercise may help to attenuate bone resorption during exercise, particularly considering potential calcium losses through sweat.^99^ To investigate the impact of calcium intake in the meal pre-exercise on calcium body metabolism, Haakonssen et al^35^ assessed the effect of a calcium-rich dairy-based meal (1350 mg of calcium) consumed in a breakfast in the 90 minutes prior to exercise. They reported a positive effect on ionized calcium and a reduction in parathyroid hormone concentrations. The practical implications include a possible positive effect on markers of bone resorption after a high-calcium dairy-based pre-exercise meal.^35^ In another study, it was reported that a fortified calcium–rich, dairy-based breakfast consumed during the 3 hours prior to exercise did not adversely affect physical performance or cause gut discomfort in female competitive cyclists.^34^ This suggests that, to satisfy the calcium recommendations for female athletes, dairy products could be a suitable dietary source. Investigating the effects of calcium dietary sources, Ormsbee et al^33^ introduced a different perspective by exploring the ingestion of chocolate milk before bedtime and its impact on morning metabolism, hydration, and performance in female runners and triathletes. The results of their investigation showed a modification of metabolism at rest and during morning exercise, with the ingestion of cocoa milk favoring CHO oxidation, in comparison with the ingestion of a nonnutritive placebo. However, these effects did not translate into improvements in a 10-km running TT.

The results thus far described suggest that various dietary strategies can have either positive or null effects on physical performance, depending on their effects on metabolic response during exercise. Training periodization is designed on the basis of different principles, with the aim of summing accumulative responses across different training sessions to induce the most favorable adaptations. Given that the objective of endurance performance is the potentiation of lipid metabolism during training sessions, whether polarized or pyramidal training is used, high-volume low-intensity exercise is the most commonly used strategy and is supported by a considerable amount of scientific evidence.^100^ This metabolic objective of training led Noakes et al^101^ to propose a dietary strategy creating low bioavailability of CHO, inducing metabolic stress that is associated with enhancement of performance after a CHO loading before a competition, the so-called ‘train low, compete high’ strategy. Two studies within this systematic review have analyzed the effect of training with low CHO bioavailability.^19^^,^^29^ One study was focused on intermittent fasting, which is characterized by periods of normal food and beverage consumption interspersed with periods of energy-intake suppression or fasting, and encompasses several specific fasting protocols.^101^ Martínez-Rodríguez et al^29^ found that HIIT over 8 weeks effectively reduced fat mass and skinfolds in physically active women, both with and without fasting. However, that study could not find any benefit from using intermittent training on physical performance assessed by the countermovement jump (CMJ) or the Wingate test. In the other study, Vargas-Molina et al^19^ reported that a ketogenic diet is more effective than a HCHO diet in improving body composition, specifically in decreasing body fat, after 4 weeks. However, the increase in physical performance was higher with the HCHO diet. Therefore, while the metabolic response to a diet with low CHO bioavailability could be effective for reducing body fat and for potentiation of lipid metabolism, physical adaptation could be hindered by suboptimal intensity during high-intensity training sessions.

Protein intake manipulation strategies in athletes aim to optimize all processes associated with sports practice, as a higher protein intake in the 24 hours following exercise appears to enhance exercise-associated muscle protein synthesis and may lead to a chronic increase in muscle protein as well as muscle functional changes. These potential adaptations may be attributed to increased leucine concentrations and exogenous amino acid delivery, stimulating protein synthesis.^13^ Three studies included in this systematic review have assessed the effect of manipulating dietary protein ingestion.^20–22^ Arciero et al^21^ compared the effects of high-protein dietary intake (2 g/kg/day) and low-protein dietary intake (1 g/kg/day) during multi-mode exercise training. To optimize the anabolic effect of the protein, the 2 g/kg/day protein ingestion was divided into ingestions of 0.25 g/kg distributed across 5–6 meals over the day. This distribution strategy was based on the fact that 25 g of protein of high biological value exceeds the blood leucine levels that activates the signaling pathway for myofibrillar protein synthesis.^102^ Thus, the strategy of ingesting meals including greater than 0.3 g/kg of protein (>18 g for an athlete of 70 kg of body mass) in each meal was proposed in order to amplify the anabolic effects of protein.^103^ The study carried out by Arciero et al^21^ confirmed experimentally that the distribution of daily protein in intakes of approximately 25 g of protein, at a dosage in the upper limit of the recommendations for athletes (1.2–2.0 g/kg/day),^13^ is effective in amplifying the effects of training on physical performance.

In a second study, Cambell et al^22^ reported the positive effects of ingesting separate meals with a protein content of 25 g (reaching up to 2.4 g/kg/day) in response to an 8-week resistance training program in resistance-trained female athletes. This approach proved effective for increasing both strength and lean body mass. Considering the potential impact of a high-protein diet on bone metabolism, Antonio et al^20^ studied the safety of a high-protein diet (>2.2 g/kg/day). The results of that study confirmed that a protein intake of 2.5 times higher than the recommended amount for trained women did not negatively affect BMD or content.

Lipids are the least well-studied macronutrient for athletes; however, the impact of the various types of fatty acids (specifically omega-3 PUFAs, PUFA-n3s) on health parameters has been well established.^104^ PUFA-n3s, specifically eicosapentaenoic acid (EPA) and docosahexaenoic acid (DHA), are associated with several benefits for physical performance. These include: improving oxygen and nutrient uptake in skeletal muscle; reducing oxidative stress and inflammation; improving muscle recovery; regulating oestrogen levels; increasing protein synthesis, muscle hypertrophy and strength; and alleviating delayed onset muscle soreness (DOMS).^105^ A study included in this systematic review^23^ evaluated the effect of a high-PUFA-n3s dietary intervention on various sport-related metrics in women. The effect of a healthy diet rich in PUFA-n3s (≥500 g/week of fish and seafood and a n-6:n-3 ratio of <2 for 24 weeks) in combination with resistance training was compared with the effect of resistance training alone. The authors reported a significant increase in the hypertrophy of fast type IIA skeletal muscle fibres (+23%), an upregulation of mTOR in skeletal muscle and a downregulation of IL-1β, in comparison with the control group, who only received the resistance training and continued with their usual diet. These findings suggest that female athletes could benefit from a diet rich in PUFA-n3s, and a consumption of greater than 500 g/week of fish and seafood could have a positive impact on their training adaptions.

In an attempt to assess the effect of a high-antioxidant diet on eating behavior, body image, and mood of professional handball players, Miralles-Amoros et al^25^ investigated the effects of a diet focused on the inclusion of fruits high in antioxidants, such as blueberries, beetroot and pomegranate, providing 200% of the Recommended Dietary Allowance (RDA) for vitamins A, C, and E. They compared the outcomes with those for participants following an isocaloric diet that included other type of fruits with a lower content of antioxidant vitamins, providing 100% of the RDA for each vitamin. No significant differences were observed. However, it would be interesting to assess the influence of this type of diet on outcomes more related to health and exercise recovery, which may well be modulated by the consumption of antioxidant compounds.

Estrogen modulates calcium and bone metabolism. Thus, women with an alteration of the MC are at increased risk for low BMD that could induce to osteopenia and/or osteoporosis. Therefore, one of the more important objectives for oligomenorrheic and amenorrheic athletes is restoration of their habitual MC and an increase in their calcium intake (it is recommended that they increase daily consumption to 1500 mg/day).^13^ In view of the relationship between dietary energy intake and menstrual function, De Souza et al^24^ investigated the effects of a hyperenergetic diet (+20–40% above baseline energy needs) on oligomenorrheic and amenorrheic physically active women. They showed that this diet had a beneficial effect on body composition and normalization of IGF-1, leptin, and triiodothyronine levels; however, no effects were reported on BMD. These findings are very important, indicating that the negative effects on BMD of oligomenhorrea and amenorrhea cannot be reverted with a hyperenergetic diet.

### Effects of various dietary supplements in female athletes

The prevalence of dietary supplement intake for female athletes is reported to be above 70% in sports teams,^106^ racket sports,^107^ and endurance activities.^108^ Studies indicate that the main reasons for supplement consumption are increase in sports performance, followed by improvement in health status.^106–108^ Analysis of the studies included in this systematic review confirms that the majority of the published studies on female athletes are focused on dietary supplements taken with the primary aim of directly increasing sports performance.

Micronutrients play a crucial role in many physiological processes, significantly impacting, for example, energy metabolism, protein synthesis, and cell generation.^9^,^1^ Despite understanding of their importance, micronutrient deficiencies continue to be a global health problem, with iron deficiency (ID) disproportionately affecting women nearly twice as much as men.^20^ While it is nutritional deficiencies that are commonly associated with the developing world, women in developed nations also experience suboptimal micronutrient status. Exercise stresses many of the metabolic pathways in which micronutrients are required, and training may induce muscle biochemical adaptations that increase the need for specific micronutrients. Female athletes (especially those restricting energy intake, engaging in severe weight loss, eliminating entire food groups from their diet, adhering to extreme diets, or participating in intense physical activity) may require exogenous vitamin and mineral supplementation for health and performance maintainance.^13^ Female athletes should pay close attention to common deficiencies linked to their physiology, such as vitamin D, calcium, and iron deficiencies, which not only have a negative impact on sports performance, but also lead to health risks like osteoporosis and anemia. In addition, deficiencies in B vitamins and certain antioxidants (vitamins E and C) have been identified in female athletes.^109^

Iron is an essential mineral for energy metabolism, cell differentiation and proliferation, antioxidant defence, cognitive development, and immune response.^110^ Iron deficiency is the most prevalent deficiency in the athletic population, with a particularly high prevalence in female athletes (exceeding 57%), compared with the non-athletic female population.^111^ Increased iron demand in women is caused by menstruation, but female athletes increase their iron demand further due to microischemia produced during excessive training,^112^ increased hemolysis, excessive sweating, hematuria,^113^ and frequently not meeting the iron intake requirements due to the consumption of hypocaloric diets or foods with low iron bioavailability, especially in those adhering to vegetarian or vegan diets.^114^ Female athletes should be aware that prolonged ID could induce ID anemia (IDA). Even with normal hemoglobin levels, ID can reduce endurance capacity and athletic performance by affecting energy metabolism, decreasing the lactate threshold, decreasing VO_2max_ and causing early onset of fatigue.^115^

Research suggests potential benefits from daily or intermittent iron supplementation in female athletes. McClung et al^37^ undertook a study on a population of US servicewomen, reporting that daily iron supplementation alleviated training-related ID, improving the physical performance and cognitive status of women soldiers during basic combat training. Another study carried out by Dellavalle and Haas^38^ in female rowers with ID showed that iron supplementation during the training period improved their iron status and energy efficiency during endurance exercise. These findings are particularly relevant for endurance athletes, whose dietary patterns and physical training increase the risk of ID, and suggest that adequate iron supplementation can maximize the benefits of endurance training. In another study,^39^ an 8-week intake of symbiotic supplements (pre- and probiotics) or foods containing symbiotics, along with iron sulfate supplementation was shown to improve iron status in female athletes with ID. These results suggest recommending the use of symbiotics in athletes with medical prescription for iron supplements. Moreover, it is necessary to support dietary iron bioavailability by incorporating nutrients that facilitate iron absorption, such as vitamins A and C and the “meat” factor, and avoiding the simultaneous intake of foods containing factors that reduce iron absorption, such as calcium, fiber, and polyphenols. Additionally, processes that reduce iron bioavailability, such as freezing or prolonged cooking. should be minimized. It is also necessary to limit increase in hepcidin (caused by exercise at intensities of upper VT1, large amounts of iron, and inflammation), a hormone that reduces dietary iron absorption.^116^

Folate and vitamin B12 play important roles in red blood cell formation and cellular repair; therefore, suboptimal intake levels of these vitamins could affect the performance of female athletes. When dietary intake provides optimal amounts of these vitamins, additional folate and vitamin B12 supplementation do not provide additional performance improvements in female athletes.^13^ Another study included in this systematic review showed that folic acid supplementation may improve endothelial function in eumenorrheic female runners with borderline flow-mediated dilation and thus potentially reduce future cardiovascular risk.^40^ However, the results of that study should be interpreted with caution for 2 reasons: (i) the small sample size of the study (13 women); and (ii) the doses used (10 mg/day) in the intervention, which exceeded 10 times the tolerable upper intake level. It is important to note that high intakes of folate supplements might temporally “mask” vitamin B12 deficiency, leading to irreversible neurological consequences.^117^ Given that folic acid and vitamin B12 supplementation are intricate, further studies are warranted to determine the synergistic and non-antagonistic aspects of folic acid and vitamin B12 joint supplementation, taking into account different sports disciplines and age ranges of female athletes.

Antioxidant vitamins such as vitamins A, C, and E (especially vitamin C) may help female athletes to enhance their training tolerance by reducing oxidative damage and/or maintaining a healthy immune system during heavy training. However, high/supramaximal doses of these vitamins may hinder the intracellular adaptations caused by exercise training, creating a negative impact on an athlete’s performance. It is important to note, though, that such high/supramaximal doses are not typically achieved through a balanced and healthy diet.^93^ Two studies included in the systematic review investigated the effectiveness of large doses of vitamin C (250 mg/day), vitamin E (400 IU/day), and their combination in trained female athletes.^41^^,^^42^ The outcomes of these studies revealed no significant improvements in endurance performance (VO_2max_), body composition,^41^ or biomarkers of muscle damage^42^ following 4 weeks of strenuous endurance training involving 64 female athletes without vitamin deficiencies. These results are in line with the results reported by Miralles-Amoros et al,^25^ comparing outcomes for a high antioxidant diet (200% RDA of vitamins A, C, and E) with those for an isocaloric diet (100% RDA of vitamins A, C, and E).

Overall, the results of this systematic review support the hypothesis that micronutrient supplementation in female athletes without nutritional deficiencies does not have additional benefits or ergogenic properties,^13^ and could even be counterproductive to the adaptations sought through training. Recommended Dietary Allowance values may be adequate to cover the requirements of female athletes. It is important to note that nutritional deficiencies should be identified through a comprehensive nutritional assessment that includes an evaluation of dietary intake and of the appropriate urinary or blood markers.^118^

Dietary supplements can be used as a practical form of supporting energy and nutrient requirements in various sports contexts. These supplements, collectively named sport foods, encompass heterogeneous products marketed as sport or energy drinks, CHO and protein supplements, liquid meals, or electrolyte replacement supplements.^91^ CHO supplementation in the form of sport drinks and bars is widely consumed in various sports modalities by female athletes, due to their capacity to maintain stable blood glucose and delay the depletion of glycogen stores during exercise.^119^ In addition, post-exercise CHO ingestion enhances glycogenesis and aids recovery.^120^ For optimizing CHO supplement administration, CHO source supplementation (eg, glucose/fructose/sucrose) must be considered. Research suggests that a combination of CHO sources (eg, glucose + fructose + sucrose) is more effective than a single CHO source (glucose) for increasing absorption (∼1.7 vs 1.0 g/min).^121^ Moreover, a high-molecular-weight glucose polymer (HMW) has been shown to enhance the gastric emptying rate for 10 minutes post-ingestion and to promote post-exercise muscle glycogen re-synthesis, compared with isocaloric low-molecular-weight solution (LMW).^122^ In this systematic review, we only identified 1 study that has assessed CHO supplementation effects in the post-exercise period after exercise in which glycogen depletion was induced.^43^ In agreement with the study that reported the positive effect of a HCHO diet in efforts that induced muscle glycogen depletion,^26^ Mock et al^43^ in 13 competitive female cyclists, reported a higher performance in a 15-minute TT in a depleted muscle glycogen context induced by exercise after ingesting CHO, in comparison with a placebo; however, no differences were reported between the effects of HMW and LMW beverages.

Although CHO drinks are the most popular supplement chosen during exercise,^106–108^ a systematic review and meta-analysis concluded that the combined ingestion of CHO and protein leads to greater endurance performance in TTE compared with CHO intake alone. In a study identified within this systematic review, in a sample of 14 trained female cyclists, McCleave et al^57^ confirmed that supplementation with a low mixed CHO supplement plus protein (1% each of dextrose, fructose, and maltodextrin + 1.2% protein) was more effective than supplementation with a traditional 6% CHO supplement (6% dextrose only) in increasing TTE at 75% VO_2max_ after a period of 3 hours with intensities ranging between 45% and 75% VO_2max_. A plausible hypothesis to explain this effect is that protein may increase insulin production that induce to an increased glucose clearance from the blood and elevating the CHO oxidation rate, reducing exercise-induced muscle damage and/or maintaining Krebs cycle intermediates and oxidative energy production during long-distance efforts in women.^57^

In the immediate post-exercise period, protein intake increases protein synthesis 3-fold compared with basal situations. Therefore, this immediate post-exercise period has been established as a suitable time for ingesting proteins.^123^ One of the most suitable dietary protein sources for athletes in terms of quality and ease of consumption is protein supplements. Three studies identified in this systematic review examined the effects of protein supplementation in female resistance-training athletes.^44–46^ In one of these studies, Taylor et al^45^ observed, after an intervention involving 8 weeks of ingesting 24 g of whey protein following resistance training (4 times per week) in 16 female basketball players, that there were improvements in body composition (increase in lean body mass and reduction in body fat mass) and performance (one maximum repetition [1-RM] in bench press, and agility), compared with a CHO supplementation intervention (24 g maltodextrin). In another study, Wilborn et al^46^ conducted a combined interventional study lasting 8 weeks, with 4 sessions per week of resistance training and supplementation based on protein (25 g whey protein) or protein plus creatine supplementation (25 g whey protein+5 g of creatine monohydrate), reporting similar positive effects of protein supplementation on both body composition and physical performance, but no additional effects from creatine. On the other hand, Hida et al^44^ failed to find a significant effect of protein supplementation on body composition or physical performance after 8 weeks of daily supplementation with either protein or CHO (17.5 g maltodextrin). This discrepancy may be attributable to the protein supplementation procedure: the amount of protein supplementation in the study of Hida et al (15.0 g egg white protein) may have been insufficient for activating the signaling pathway for myofibrillar protein synthesis after protein supplementation; it is estimated that more than 0.25–0.3 g/kg of high-quality protein is required.^10^,^4^

Most elite athletes suffer insufficient sleep duration,^124^ and it seems that women may be particularly vulnerable to sleep deprivation, with more sleep disturbances and decreased sleep quality during the luteal phase of the MC.^125^ In this systematic review, 2 studies that analyzed the effect of supplementation with 40 g of α-lactalbumin protein powder before bedtime were identified, 1 investigating acute intervention^58^ and the other intervention over a period of 3 weeks.^47^ The results from both studies were promising, as they reported positive effects on sleep quality indicators. Additionally, one of the studies, which also assessed physical performance the following day, showed enhanced endurance performance after α-lactalbumin supplementation.^58^ However, these results should be taken cautiously, due to: the small sample sizes of the 2 studies (16 and 18 participants), the heterogeneity of the samples (which included athletes from soccer, rugby 7, field hockey, middle distance running, and powerlifting), variations in the use of oral contraceptives among participants, lack of an exhaustive control of dietary intake, and an absence of control for MC and/or hormonal fluctuations. It is necessary that further studies recognize the importance of considering and controlling aspects such as dietary intake variations, hormonal fluctuations, and the use of contraceptives.

The findings of this systematic review suggest that sports foods delivering CHO during exercise or between efforts, particularly in situations with muscle glycogen depletion, could be effective in increasing endurance performance and recovery, and possibly effective in enhancement of training adaptions in female athletes. Additionally, there may be a potential enhancement of adaptations induced by resistance training when protein supplementation exceeding 25 g of a high-quality protein (eg, whey protein) is ingested immediately post-exercise. In addition, future studies should analyze the potential beneficial effect of α-lactalbumin for enhancing sleep in female athletes.

There is a high level of scientific evidence for direct enhancement of sports performance by a select group of dietary supplements, including caffeine, creatine, alkalizing, and nitrate (${\text{NO}}_{3}^{-}$) supplements.^87^^,^^91^^,^^92^ Among these supplements, caffeine stands out as one of the most consumed^106–108^ and most studied supplements. However, only 13% of the research on the effects of caffeine has been undertaken specifically in women.^126^ In this systematic review, caffeine supplementation studies were the most numerous of the studies focused on female athletes or physically active women, with 10 articles being identified.^59–68^ All of these studies employed the posology considered to be ergogenic: a dosage of 3–6 mg/kg provided ∼60 minutes pre-exercise.^127^

Initially, the ergogenic effects of caffeine were associated with endurance performance and attributed to increased lipolysis and delayed depletion of muscle glycogen stores, with subsequent increase in TTE in endurance tests in combination with an increased blood fatty acids level.^128^ It is now understood that the ergogenic effects of caffeine are primarily associated with explosive and high intensity efforts, and secondarily associated with endurance performance. In the former, the effects of caffeine are exerted on the central nervous system, by blocking adenosine receptors A_1_ and A_2A_ and inhibiting parasympathetic system activity. This in turn, increases alertness and enhances mood, reduces the rate of perceived exertion (RPE), and increases cognitive performance.^129^ Additionally, at the peripheral level, caffeine increases the recruitment of motor unit fibers, the bioavailability of calcium in the myoplasm (increasing the release of calcium after an action potential), sodium–potassium pump activity, and intra- and inter-muscle coordination.^130^

The results of the studies included in this systematic review indicate that caffeine supplementation improves velocity and power during the execution of strength training,^61^^,^^64^^,^^65^ measured as maximum strength using 1-RM assessment,^65^ and muscular endurance, measured as the increment in the number of repetitions until failure with submaximal loads.^65^^,^^67^^,^^68^ Additionally, improvements were observed in sprint and/or agility tests,^66^ glycolytic performance (assessed in a Wingate test),^60^ and efforts characteristic of intermittent dynamic sports such as soccer.^61^ One potential concern about increasing physical performance is the possibility of inducing overtraining, due to an increase in the training load. However, a study included in this systematic review reported increased performance in torque production during knee extension, even at the end of an exercise session consisting of 6 blocks of 15 minutes each. This suggests that the enhancement of physical performance after caffeine supplementation does not necessarily lead to increased fatigue levels.^64^

Regarding the possible interaction of the MC with caffeine supplementation, 1 study reported ergogenic effects during both the early and late follicular phases and during the luteal phase.^60^ However, a second study reported ergogenic effects of caffeine on neuromuscular performance during the early and late follicular phases, but not during the luteal phase.^61^ The latter results suggest that there might be greater improvement in performance after supplementation during the follicular phase in female athletes than during the luteal phase. Additionally, a study carried out by Bougrine et al^66^ suggested that caffeine supplementation may have a greater effect on physical performance in the morning than in the evening. Finally, it should be borne in mind that caffeine affects sleep latency, ability to fall asleep, restlessness of sleep, and periods of wakefulness in female athletes.^63^ These side effects should be given consideration, as it has not been well established whether the possible enhancement of physical performance after supplementation could negatively interact with sleep.

Another modulator of nervous system activity with a chemical structure similar to that of caffeine is p-synephrine, which has been associated with an increase in fat oxidation during low to moderate intensity exercises.^69^ However, the only study that met the criteria of this review^69^ showed that an intake of 3 mg/kg of p-synephrine prior to an incremental exercise did not appear to increase fat oxidation or energy expenditure.

Beetroot juice supplementation is a dietary source rich in inorganic nitrate, which is a precursor of NO, through reduction processes that start in the mouth (from nitrate^–^ to nitrite) and continue in the stomach and at the systemic level (reducion of the nitrite to NO).^131^ NO is a potent vasodilator compound that enhances blood flow in skeletal muscles, improving muscle oxygenation, and contractile force in type II (ie, fast-twitch) muscle fibers. There is also recent evidence suggesting that NO has the capacity to increase intramuscular nitrate storage.^132^

Three studies identified in this systematic review assessed the effect of BRJ supplementation.^72–74^ In one of them, the ingestion of 70 ml of BRJ (containing 6.4 mmol of nitrate) failed to increase performance in a physical conditioning battery and a simulated hockey match in elite hockey players (ie, bronze team medallists in the Eurohockey Club Champions Cup).^74^ Conversely, Jurado-Castro et al^73^ reported an enhancement in CMJ, mean velocity, and power in back squat at 50% 1-RM, as well as in muscular endurance in a resistance training session composed of 3 exercises for lower limbs (back squat, leg press, and leg extension). Hemmatinafar et al^72^ sought to analyze the possible effect of BRJ on recovery after a training session that induced muscle damage (200 vertical jumps with weighted vests) in 12 semi-professional female volleyball players. They used a battery of functional tests (ie, V-Sit, reach flexibility test, vertical jump height, and wall-sit) and monitored muscle pain, always at 48 hours after the exercise-induced muscle damage (EIMD) protocol.^72^ Their findings suggested that BRJ supplementation in female volleyball players could be beneficial in improving certain recovery indicators, such as muscle soreness, flexibility, and explosive performance of lower limbs in the post-exercise phase. The studies on the precursors of NO focused on female athletes and physically active women indicated that this type of supplementation could increase physical performance and aid in post-exercise recovery.

Citrulline malate (CM) is another dietary supplement that could act ergogenically by increasing NO levels. Two studies identified in this systematic review assessed the effects of CM on physically active women^76^ and masters-aged female tennis players.^75^ Both studies indicated a beneficial effect in enhancing exercise performance in lower-body multiple-bout resistance exercise.^75^ Gills et al^76^ found an increase in total work done in 50 maximal repetitions of isokinetic knee extensions performed 60 minutes after ingesting 8 g of CM during the follicular phase of the MC in physically active women. The authors claimed that this supplement can be used as an ergogenic aid in resistance training, especially when NO production might be compromised, as is the case when estrogen levels are lower during the MC. Moreover, Glen et al^76^ reported an enhanced handgrip strength and increased explosive and peak power during a Wingate test in masters-aged tennis players after the consumption of 8 g of CM. The results of this systematic review suggest that supplementation with NO precursors might increase explosive and neuromuscular performance as well as post-exercise recovery. However, it is worth noting that the sample composition of these studies suggests that the beneficial effects of these supplements could be more pronounced in recreational or semi-professional athletes than in elite athletes.^133^

β-alanine and ${\text{NaHCO}}_{3}^{-}$ act as buffering agents.^134^^,^^135^ In the case of ${\text{NaHCO}}_{3}^{-}$, its effectiveness is attributed to the additional supply of both extracellular and intracellular buffering capacity, leading to increased concentrations in the blood and increased pH. In the case of β-alanine, its effectiveness is explained by its ability to increase muscle carnosine concentration, an ergogenic strategy in high-intensity exercise.^136^ Despite being among the few supplements with strong scientific support for their effectiveness, the consumption of these 2 supplements is relatively low among women^106–108^; only 23% of participants in the published studies supporting the use of sports supplements including β-alanine and ${\text{NaHCO}}_{3}^{-}$ are women.^15^ We note that the studies on the effectiveness of β-alanine have considerable heterogeneity. In an acute supplementation procedure, Glenn et al^88^ reported an increased TTE at 120% VO_2max_ after a single dose of 1.6 g of β-alanine. In long-term protocols, Smith et al^51^ reported an interaction between time and supplementation for the effect on body composition and RPE after 4 weeks of ingesting 4.8 g/day of β-alanine, but not for VO_2max_ or VT1. Thus, it is important to note that changes in VO_2max_ in trained athletes are limited and require at least an 8 week training period. The positive effect reported for RPE could reflect an enhanced training capacity in the participants, especially during interval training, due to a higher buffer capacity. In fact, this superior capacity for training could explain the enhancements observed in a consecutive 60 seconds of CMJs and a running-based anaerobic print test (RAST) in combination with plyometric training vs plyometric training alone. However, these effects have not been detected in other tests with lower demands in terms of glycolytic metabolism, as in 20-m sprints or in a single CMJ in women soccer players. In agreement with their results, Glenn et al^89^ reported mean power and total work done during the last third of a set of 50 maximal isokinetic leg extensions, an effort with significant glycolytic demands. The reduction in pH occurring as a consequence of increased glycolytic activity inhibits phosphofructokinase activity,^137^ hampering the glycolytic pathway and affecting muscle contraction by influencing competition between calcium and hydrogen ions at the troponin-binding site.^138^ β-alanine increases carnosine levels, facilitating the transport of calcium from the sarcoplasmic reticulum to the myoplasm, promoting more cross-bridging and potentially faster muscle contraction. Carnosine also acts as a buffer, capturing hydrogen ions and transporting them to the extracellular space.^139^ Given that women present lower levels of muscular carnosine compared with men^140^ and, considering the results of the studies included in this systematic review, it is plausible to state that female athletes and physically active women are likely to benefit from the ergogenic effects of β-alanine.

Regarding ${\text{NaHCO}}_{3}^{-}$, only 1 article met the inclusion criteria.^77^ That study reported a positive effect of ${\text{NaHCO}}_{3}^{-}$ in regulating blood pH, but did not find a significant improvement in mean sprint times in a 59-minute simulated match in an elite water polo squad. Future studies should investigate the effect of ${\text{NaHCO}}_{3}^{-}$ supplementation in female athletes, since the ingestion of ${\text{NaHCO}}_{3}^{-}$ increases blood pH, facilitating the buffering of excess hydrogen ions in the blood, delaying accumulation of hydrogen ions within muscle fibers and leading to increased glycolytic metabolism during efforts. In addition, these future studies should adhere to the recommended dose (200–400 mg/kg) in combination with a small CHO-rich meal (1.5 g/kg) 2–2.5 hours before exercise to minimize gastrointestinal symptoms.^87^

Creatine is considered one of the most effective supplements in the sports world^141^; however, its effectiveness in women has been largely overlooked until now. In this review, 4 studies conducted with creatine supplementation met the inclusion criteria. In elite female soccer players, supplementation with 20 g/day of creatine for 6 days increased body mass; however, overall, it did not improve performance in agility tasks.^70^ In a similar study, the effects of a 6-week plyometric training and creatine supplementation intervention on maximal-intensity and endurance performance in women soccer players showed that both placebo and creatine plyometric training groups showed improved performance in jumps, sprints, repeated sprinting, endurance, and change-of-direction speed, and that creatine did not impact on these parameters.^48^ The study of Brooks et al^71^ investigated the effects of 0.1 g/kg/day creatine supplementation for 6 weeks on body composition, performance, and cognitive function of female collegiate dancers. Creatine significantly impacted body composition by increasing total body water, lean mass (kilogram), and lower appendicular lean mass, but not the percentage of lean mass. No effects were detected in performance or cognitive function.^71^ Only 1 study considered the phase of the MC in relation to the effect of creatine supplementation in women. The study investigated the effects of 20 g/day of creatine supplementation for 5 days on performance and hormone levels in recreationally active women. During the luteal phase, sprint performance and recovery were significantly reduced in both groups, indicating that perhaps this phase of the MC would be the most appropriate for supplementation. However, creatine supplementation had no effect on heart rate variability (HRV), fatigue index, sprint performance, or recovery.

Creatine is a naturally occurring compound in the body, being stored primarily in the muscles and used for quick bursts of energy during high-intensity activities. Creatine supplementation has been shown to increase the body’s creatine phosphate stores, leading to enhanced performance in short-duration, high-intensity activities like sprints and jumps.^142^ The effects of creatine supplementation in women are multifaceted and influenced by various factors, including the type of physical activity, the training regimen, and hormonal fluctuations. The need for more comprehensive research that considers these variables and has higher sample sizes is evident if we are to better understand the nuanced relationship between creatine and athletic performance in females.

A fourth category of dietary supplements includes those that may indirectly improve physical performance by supporting the athlete’s health status, promoting a more suitable body composition, or enhancing the ability to train hard, recover fast, or avoid sport injuries.^91^ Within this category, bovine colostrum stands out as the most-studied dietary supplement for women, with a total of 4 studies.^80–83^ Bovine colostrum is a type of milk produced by cows during the first few days after giving birth. It contains a high concentration of mitogenic hormones and growth factors, which have been shown to increase the synthesis of skeletal muscle protein.^143^ Two studies investigated the effect of bovine colostrum on buffer capacity in female rowers, with different results.^82^^,^^83^ In the first study, supplementation with 40 g/day of protein from bovine colostrum for 9 weeks increased buffer capacity,^82^ whereas in a second study^83^ it did not. Neither study detected any effect on sporting performance. The discrepancy in these results may be due to the small sample size (less than 10 women in each study) and/or to the lack of detailed characterization of the colostrum composition. Two other studies investigated long-term (6 months) supplementation with 3.2 g/day of bovine colostrum on the immune system, iron homeostasis, oxidative stress, and inflammation,^80^^,^^81^ in women basketball players. Of all the parameters analyzed in relation to the immune system (IL-1-alpha, IL-2, IL-10, IL-13, TNF, IgG), supplementation only showed an effect on IL-10 levels (an increase compared with the control group), indicating a potential anti-inflammatory effect. Bovine colostrum supplementation was able to decrease muscle damage, as lower post-exercise CKMM levels were detected in the colostrum-supplemented group.^81^ The findings in relation to oxidative stress, inflammation, and iron homeostasis are also rather limited. Changes were only detected in the lipid peroxidation marker (TBARS) and in IL-6 levels, which were lower post-exercise in the supplemented group.^80^ Higher transferrin and lactoferrin values were found in the post-exercise stage after supplementation, which indicates that bovine colostrum may have an effect on iron homeostasis, which is of great importance for the female athlete.

The results of the studies included in this systematic review suggest that supplementation with bovine colostrum could reduce oxidative stress and inflammation through the presence of antioxidants, such as lactoferrin and caseins, which protect against free radical damage. In addition, bovine colostrum can modulate the inflammatory response and iron levels in the body, which may contribute to its beneficial effects.

Some studies included in the systematic review have evaluated the effects of various interventions based on the consumption of certain foods or supplements containing nutrients, bioactive compounds, or probiotics in female athletes.^55^^,^^84^ For example, the consumption of foods such as *Opuntia ficus indica* juice, which is rich in minerals, vitamins, proline, glutamine, taurine, and betalains, was shown to be associated with a statistically significant reduction in exercise-induced oxidative stress parameters, contributing to an accelerated recovery time.^84^ Furthermore, in a study conducted by Brown et al,^55^ supplementation with Montmorency tart cherry concentrate, which is rich in flavonoids and anthocyanins, was shown to be associated with an improvement in recovery after CMJ performance compared with the control group. Moreover, supplementation with avocado pulp (high in phenolic acids, flavonoids, and oleic acid) prior to exercise was associated with improved recovery after exercise in metrics of heart rate, systolic blood pressure, HRV, and skin conductance.^79^ Conversely, the study performed by Livolsi et al,^56^ which evaluated the effect of supplementation with chromium picolinate on muscular strength and body composition, found no significant benefits. On the other hand, probiotics purportedly show promise of beneficial effects in terms of recovery post-exercise, decreased muscle soreness, and decreased levels of markers of muscle damage.^53^ Toohey et al^53^ investigated the effect of *Bacillus subtilis* supplementation and found that probiotic consumption in combination with post-workout nutrition had no effect on either physical performance or hypertrophy; however, it may improve body composition by reducing body fat percentage. Supplementation with fish oil (6 g/day) for 3 weeks has not been shown to increase physical performance in 5 maximum isokinetic extensions, but it positively influenced parameters related to muscle damage and inflammation, such as myoglobin and TNF-α.^54^ The only study that assessed the effect of curcumin supplementation reported a positive influence of 500 mg/day of *Curcuma longa* L supplementation on inflammatory parameters and biomarkers of muscle damage, and an improvement in VO_2max_.^85^ Currently, all of these supplements are categorized as being supported by a low level of scientific evidence.^87^^,^^91^^,^^92^ The limited number of studies on them published to date suggests there is a need to conduct studies in female athletes and other physically active women on possible beneficial effects of the supplements categorized in this group. In addition, future studies should take into account the potential variation in responses to supplements among women, considering factors such as age, fitness level, and individual differences, which may significantly influence outcomes.

This is the first systematic review that has analyzed the findings regarding the effects of dietary manipulations and dietary supplementation on performance, recovery, and health status parameters in female athletes and other physically active women. The review has some limitations, related to the number and heterogeneity of the studies and their overall assessment as having “unclear” risk of bias. The first limitation, the limited number of studies analyzing the effects of dietary strategies on performance and health status in female athletes and other physically active women, is amplified by considerable heterogeneity in the types of interventions in the studies, the very limited information provided about control for menstrual function in adult athletes in the studies, and the minimal amount of research that has been conducted on menopausal and oligomenorrheic/amenorrheic athletes. The most important limitation is related to the detection of only 4 studies (5% of the studies included in the review) exhibited a low risk of bias. Further research in the female population is vital, including longitudinal studies to evaluate the long-term effects of dietary strategies on the performance and health of female athletes and other physically active women.

## CONCLUSIONS

The number of studies analyzing the effects of dietary strategies on performance and health status in female athletes and other physically active women is very limited. This is concomitant with large heterogeneity in the types of interventions of the available studies, very limited information about the influence of the menstrual function, and minimal research on populations including menopausal and oligomenorrheic/amenorrheic athletes. In addition, research on dietary supplements and their effectiveness has been more prevalent than research on dietary interventions in female athletes. Further research is clearly needed on dietary strategies for female physically active women and female athletes. Furthermore, longitudinal studies evaluating the long-term effects of dietary strategies on the performance and health of female athletes are essential. Regarding dietary interventions, we can conclude that HCHO diets influence energy metabolism, potentiating CHO metabolism during exercise and favoring recovery. However, HCHO diets only enhance physical performance in efforts that deplete muscle glycogen. In athletes with a HCHO diet, higher consumption of CHO in pre-exercise meals and dietary sources with a HGI increase CHO metabolism but have not been shown to improve physical performance. A calcium-rich dairy-based meal may have a positive effect on BMD without increasing gut discomfort. Dietary intake approaching the upper limit of the general recommended protein intake for athletes (1.2–2.0 g/kg/day), distributed over 5 to 6 meals throughout the day, with each meal containing at least 25 g of protein of high quality, may be optimal for ensuring training adaptations. At the same time, ensuring the PUFA-n3 content of the diet is above 500 g weekly of fish or seafood is recommended, and maintainance of an n-6/n-3 ratio below 2 should be considered.

Dietary supplements should be considered within an integral dietary plan for female athletes. It is crucial to review possible nutritional deficiencies, focusing on the most prevalent deficiencies (iron, calcium, vitamin C, folic acid, and vitamin B12). Iron status should be frequently reviewed, because iron supplementation may enhance performance even in the case of ID without anemia status. Under normal conditions, there is no evidence for a beneficial effect from intake amounts higher than the RDA. Sports foods can be beneficial for female athletes, especially in situations where there is limited access to foods. CHO supplements are effective for recovery after exercise involving consecutive efforts, and their ingestion with protein could have an added benefit compared with the intake of CHO alone. Whey protein at doses of 0.25–0.3 g/kg immediately post-exercise has been associated with improvement in physical performance and body composition (increasing lean body mass and reducing body fat percentage) in response to a resistance training program. The current data suggests that the use of supplements such as caffeine, precursors of NO, and β-alanine are associated with ergogenic effects in physically active females, specifically in non-elite athletes. The use of other supplements, such as creatine, sodium bicarbonate, α-lactalbumin, fish oil, bovine colostrum, probiotics (DE111), curcumin, and avocado pulp, require more extensive studies, because the existing data, while suggesting a possible effect, are not sufficient to justify recommending their use in female athletes.

This review underscores the importance of promoting educational initiatives aimed at raising awareness among female athletes and other physically active women about the importance of nutritional intake and its impact on performance and health. It is imperative that further gender-specific research is conducted to bridge the existing knowledge gaps regarding how dietary manipulation and supplements may impact men and women differently.

## Supplementary Material

nuae082_Supplementary_Data

## Data Availability

The datasets used and/or analyzed during the current study are available from the corresponding author on reasonable request.
