# Supplementary material for: Nutritional Strategies for Optimizing Health, Sports Performance, and Recovery for Female Athletes and Other Physically Active Women: A Systematic Review
Source: Nutr Rev. 2024 Jul 12;83(3):e1068–89. doi: 10.1093/nutrit/nuae082 (PMC11819490; doi:10.1093/nutrit/nuae082)
Supplement: nuae082_Supplementary_Data [file nuae082_supplementary_data.zip › nuae082_Supplementary_Data/Supplementary reference list complete.docx]

1. Walker JL, Heigenhauser GJ, Hultman E, Spriet LL. Dietary carbohydrate, muscle glycogen content, and endurance performance in well-trained women. *J Appl Physiol (1985)*. 2000;88(6):2151-2158. doi:10.1152/jappl.2000.88.6.2151
2. Reznik Dolins K, Boozer CN, Stoler F, Bartels M, DeMeersmane R, Contento I. Effect of variable carbohydrate intake on exercise performance in female endurance cyclists*. Int J Sport Nutr Exerc Metab.* 2003;13(4):422-435. doi:10.1123/ijsnem.13.4.422
3. Martínez-Rodríguez A, Rubio-Arias JA, García-De Frutos JM, Vicente-Martínez M, Gunnarsson TP. Effect of High-Intensity Interval Training and Intermittent Fasting on Body Composition and Physical Performance in Active Women. *Int J Environ Res Public Health*. 2021;18(12):6431. Published 2021 Jun 14. doi:10.3390/ijerph18126431
4. Vargas-Molina S, Gómez-Urquiza JL, García-Romero J, Benítez-Porres J. Effects of the Ketogenic Diet on Muscle Hypertrophy in Resistance-Trained Men and Women: A Systematic Review and Meta-Analysis. *Int J Environ Res Public Health*. 2022;19(19):12629. Published 2022 Oct 3. doi:10.3390/ijerph191912629
5. McLay RT, Thomson CD, Williams SM, Rehrer NJ. Carbohydrate loading and female endurance athletes: effect of menstrual-cycle phase. *Int J Sport Nutr Exerc Metab*. 2007;17(2):189-205. doi:10.1123/ijsnem.17.2.189
6. Stevenson EJ, Williams C, Mash LE, Phillips B, Nute ML. Influence of high-carbohydrate mixed meals with different glycemic indexes on substrate utilization during subsequent exercise in women. *Am J Clin Nutr*. 2006;84(2):354-360. doi:10.1093/ajcn/84.1.354
7. Stevenson E, Williams C, Nute M, Humphrey L, Witard O. Influence of the glycaemic index of an evening meal on substrate oxidation following breakfast and during exercise the next day in healthy women. *Eur J Clin Nutr*. 2008;62(5):608-616. doi:10.1038/sj.ejcn.1602759
8. Wynne JL, Ehlert AM, Wilson PB. Effects of high-carbohydrate versus mixed-macronutrient meals on female soccer physiology and performance. *Eur J Appl Physiol*. 2021;121(4):1125-1134. doi:10.1007/s00421-021-04597-5
9. Zeng Z, Jendricke P, Centner C, Storck H, Gollhofer A, König D. Acute Effects of Oatmeal on Exercise-Induced Reactive Oxygen Species Production Following High-Intensity Interval Training in Women: A Randomized Controlled Trial. *Antioxidants (Basel)*. 2020;10(1):3. Published 2020 Dec 22. doi:10.3390/antiox10010003
10. Haakonssen EC, Ross ML, Knight EJ, et al. The effects of a calcium-rich pre-exercise meal on biomarkers of calcium homeostasis in competitive female cyclists: a randomised crossover trial. *PLoS One*. 2015;10(5):e0123302. Published 2015 May 13. doi:10.1371/journal.pone.0123302
11. Haakonssen EC, Ross ML, Cato LE, et al. Dairy-based preexercise meal does not affect gut comfort or time-trial performance in female cyclists. *Int J Sport Nutr Exerc Metab*. 2014;24(5):553-558. doi:10.1123/ijsnem.2014-0069
12. Ormsbee MJ, Gorman KA, Miller EA, et al. Nighttime feeding likely alters morning metabolism but not exercise performance in female athletes. *Appl Physiol Nutr Metab*. 2016;41(7):719-727. doi:10.1139/apnm-2015-0526
13. Antonio J, Ellerbroek A, Evans C, Silver T, Peacock CA. High protein consumption in trained women: bad to the bone?. *J Int Soc Sports Nutr*. 2018;15:6. Published 2018 Jan 31. doi:10.1186/s12970-018-0210-6
14. Campbell BI, Aguilar D, Conlin L, et al. Effects of High Versus Low Protein Intake on Body Composition and Maximal Strength in Aspiring Female Physique Athletes Engaging in an 8-Week Resistance Training Program [published correction appears in Int J Sport Nutr Exerc Metab. 2020 Sep 1;30(5):383]. *Int J Sport Nutr Exerc Metab*. 2018;28(6):580-585. doi:10.1123/ijsnem.2017-0389
15. Arciero PJ, Ives SJ, Norton C, et al. Protein-Pacing and Multi-Component Exercise Training Improves Physical Performance Outcomes in Exercise-Trained Women: The PRISE 3 Study. *Nutrients*. 2016;8(6):332. Published 2016 Jun 1. doi:10.3390/nu8060332
16. Strandberg E, Ponsot E, Piehl-Aulin K, Falk G, Kadi F. Resistance Training Alone or Combined With N-3 PUFA-Rich Diet in Older Women: Effects on Muscle Fiber Hypertrophy. *J Gerontol A Biol Sci Med Sci*. 2019;74(4):489-494. doi:10.1093/gerona/gly130
17. De Souza MJ, Ricker EA, Mallinson RJ, et al. Bone mineral density in response to increased energy intake in exercising women with oligomenorrhea/amenorrhea: the REFUEL randomized controlled trial. *Am J Clin Nutr*. 2022;115(6):1457-1472. doi:10.1093/ajcn/nqac044
18. Miralles-Amorós L, Vicente-Martínez M, Martínez-Olcina M, et al. Study of Different Personalised Dietary Plans on Eating Behaviour, Body Image and Mood in Young Female Professional Handball Players: A Randomised Controlled Trial. *Children (Basel)*. 2023;10(2):259. Published 2023 Jan 31. doi:10.3390/children10020259
19. DellaValle DM, Haas JD. Iron supplementation improves energetic efficiency in iron-depleted female rowers. *Med Sci Sports Exerc*. 2014;46(6):1204-1215. doi:10.1249/MSS.0000000000000208
20. Sandroni A, House E, Howard L, DellaValle DM. Synbiotic Supplementation Improves Response to Iron Supplementation in Female Athletes during Training. *J Diet Suppl*. 2022;19(3):366-380. doi:10.1080/19390211.2021.1887423
21. McClung JP, Karl JP, Cable SJ, et al. Randomized, double-blind, placebo-controlled trial of iron supplementation in female soldiers during military training: effects on iron status, physical performance, and mood. *Am J Clin Nutr*. 2009;90(1):124-131. doi:10.3945/ajcn.2009.27774
22. Hoch AZ, Pajewski NM, Hoffmann RG, Schimke JE, Gutterman DD. Possible relationship of folic Acid supplementation and improved flow-mediated dilation in premenopausal, eumenorrheic athletic women. *J Sports Sci Med*. 2009;8(1):123-129.
23. Taghiyar M, Darvishi L, Askari G, et al. The effect of vitamin C and e supplementation on muscle damage and oxidative stress in female athletes: a clinical trial. *Int J Prev Med*. 2013;4(Suppl 1):16-23.
24. Taghiyar M, Ghiasvand R, Askari G, et al. The effect of vitamins C and e supplementation on muscle damage, performance, and body composition in athlete women: a clinical trial. *Int J Prev Med*. 2013;4(Suppl 1):24-30.
25. Mock MG, Hirsch KR, Blue MNM, Trexler ET, Roelofs EJ, Smith-Ryan AE. Post-Exercise Ingestion of Low or High Molecular Weight Glucose Polymer Solution Does Not Improve Cycle Performance in Female Athletes. *J Strength Cond Res*. 2021;35(1):124-131. doi:10.1519/JSC.0000000000002560
26. McCleave EL, Ferguson-Stegall L, Ding Z, et al. A low carbohydrate-protein supplement improves endurance performance in female athletes. *J Strength Cond Res*. 2011;25(4):879-888. doi:10.1519/JSC.0b013e318207e98c
27. Hida A, Hasegawa Y, Mekata Y, et al. Effects of egg white protein supplementation on muscle strength and serum free amino acid concentrations. *Nutrients*. 2012;4(10):1504-1517. Published 2012 Oct 19. doi:10.3390/nu4101504
28. Taylor LW, Wilborn C, Roberts MD, White A, Dugan K. Eight weeks of pre- and postexercise whey protein supplementation increases lean body mass and improves performance in Division III collegiate female basketball players. *Appl Physiol Nutr Metab*. 2016;41(3):249-254. doi:10.1139/apnm-2015-0463
29. Wilborn CD, Outlaw JJ, Mumford PW, et al. A Pilot Study Examining the Effects of 8-Week Whey Protein versus Whey Protein Plus Creatine Supplementation on Body Composition and Performance Variables in Resistance-Trained Women. *Ann Nutr Metab*. 2016;69(3-4):190-199. doi:10.1159/000452845
30. Gratwicke M, Miles KH, Clark B, Pumpa KL. The effect of α-lactalbumin consumption on sleep quality and quantity in female rugby union athletes: a field-based study. *Biol Sport*. 2023;40(2):449-455. doi:10.5114/biolsport.2023.116002
31. Miles KH, Clark B, Fowler PM, et al. ɑ-Lactalbumin Improves Sleep and Recovery after Simulated Evening Competition in Female Athletes. *Med Sci Sports Exerc*. 2021;53(12):2618-2627. doi:10.1249/MSS.0000000000002743
32. Lara B, Gonzalez-Millán C, Salinero JJ, et al. Caffeine-containing energy drink improves physical performance in female soccer players. *Amino Acids*. 2014;46(5):1385-1392. doi:10.1007/s00726-014-1709-z
33. Lara B, Gutiérrez Hellín J, Ruíz-Moreno C, Romero-Moraleda B, Del Coso J. Acute caffeine intake increases performance in the 15-s Wingate test during the menstrual cycle. *Br J Clin Pharmacol*. 2020;86(4):745-752. doi:10.1111/bcp.14175
34. Romero-Moraleda B, Del Coso J, Gutiérrez-Hellín J, Lara B. The Effect of Caffeine on the Velocity of Half-Squat Exercise during the Menstrual Cycle: A Randomized Controlled Trial. *Nutrients*. 2019;11(11):2662. Published 2019 Nov 4. doi:10.3390/nu11112662
35. Filip-Stachnik A, Krzysztofik M, Kaszuba M, et al. Placebo Effect of Caffeine on Maximal Strength and Strength Endurance in Healthy Recreationally Trained Women Habituated to Caffeine. *Nutrients*. 2020;12(12):3813. Published 2020 Dec 13. doi:10.3390/nu12123813
36. Ali A, O'Donnell JM, Starck C, Rutherfurd-Markwick KJ. The Effect of Caffeine Ingestion during Evening Exercise on Subsequent Sleep Quality in Females. *Int J Sports Med*. 2015;36(6):433-439. doi:10.1055/s-0034-1398580
37. Ali A, O'Donnell J, Foskett A, Rutherfurd-Markwick K. The influence of caffeine ingestion on strength and power performance in female team-sport players. *J Int Soc Sports Nutr*. 2016;13:46. Published 2016 Dec 5. doi:10.1186/s12970-016-0157-4
38. Norum M, Risvang LC, Bjørnsen T, et al. Caffeine increases strength and power performance in resistance-trained females during early follicular phase. *Scand J Med Sci Sports*. 2020;30(11):2116-2129. doi:10.1111/sms.13776
39. Bougrine H, Nasser N, Abdessalem R, Ammar A, Chtourou H, Souissi N. Pre-Exercise Caffeine Intake Attenuates the Negative Effects of Ramadan Fasting on Several Aspects of High-Intensity Short-Term Maximal Performances in Adolescent Female Handball Players. *Nutrients*. 2023;15(15):3432. Published 2023 Aug 3. doi:10.3390/nu15153432
40. Karayigit R, Naderi A, Akca F, et al. Effects of Different Doses of Caffeinated Coffee on Muscular Endurance, Cognitive Performance, and Cardiac Autonomic Modulation in Caffeine Naive Female Athletes. *Nutrients*. 2020;13(1):2. Published 2020 Dec 22. doi:10.3390/nu13010002
41. Pereira PEA, Azevedo P, Azevedo K, Azevedo W, Machado M. Caffeine Supplementation or Carbohydrate Mouth Rinse Improves Performance. *Int J Sports Med*. 2021;42(2):147-152. doi:10.1055/a-1212-0742
42. Gutiérrez-Hellín J, Aguilar-Navarro M, Ruiz-Moreno C, et al. Effect of p-Synephrine on Fat Oxidation Rate during Exercise of Increasing Intensity in Healthy Active Women. *Nutrients*. 2022;14(20):4352. Published 2022 Oct 17. doi:10.3390/nu14204352
43. Cox G, Mujika I, Tumilty D, Burke L. Acute creatine supplementation and performance during a field test simulating match play in elite female soccer players. *Int J Sport Nutr Exerc Metab*. 2002;12(1):33-46. doi:10.1123/ijsnem.12.1.33
44. Ramírez-Campillo R, González-Jurado JA, Martínez C, et al. Effects of plyometric training and creatine supplementation on maximal-intensity exercise and endurance in female soccer players. *J Sci Med Sport*. 2016;19(8):682-687. doi:10.1016/j.jsams.2015.10.005
45. Gordon AN, Moore SR, Patterson ND, et al. The Effects of Creatine Monohydrate Loading on Exercise Recovery in Active Women throughout the Menstrual Cycle. *Nutrients*. 2023;15(16):3567. Published 2023 Aug 13. doi:10.3390/nu15163567
46. Brooks SJ, Candow DG, Roe AJ, et al. Creatine monohydrate supplementation changes total body water and DXA lean mass estimates in female collegiate dancers. *J Int Soc Sports Nutr*. 2023;20(1):2193556. doi:10.1080/15502783.2023.2193556
47. Hemmatinafar M, Zaremoayedi L, Koushkie Jahromi M, et al. Effect of Beetroot Juice Supplementation on Muscle Soreness and Performance Recovery after Exercise-Induced Muscle Damage in Female Volleyball Players. *Nutrients*. 2023;15(17):3763. Published 2023 Aug 28. doi:10.3390/nu15173763
48. Jurado-Castro JM, Campos-Perez J, Ranchal-Sanchez A, Durán-López N, Domínguez R. Acute Effects of Beetroot Juice Supplements on Lower-Body Strength in Female Athletes: Double-Blind Crossover Randomized Trial. *Sports Health*. 2022;14(6):812-821. doi:10.1177/19417381221083590
49. López-Samanes Á, Pérez-Lopez A, Morencos E, et al. Beetroot juice ingestion does not improve neuromuscular performance and match-play demands in elite female hockey players: a randomized, double-blind, placebo-controlled study. *Eur J Nutr*. 2023;62(3):1123-1130. doi:10.1007/s00394-022-03052-1
50. Glenn JM, Gray M, Jensen A, Stone MS, Vincenzo JL. Acute citrulline-malate supplementation improves maximal strength and anaerobic power in female, masters athletes tennis players. *Eur J Sport Sci*. 2016;16(8):1095-1103. doi:10.1080/17461391.2016.1158321
51. Gills JL, Spliker B, Glenn JM, et al. Acute Citrulline-Malate Supplementation Increases Total Work in Short Lower-Body Isokinetic Tasks for Recreationally Active Females During Menstruation. *J Strength Cond Res*. 2023;37(6):1225-1230. doi:10.1519/JSC.0000000000004095
52. Glenn JM, Gray M, Stewart R, et al. Incremental effects of 28 days of beta-alanine supplementation on high-intensity cycling performance and blood lactate in masters female cyclists. *Amino Acids*. 2015;47(12):2593-2600. doi:10.1007/s00726-015-2050-x
53. Smith AE, Stout JR, Kendall KL, Fukuda DH, Cramer JT. Exercise-induced oxidative stress: the effects of β-alanine supplementation in women. *Amino Acids*. 2012;43(1):77-90. doi:10.1007/s00726-011-1158-x
54. Glenn JM, Smith K, Moyen NE, Binns A, Gray M. Effects of Acute Beta-Alanine Supplementation on Anaerobic Performance in Trained Female Cyclists. *J Nutr Sci Vitaminol (Tokyo)*. 2015;61(2):161-166. doi:10.3177/jnsv.61.161
55. Rosas F, Ramírez-Campillo R, Martínez C, et al. Effects of Plyometric Training and Beta-Alanine Supplementation on Maximal-Intensity Exercise and Endurance in Female Soccer Players. *J Hum Kinet.* 2017;58:99-109. Published 2017 Aug 1. doi:10.1515/hukin-2017-0072
56. Glenn JM, Gray M, Stewart RW Jr, et al. Effects of 28-Day Beta-Alanine Supplementation on Isokinetic Exercise Performance and Body Composition in Female Masters Athletes. *J Strength Cond Res.* 2016;30(1):200-207.
57. Tan F, Polglaze T, Cox G, Dawson B, Mujika I, Clark S. Effects of induced alkalosis on simulated match performance in elite female water polo players. *Int J Sport Nutr Exerc Metab.* 2010;20(3):198-205. doi:10.1123/ijsnem.20.3.198
58. Köhne JL, Ormsbee MJ, McKune AJ. The effects of a multi-ingredient supplement on markers of muscle damage and inflammation following downhill running in females. *J Int Soc Sports Nutr.* 2016;13:44. Published 2016 Nov 25. doi:10.1186/s12970-016-0156-5
59. Cameron M, Camic CL, Doberstein S, Erickson JL, Jagim AR. The acute effects of a multi-ingredient pre-workout supplement on resting energy expenditure and exercise performance in recreationally active females. *J Int Soc Sports Nutr.* 2018;15:1. Published 2018 Jan 5. doi:10.1186/s12970-017-0206-7
60. Cieślicka M, Ostapiuk-Karolczuk J, Buttar HS, Dziewiecka H, Kasperska A, Skarpańska-Stejnborn A, Cieślicka M, Dziewiecka H, et al. Effects of Long-Term Supplementation of Bovine Colostrum on the Immune System in Young Female Basketball Players. Randomized Trial. *Nutrients.* 2020;13(1):118. Published 2020 Dec 30. doi:10.3390/nu13010118
61. Skarpańska-Stejnborn A. Effects of Long-Term Supplementation of Bovine Colostrum on Iron Homeostasis, Oxidative Stress, and Inflammation in Female Athletes: A Placebo-Controlled Clinical Trial. *Nutrients*. 2022;15(1):186. Published 2022 Dec 30. doi:10.3390/nu15010186
62. Brinkworth GD, Buckley JD, Bourdon PC, Gulbin JP, David A. Oral bovine colostrum supplementation enhances buffer capacity but not rowing performance in elite female rowers. *Int J Sport Nutr Exerc Metab*. 2002;12(3):349-365. doi:10.1123/ijsnem.12.3.349
63. Brinkworth GD, Buckley JD. Bovine colostrum supplementation does not affect plasma buffer capacity or haemoglobin content in elite female rowers. *Eur J Appl Physiol*. 2004;91(2-3):353-356. doi:10.1007/s00421-003-1023-z
64. Toohey JC, Townsend JR, Johnson SB, et al. Effects of Probiotic (Bacillus subtilis) Supplementation During Offseason Resistance Training in Female Division I Athletes. *J Strength Cond Res*. 2020;34(11):3173-3181. doi:10.1519/JSC.0000000000002675
65. McKinley-Barnard SK, Andre TL, Gann JJ, Hwang PS, Willoughby DS. Effectiveness of Fish Oil Supplementation in Attenuating Exercise-Induced Muscle Damage in Women During Midfollicular and Midluteal Menstrual Phases. *J Strength Cond Res*. 2018;32(6):1601-1612. doi:10.1519/JSC.0000000000002247
66. Sousa FH, Valenti VE, Pereira LC, et al. Avocado (Persea americana) pulp improves cardiovascular and autonomic recovery following submaximal running: a crossover, randomized, double-blind and placebo-controlled trial. *Sci Rep*. 2020;10(1):10703. Published 2020 Jul 1. doi:10.1038/s41598-020-67577-3
67. Bellafiore M, Pintaudi AM, Thomas E, et al. Redox and autonomic responses to acute exercise-post recovery following Opuntia ficus-indica juice intake in physically active women. *J Int Soc Sports Nutr*. 2021;18(1):43. Published 2021 Jun 7. doi:10.1186/s12970-021-00444-2
68. Salehi M, Mashhadi NS, Esfahani PS, Feizi A, Hadi A, Askari G. The Effects of Curcumin Supplementation on Muscle Damage, Oxidative Stress, and Inflammatory Markers in Healthy Females with Moderate Physical Activity: A Randomized, Double-Blind, Placebo-Controlled Clinical Trial. *Int J Prev Med*. 2021;12:94. Published 2021 Jul 29. doi:10.4103/ijpvm.IJPVM_138_20
69. Brown MA, Stevenson EJ, Howatson G. Montmorency tart cherry (Prunus cerasus L.) supplementation accelerates recovery from exercise-induced muscle damage in females. *Eur J Sport Sci*. 2019;19(1):95-102. doi:10.1080/17461391.2018.1502360
70. Livolsi JM, Adams GM, Laguna PL. The effect of chromium picolinate on muscular strength and body composition in women athletes. *J Strength Cond Res*. 2001;15(2):161-166.
71. Farjallah MA, Hammouda O, Zouch M, et al. Effect of melatonin ingestion on physical performance, metabolic responses, and recovery after an intermittent training session. *Physiol Int*. 2018;105(4):358-370. doi:10.1556/2060.105.2018.4.24
