# Supplementary material for: Nutritional Strategies for Optimizing Health, Sports Performance, and Recovery for Female Athletes and Other Physically Active Women: A Systematic Review
Source: Nutr Rev. 2024 Jul 12;83(3):e1068–89. doi: 10.1093/nutrit/nuae082 (PMC11819490; doi:10.1093/nutrit/nuae082)
Supplement: nuae082_Supplementary_Data [file nuae082_supplementary_data.zip › nuae082_Supplementary_Data/SupplementaryMaterialS2.docx]

**Supplementary Material S2**

In this supplementary document is specified the exactly search strategy used In the different databases used in this systematic review: Pubmed, Scopus and Web of Science.

***Search strategy used in Pubmed:***

(("Women"[Title/Abstract] OR "female"[Title/Abstract]) AND ("nutri*"[Title/Abstract] OR "Micronutrients"[Title/Abstract] OR "Macronutrients"[Title/Abstract] OR "aminoacids"[Title/Abstract] OR "ergogenic aid"[Title/Abstract] OR "CHO"[Title/Abstract] OR "Carbohydrates"[Title/Abstract] OR "glucose"[Title/Abstract] OR "Lipids"[Title/Abstract] OR "fats proteins"[Title/Abstract] OR "Minerals"[Title/Abstract] OR "fiber"[Title/Abstract] OR "Vitamins"[Title/Abstract] OR "diet*"[Title/Abstract] OR "Probiotics"[Title/Abstract] OR "Prebiotics"[Title/Abstract] OR "symbiotics*"[Title/Abstract] OR "nutraceutic*"[Title/Abstract] OR "functional foods"[Title/Abstract] OR "supplement*"[Title/Abstract] OR "Non-nutrient"[Title/Abstract] OR "phytochemical"[Title/Abstract]) AND ("Sport"[Title/Abstract] OR "exercise"[Title/Abstract] OR "athlet*"[Title/Abstract] OR "physically activ*"[Title/Abstract]) AND ("health status"[Title/Abstract] OR "healthy"[Title/Abstract] OR "sport performance"[Title/Abstract] OR "conditioning"[Title/Abstract] OR "muscle performance"[Title/Abstract] OR "endurance"[Title/Abstract] OR "strength"[Title/Abstract] OR "speed"[Title/Abstract] OR "agility"[Title/Abstract] OR "competition"[Title/Abstract] OR "biochemical"[Title/Abstract] OR "physiolog*"[Title/Abstract] OR "contest"[Title/Abstract] OR "DOMS"[Title/Abstract] OR "hormonal behaviour"[Title/Abstract] OR "nutritional status"[Title/Abstract] OR "bone health"[Title/Abstract] OR "hematolog*"[Title/Abstract] OR "oxidative stress"[Title/Abstract] OR ("inflammat*"[All Fields] AND "microbio*"[Title/Abstract]))) AND ((randomizedcontrolledtrial[Filter]) AND (humans[Filter]) AND (female[Filter]) AND (english[Filter] OR portuguese[Filter] OR spanish[Filter]) AND (2000:2023[pdat])

***Search strategy used in Scopus:***

(("Women"[Title/Abstract] OR "female"[Title/Abstract]) AND ("nutri*"[Title/Abstract] OR "Micronutrients"[Title/Abstract] OR "Macronutrients"[Title/Abstract] OR "aminoacids"[Title/Abstract] OR "ergogenic aid"[Title/Abstract] OR "CHO"[Title/Abstract] OR "Carbohydrates"[Title/Abstract] OR "glucose"[Title/Abstract] OR "Lipids"[Title/Abstract] OR "fats proteins"[Title/Abstract] OR "Minerals"[Title/Abstract] OR "fiber"[Title/Abstract] OR "Vitamins"[Title/Abstract] OR "diet*"[Title/Abstract] OR "Probiotics"[Title/Abstract] OR "Prebiotics"[Title/Abstract] OR "symbiotics*"[Title/Abstract] OR "nutraceutic*"[Title/Abstract] OR "functional foods"[Title/Abstract] OR "supplement*"[Title/Abstract] OR "Non-nutrient"[Title/Abstract] OR "phytochemical"[Title/Abstract]) AND ("Sport"[Title/Abstract] OR "exercise"[Title/Abstract] OR "athlet*"[Title/Abstract] OR "physically activ*"[Title/Abstract]) AND ("health status"[Title/Abstract] OR "healthy"[Title/Abstract] OR "sport performance"[Title/Abstract] OR "conditioning"[Title/Abstract] OR "muscle performance"[Title/Abstract] OR "endurance"[Title/Abstract] OR "strength"[Title/Abstract] OR "speed"[Title/Abstract] OR "agility"[Title/Abstract] OR "competition"[Title/Abstract] OR "biochemical"[Title/Abstract] OR "physiolog*"[Title/Abstract] OR "contest"[Title/Abstract] OR "DOMS"[Title/Abstract] OR "hormonal behaviour"[Title/Abstract] OR "nutritional status"[Title/Abstract] OR "bone health"[Title/Abstract] OR "hematolog*"[Title/Abstract] OR "oxidative stress"[Title/Abstract] OR ("inflammat*"[All Fields] AND "microbio*"[Title/Abstract]))) AND ((randomizedcontrolledtrial[Filter]) AND (humans[Filter]) AND (female[Filter]) AND (english[Filter] OR portuguese[Filter] OR spanish[Filter]) AND (2000:2023[pdat])

***Search strategy used in Web of Science:***

((((((((((((((((((((((((((((((((((((((((((((((((((((((((((((((((((((((((((((((((((((((((((((((((((((((((((((((TI=(women)) OR AB=(women)) OR TI=(female)) OR AB=(female)) AND TI=(nutri*)) OR AB=(nutri*)) OR TI=(micronutrients )) OR AB=(micronutrients )) OR TI=(macronutrients )) OR AB=(macronutrients)) OR TI=(aminoacids)) OR AB=(aminoacids)) OR TI=(“ergogenic aid”)) OR AB=(“ergogenic aid”)) OR TI=(CHO)) OR AB=(CHO)) OR TI=(carbohydrates)) OR AB=(carbohydrates)) OR TI=(glucose)) OR AB=(glucose)) OR TI=(lipids)) OR AB=(lipids)) OR TI=(fats)) OR AB=(fats)) OR TI=(proteins)) OR AB=(proteins)) OR TI=(Minerals)) OR AB=(Minerals)) OR TI=(fiber)) OR AB=(fiber)) OR TI=(fibre)) OR AB=(fibre)) OR TI=(vitamins)) OR AB=(vitamins)) OR TI=(diet*)) OR AB=(diet*)) OR TI=(probiotics)) OR AB=(probiotics)) OR TI=(prebiotics)) OR AB=(prebiotics)) OR TI=(symbiotics*)) OR AB=(symbiotics*)) OR TI=(nutraceutic*)) OR AB=(nutraceutic*)) OR TI=(“functional foods”)) OR AB=(“functional foods”)) OR TI=(supplement* )) OR AB=(supplement* )) OR TI=(“non-nutrient”)) OR AB=(“non-nutrient”)) OR TI=(phytochemical)) OR AB=(phytochemical)) AND TI=(sport)) OR AB=(sport)) OR TI=(exercise)) OR AB=(exercise)) OR TI=(athlet*)) OR AB=(athlet*)) OR TI=(“physical activ*”)) OR AB=(“physically activ*”)) AND TI=(“health status”)) OR AB=(“health status”)) OR TI=(healthy)) OR AB=(healthy)) OR TI=(“sport performance”)) OR AB=(“sport performance”)) OR TI=(conditioning)) OR AB=(conditioning)) OR TI=(“muscle performance”)) OR AB=(“muscle performance”)) OR TI=(endurance)) OR AB=(endurance)) OR TI=(strength)) OR AB=(strength)) OR TI=(speed)) OR AB=(speed)) OR TI=(agility)) OR AB=(agility)) OR TI=(“international concurrence”)) OR AB=(“international concurrence”)) OR TI=( competition)) OR AB=( competition)) OR TI=(biochemical)) OR AB=(biochemical)) OR TI=(physiolog*)) OR AB=(physiolog*)) OR TI=(contest)) OR AB=(contest)) OR TI=(“sport biomarker”)) OR AB=(“sport biomarker”)) OR TI=(DOMS)) OR AB=(DOMS)) OR TI=(“hormonal behaviour”)) OR AB=(“hormonal behaviour”)) OR TI=(“nutritional status”)) OR AB=(“nutritional status”)) OR TI=(“bone health”)) OR AB=(“bone health”)) OR TI=(hematolog*)) OR AB=(hematolog*)) OR TI=(“oxidative stress”)) OR AB=(“oxidative stress”)) OR TI=(inflammat*)) OR AB=(inflammat*)) OR TI=(microbio*)) OR AB=(microbio*)) AND ALL=("randomized controlled trial")) OR ALL=("randomised controlled trial")) NOT ALL=(mice)) NOT ALL=(rat*)) NOT ALL=("in vivo")
