# Supplementary material for: Nutritional Strategies for Optimizing Health, Sports Performance, and Recovery for Female Athletes and Other Physically Active Women: A Systematic Review
Source: Nutr Rev. 2024 Jul 12;83(3):e1068–89. doi: 10.1093/nutrit/nuae082 (PMC11819490; doi:10.1093/nutrit/nuae082)
Supplement: nuae082_Supplementary_Data [file nuae082_supplementary_data.zip › nuae082_Supplementary_Data/SupplementaryMaterialS3_new.docx]

**Supplementary Material S3***.* **Summarize of studies included in the systematic review focused on interventions based on manipulation of the diet**

| Reference | Population | Control of menstrual function | Dietary control | Intervention | | | Outcomes | Results |
| --- | --- | --- | --- | --- | --- | --- | --- | --- |
|  |  |  |  | Experimental group/conditions | Characteristics | Duration |  |  |
| Walker (2000) ^S1^ | 6 Highly endurance athletes (27.4±1.7 years) | Eumenorrheic. Trials were performed in the luteal phase | It existed a frequent communication between researchers and participants to ensure accurate adherence to the diet | EC1: High CHO diet (HCHO)  EC2: Control | EC1: 3 days of a mixed diet (48% daily energy from CHO) and high volume of training + 3 days of a high CHCO diet (78% daily energy) and low volume of training  EC2: 7 days of a mixed diet (48% daily energy from CHO) | 1 week (COD) | TTE cycling at 80-82% VO2max: VO_2_, RER, insulin, glucose, FFA, glycerol and BLA and rate of CHO and fat oxidation, individual pre- and post-exercise glycogen content and net glycogen utilization during exercise | Comparison EC1 vs EC2:  ↑TTE cycling at 80-82% VO_2max_,, RER, glucose, BLA rate of CHO oxidation during exercise, individual pre-and post-exercise glycogen content and net glycogen utilization during exercise  ↓RER, FFA, glycerol and rate of fat oxidation during exercise |
| Dolins (2003) ^S2^ | 11 Trained cyclists (32.5±6.4 years) | Eumenorrheic. Trials were performed in the mid luteal phase | Testing days, participants ingested a standardized breakfast (360 kcal) 3-4 h pre-exercise | EC1: High CHO diet (HCHO)  EC2: Moderate CHO diet (MCHO)  EC3: Low CHO diet (LCHO) | EC1: 8 g/kg/day CHO  EC2: 5 g/kg/day CHO  EC3: 3 g/kg/day CHO | 1 week (COD) | TTE at 90%: time, VO_2_, RER and RPE | Not differences were reported between ECs |
| Martínez-Rodríguez (2021) ^S3^ | 14 Physically active (27±6 years) | Eumenorrheic. Trials were performed in the same phase of the MC | Distribution of macronutrients for EGs was as follow: 5.8 g/kg/day of CHO - 1.8-2 g/kg of protein - 1-1.2 g/kg of lipids. Not additional dietary control was reported | EG1: HIIT + intermittent fasting (HIIT + IF)  EG2: HIIT with control diet (HIIT + CON) | EG1: Participants were instructed not to eat in <14 h of the day before and consume breakfast as soon as possible after waking and continuing to eat following the diet intervals.  EG2: Participants had to maintain their usual dietary intake based on a Mediterranean diet | 8 weeks (COD) | CMJ, handgrip strength, Wingate test (mean power and peak power), 10 skinfold, girths and body composition | ↑CMJ (EG1)  Interaction time·intervention: skinfold (triceps, biceps and leg) and fat mass (kg) |
| Vargas-Molina (2020) ^S4^ | 21 Strength trained athletes (EG1: n=10, 26.8±3.9 years; EG2: n=11, 28.3±4.1 years) | Eumenorrheic. Trials were performed in the follicular phase (7 days after menstruation) | Daily dietary intake was supervised using a smartphone app | EG1: Ketogenic diet  EG2: control | Distributed between 3-6 meals daily:  EG1: 40-45 kcal/kg BLM (0.9 g/kg/day CHO – 2.7 g/kg/day protein – 2.9 g/kg/day fat)  EG2: 40-45 kcal/kg BLM (6.5 g/kg/day CHO – 2.2 g/kg/day protein – 1.0 g/kg/day fat) | 4 weeks  (PGD) | Body mass, fat mass, BLM, CMJ and 1-RM in bench press and squat | ↑1-RM bench press (EG2) and squat (EG1 and EG2) and CMJ (EG1 and EG2)  ↓Body mass and fat mass (EG1)  Interaction time·intervention: Body mass, fat mass, BLM, CMJ and 1-RM in bench press and squat |
| McLay (2007) ^S5^ | 6 Moderately trained cyclists (25.1±7.3 years) | Eumenorrheic. Trials were performed in the mid follicular (days 8-12 of the MC) or mid luteal phase (days 21-29 of the MC) | Testing days, participants ingested a standardized breakfast (87 g CHO - 14 g protein - 2 g fat) 1.5–2 h before trials | EC1: High diet in mid follicular phase (MF/HCHO)  EC2: control diet in mid follicular phase (MF/CON)  EC3: High diet in mid luteal phase (ML/HCHO)  EC4: control diet in mid luteal phase (ML/CON) | EC1: 8.4 g/kg/day CHO in mid follicular phase  EC2: 5.2 g/kg/day CHO in mid follicular phase  EC3: 8.4 g/kg/day CHO in mid luteal phase  EC4: 5.2 g/kg/day CHO in mid luteal phase | 3 days (COD) | 70 min cycling between 45-75% VO_2max_ + 16-km TT: TT (time), BLA, haemoglobin, haematocrit, rate of CHO and fat oxidation, muscle glycogen content pre-exercise | ↑Rate of CHO oxidation (EC1 vs EC2 and EC3 vs EC4) and muscle glycogen content pre-exercise (EC1, EC3 and EC4 vs EC2)  ↓Rate of fat oxidation (EC1 vs EC2 and EC3 vs EC4) |
| Stevenson (2006) ^S6^ | 18 Physically active (18.6±0.9 years) | Eumenorrheic. It was not controlled MC phase in the study | Testing days, participants ingested a standardized breakfast during the postprandial period, only water was permitted | EC1: High glycaemic index CHO (HGI)  EC2: Low glycaemic index CHO (LGI) | Breakfast (3-h pre-exercise) composed by:  EC1: High glycaemic index (3054 kJ - 2 g/kg of CHO - 20 g of protein - 10 g of fat)  EC2: Low glycaemic index (3063 kJ - 2 g/kg of CHO - 23 g of protein - 9 g of fat) | Acute (COD) | At the rest and during exercise (60 min running at 65% VO_2max_): glucose, insulin, FFA, glycerol, rate of CHO and fat oxidation, RPE | Comparison EC1 vs EC2:  At rest:  ↑Glucose and insulin  ↓FFA  During exercise:  ↑Glucose and rate of CHO oxidation  ↓FFA, glycerol and rate of fat oxidation |
| Stevenson (2008) ^S7^ | 7 Physically active (24.4±3.4 years) | Eumenorrheic. Participants were randomised to each experimental condition based on the phase of the MC | Testing days, participants arrived after 13-h overnight fast. Before the first trial, 2-days dietary intake was recorded for replicating it prior to the second trial | EC1: meal with high glycaemic index CHO (HGI)  EC2: meal with low glycaemic index CHO (LGI) | Breakfast (3-h pre-exercise) composed by:  EC1: 2 g/kg/day of HGI  EC2: 2 g/kg/day of LGI | Acute (COD) | At the rest and during exercise (60 min running at 65% VO_2max_): VO_2_, RER, glucose insulin, FFA and plasma glycerol | Comparison EC1 vs EC2:  At rest:  ↑Glucose, insulin and FFA  During exercise: not differences were reported between ECs |
| Wynne (2021) ^S8^ | 21 National soccer players (19.6±1.3 years) | Not reported | To refrain from eating any energy-containing foods 3-h previousto the pre-scrimmage meal | EC1: CHO pre-scrimmage meal  EC2: control | EC1: 1000 kcal – 203 g CHO – 21 g protein - 21 g fat (4-h pre-exercise)  EC2: 1000 kcal – 103 g CHO – 52 g protein - 48.5 g fat | Acute (COD) | In a simulated match of 2 halves of 35 min in a reduced space: total distance covered, high speed running, sprint count, hearth rate, rate of fatigue and RPE | Not differences were reported between ECs |
| Zeng (2020) ^S9^ | 34 Physically active (25.5± 5.1 years) | Not reported | Testing days participants ingested a standardized meal (66.2 g/kg of CHO - 24.9 g of protein - 6.0 g of fat) 120 min pre-exercise | EC1: Oatmeal  EC2: PLA | 120 min pre-exercise:  EC1: 650 ml of water and semi-skimmed milk with oatmeal (1.0 g/kg CMHO)  EC2: 650 ml of water and semi-skimmed milk | Acute (CODn) | At the rest and post-exercise (HIIT of 30 min): Reactive oxygen species, glucose, BLA and RPE | Comparison EC1 vs EC2: ↑Glucose pre-exercise  ↓Reactive oxygen species post-exercise |
| Haakonssen (2015) ^S10^ | 32 Competitive cyclists (24.3±4.1 years) | Not reported | Participants ingested a standardised diet the prior 24-h before trial (5 g/kg of CHO - 1.5 g/kg of protein - 1.5 g/kg of fat). Testing days, participants ingested an isocaloric diet with the same macronutrient distribution | EC1: Calcium-rich dairy-based meal  EC2: Control | Breakfast (90 min pre-exercise):  EC1: 1350 mg of calcium  EC2: 50 mg of calcium | Acute (COD) | Pre-, during- and post-exercise (80 min cycling 60% MAP + 10-min TT): haematocrit, ionized calcium, concentrations of parathyroid hormone, cross linked C-telopeptide of type I and type II collagen and procollagen I N-terminal propeptide | Comparison EC1 vs EC2:  During-exercise:  ↑Ionized calcium  ↓Haematocrit and parathyroid hormone concentrations |
| Haakonssen (2014) ^S11^ | 32 Competitive cyclists (24.3±4.1 years) | Not reported | Participants ingested a standardised diet the day before trials (5 g/kg of CHO - 1.5 g/kg of protein - 1.5 g/kg of fat). Testing days, the meals provided to both groups were isocaloric with 2 g/kg of CHO | EC1: Dairy (1350 mg of Ca)  EC2: Control (50 mg of calcium) | Pre-exercise meal:  EC1: Dairy consisted of rolled-oats cooked with calcium-fortified, milk, yogurt and additional milk (1350 mg of Ca)  EC2: Meal that provided oats cooked with water and served with canned fruit and nuts (50 mg of calcium) | Acute (COD) | 80 min cycling 60% MAP + 10-min TT: 10-min TT (power), meal gut comfort (pre- and post-exercise) and VAS palatability | Not differences were reported between ECs |
| Ormsbee (2016) ^S12^ | 10 Team sport players (30±7 years) | Eumenorrheic. Trials were performed in the same phase of the MC | 72-h before testing, participants were instructed for maintaining their habitual diet, but dietary caffeine sources were avoided 24 before trials | EC1: Chocolate milk (CHM)  EC2: PLA | The night before the trials (30 min pre-sleep):  EC1: 355 ml of CHM (30 g CHO - 12 g protein)  EC2: 355 ml of a non-nutritive beverage | Acute (COD) | Submaximal test (5 min at 55% - 60% - 65% VO_2max_) + 10-km TT: 10-km TT (time), VO_2_, RER, glucose, total CHO and lipid oxidation, RPE and urine specific gravity and output (pre- and post-exercise) | Comparison EC1 vs EC2: ↑ RER and total CHO oxidation (submaximal test)  ↓Total fat oxidation (submaximal test) and urine output (pre-exercise) |
| Antonio (2018) ^S13^ | 24 Physically active (EG1: n=14; EG2: n=14; 37.3±8.9 years) | Not reported | Participants were instructed to maintaining their habitual diet | EG1: High protein diet (HPROT)  EG2: control | EG1: >2.2 g/kg/day protein increasing rich dietary sources in protein and/or the consumption of protein powder  EG2: Habitual diet (1.5 g/kg/day protein) | 24 weeks  (PGD) | Body mass, fat mass (kg and %), BLM, total Body Tscore, bone mineral content and mineral density | Not differences were reported for intervention or the interaction time·intervention |
| Campbell (2018) ^S14^ | 17 Resistance trained athletes (EG1: n=8; EG2: n=9; 21.2±2.1 years) | Not reported | Testing days, participants arrived after an overnight fast | EG1: High protein diet (HPROT)  EG2: Low protein diet (LPROT) | EG1: A diet with 2.4 g/kg/day of protein which included 25 g of whey protein pre- and post- every training session. No guidelines place on dietary CHO and fat  EG2: A diet <1.2 g/kg/day of protein which included 5 g of whey protein pre- and post- every training session. No guidelines place on dietary CHO and fat | 8 weeks  (PGD) | 1-RM in squat, deadlift and body composition | ↑ 1-RM squat and deadlift (EG1 and EG2), LBM (EG1) and fat mass (kg and %) (EG1)  Interaction time·intervention: LBM |
| Arciero (2016) ^S15^ | 27 Physically active (EG1: n=12, 42±7 years; EG2: n=15, 42±9 years) | Not reported | Participants ingested a multi-vitamin/mineral complex daily and caffeine (85 mg) on exercise days. Before resistance training and interval exercise, participants ingested a small snack (250 kcal) | EG1: Protein pacing and multi-mode exercise training (PRISE)  EG2: Normal protein and multi-mode exercise training (CON) | Distributed in 5-6 meals daily:  EG1: 2.0 g/kg/day of protein (>0.25 g/kg protein in each meal)  EG2: 1.0 g/kg/day of protein | 12 weeks  (PGD) | 5-km cycling TT, SJ, 1-RM in bench press and leg press, abdominal sit-ups test, push-ups in 1 min, bench throws, flexibility, balance, body composition, resting metabolic rate, glucose, insulin, cholesterol, triglycerides, SBP, SDP and aortic pulse | ↑5-km cycling TT, SJ, 1-RM in bench press and leg press, abdominal sit-ups test, push-ups in 1 min, bench throws, flexibility, balance, total cholesterol, LBM and body, abdominal and hip fat (EG1 and EG2) and aortic pulse (EG1)  ↓SBP, SDP (EG1 and EG2)  Interaction time·intervention: Abdominal sit-ups, bench throws, SDP and aortic pulse |
| Strandberg (2019) ^S16^ | 63 Recreative active older women (EG1: n=21; EG2: n=21; EG3: n=21) | Menopause. It was not controlled MC phase in the study | 24-h dietary intake was recorded prior to the trials for dietary control | EG1: PUFA diet + resistance training (PUFA+RT)  EG2: resistance training (RT)  EG3: control | EG1: Diet adjusted N-6/N-3 ratio <2 and N-3 PUFA ingesting >500 g/week of fish and scafood (44% CHO - 20% protein - 36% fat)  EG2: to follow the habitual diet | 24 weeks  (PGD) | Type of muscle fibre, CSA, myonuclei/fibree, satellite cellC/fibre  I fiber (%) / O2- Type IIa fiber (%) / O3- Type I fiber CSA (%) / O4- Type IIa fiber CSA (%) | ↑ CSA type IIA (EG1)  No differences were reported for the interaction time·intervention |
| De Souza (2022) ^S17^ | 33 Physically active (EG1: n=17, 21.3±0.5 years; EG2: n=16, 20.7±0.5 years) | Oligomenorrheic and amenorrhoeic | A dietitian reviewed dietary log, eating behaviour and nutritional characteristics | EG1: Hyperenergetic diet  EG2: habitual energy intake | EG1: Hyperenergetic diet (+20-40% above baseline energy needs)  EG2: habitual energy intake | 1 year (PGD) | BMD (femoral neck, total hip and total body), body composition, Total IGF-1, leptin, triiodothyronine and menstrual recovery | ↑BMD total body and body fat (%)  (EG1 and EG2), body mass, BMI, body fat (kg) and IGF-1 (EG1)  ↓BMD femoral (EG1 and EG2)  Interaction time·intervention: body fat (kg and %), IGF-1, leptin and triiodothyronine |
| Miralles-Amoros (2023) ^S18^ | 14 Professional handball players (EG1: n=7, 21±3 years; EG2: n=7, 22±4 years) | Not reported | Distribution of macronutrients for EGs was as follows: 7.0 g/kg/day of CHO - 1.5 g/kg of protein – 30% energetic intake were lipids. to monitor diet, a 7-day self-record was documented three times along the study | EG1: High-antioxidant diet (HAD)  EG2: Mediterranean Diet (MD)  EG3: control | EG1: Diet prioritised the inclusion of fruits high in antioxidants, such as blueberries, beetroot and pomegranate, providing 200% of the RDA for vitamin A, C and E  EG2: Diet provided 100% of the RDA for vitamin A, C and E  EG3: Free diet with healthy lifestyle recommendations for sportswomen | 12 weeks  (PGD) | Eating behaviour, body image (BSQ) and mood (POMS) | ↓Body image, tension, vigour and depression (EG1, EG2 and EG3)  No differences were reported for the interaction time·intervention |

BLA: blood lactate concentration; BLM: body lean mass; CMJ: counter movement jump; COD: crossover design; CSA: fibre cross-sectional area;; FFA: plasma free fatty acid; HIIT: High-intensity interval training; MAP: maximal aerobic power; MC: menstrual cycle; MIN: minutes; PGD: parallel group design; PLA: placebo; RER: respiratory exchange ratio; RM: repetition maximum; RPE: rate of perceived exertion; RTF: repetition to failure; SBP: systolic blood pressure; SDP: diastolic blood pressure; SJ: squat jump; TT: time trial tests; TTE: time-to-exhaustion test; VO2: volume of oxygen; VO2max: maximum oxygen volume consumption

1. Walker JL, Heigenhauser GJ, Hultman E, Spriet LL. Dietary carbohydrate, muscle glycogen content, and endurance performance in well-trained women. *J Appl Physiol (1985)*. 2000;88(6):2151-2158. doi:10.1152/jappl.2000.88.6.2151
2. Reznik Dolins K, Boozer CN, Stoler F, Bartels M, DeMeersmane R, Contento I. Effect of variable carbohydrate intake on exercise performance in female endurance cyclists*. Int J Sport Nutr Exerc Metab.* 2003;13(4):422-435. doi:10.1123/ijsnem.13.4.422
3. Martínez-Rodríguez A, Rubio-Arias JA, García-De Frutos JM, Vicente-Martínez M, Gunnarsson TP. Effect of High-Intensity Interval Training and Intermittent Fasting on Body Composition and Physical Performance in Active Women. *Int J Environ Res Public Health*. 2021;18(12):6431. Published 2021 Jun 14. doi:10.3390/ijerph18126431
4. Vargas-Molina S, Gómez-Urquiza JL, García-Romero J, Benítez-Porres J. Effects of the Ketogenic Diet on Muscle Hypertrophy in Resistance-Trained Men and Women: A Systematic Review and Meta-Analysis. *Int J Environ Res Public Health*. 2022;19(19):12629. Published 2022 Oct 3. doi:10.3390/ijerph191912629
5. McLay RT, Thomson CD, Williams SM, Rehrer NJ. Carbohydrate loading and female endurance athletes: effect of menstrual-cycle phase. *Int J Sport Nutr Exerc Metab*. 2007;17(2):189-205. doi:10.1123/ijsnem.17.2.189
6. Stevenson EJ, Williams C, Mash LE, Phillips B, Nute ML. Influence of high-carbohydrate mixed meals with different glycemic indexes on substrate utilization during subsequent exercise in women. *Am J Clin Nutr*. 2006;84(2):354-360. doi:10.1093/ajcn/84.1.354
7. Stevenson E, Williams C, Nute M, Humphrey L, Witard O. Influence of the glycaemic index of an evening meal on substrate oxidation following breakfast and during exercise the next day in healthy women. *Eur J Clin Nutr*. 2008;62(5):608-616. doi:10.1038/sj.ejcn.1602759
8. Wynne JL, Ehlert AM, Wilson PB. Effects of high-carbohydrate versus mixed-macronutrient meals on female soccer physiology and performance. *Eur J Appl Physiol*. 2021;121(4):1125-1134. doi:10.1007/s00421-021-04597-5
9. Zeng Z, Jendricke P, Centner C, Storck H, Gollhofer A, König D. Acute Effects of Oatmeal on Exercise-Induced Reactive Oxygen Species Production Following High-Intensity Interval Training in Women: A Randomized Controlled Trial. *Antioxidants (Basel)*. 2020;10(1):3. Published 2020 Dec 22. doi:10.3390/antiox10010003
10. Haakonssen EC, Ross ML, Knight EJ, et al. The effects of a calcium-rich pre-exercise meal on biomarkers of calcium homeostasis in competitive female cyclists: a randomised crossover trial. *PLoS One*. 2015;10(5):e0123302. Published 2015 May 13. doi:10.1371/journal.pone.0123302
11. Haakonssen EC, Ross ML, Cato LE, et al. Dairy-based preexercise meal does not affect gut comfort or time-trial performance in female cyclists. *Int J Sport Nutr Exerc Metab*. 2014;24(5):553-558. doi:10.1123/ijsnem.2014-0069
12. Ormsbee MJ, Gorman KA, Miller EA, et al. Nighttime feeding likely alters morning metabolism but not exercise performance in female athletes. *Appl Physiol Nutr Metab*. 2016;41(7):719-727. doi:10.1139/apnm-2015-0526
13. Antonio J, Ellerbroek A, Evans C, Silver T, Peacock CA. High protein consumption in trained women: bad to the bone?. *J Int Soc Sports Nutr*. 2018;15:6. Published 2018 Jan 31. doi:10.1186/s12970-018-0210-6
14. Campbell BI, Aguilar D, Conlin L, et al. Effects of High Versus Low Protein Intake on Body Composition and Maximal Strength in Aspiring Female Physique Athletes Engaging in an 8-Week Resistance Training Program [published correction appears in Int J Sport Nutr Exerc Metab. 2020 Sep 1;30(5):383]. *Int J Sport Nutr Exerc Metab*. 2018;28(6):580-585. doi:10.1123/ijsnem.2017-0389
15. Arciero PJ, Ives SJ, Norton C, et al. Protein-Pacing and Multi-Component Exercise Training Improves Physical Performance Outcomes in Exercise-Trained Women: The PRISE 3 Study. *Nutrients*. 2016;8(6):332. Published 2016 Jun 1. doi:10.3390/nu8060332
16. Strandberg E, Ponsot E, Piehl-Aulin K, Falk G, Kadi F. Resistance Training Alone or Combined With N-3 PUFA-Rich Diet in Older Women: Effects on Muscle Fiber Hypertrophy. *J Gerontol A Biol Sci Med Sci*. 2019;74(4):489-494. doi:10.1093/gerona/gly130
17. De Souza MJ, Ricker EA, Mallinson RJ, et al. Bone mineral density in response to increased energy intake in exercising women with oligomenorrhea/amenorrhea: the REFUEL randomized controlled trial. *Am J Clin Nutr*. 2022;115(6):1457-1472. doi:10.1093/ajcn/nqac044
18. Miralles-Amorós L, Vicente-Martínez M, Martínez-Olcina M, et al. Study of Different Personalised Dietary Plans on Eating Behaviour, Body Image and Mood in Young Female Professional Handball Players: A Randomised Controlled Trial. *Children (Basel)*. 2023;10(2):259. Published 2023 Jan 31. doi:10.3390/children10020259
