# Supplementary material for: Nutritional Strategies for Optimizing Health, Sports Performance, and Recovery for Female Athletes and Other Physically Active Women: A Systematic Review
Source: Nutr Rev. 2024 Jul 12;83(3):e1068–89. doi: 10.1093/nutrit/nuae082 (PMC11819490; doi:10.1093/nutrit/nuae082)
Supplement: nuae082_Supplementary_Data [file nuae082_supplementary_data.zip › nuae082_Supplementary_Data/SupplementaryMaterialS4_new.docx]

**Supplementary Material S4.** **Summarize of studies included in the systematic review focused on interventions based on manipulation of dietary supplements for prevent or treat nutritional deficiencies**

| Reference | Population | Control of menstrual function | Dietary control | Intervention | | | Outcomes | Results |
| --- | --- | --- | --- | --- | --- | --- | --- | --- |
|  |  |  |  | Experimental group/conditions | Characteristics | Duration |  |  |
| Dellavalle (2014) ^S19^ | 40 Iron-depleted competitive rowers (EG1: n=21, 19.7±0.9 years; EG2: n=19, 19.8±1.1 years) | Not reported | 7-days dietary intake was recorded prior to the trials for dietary control | EG1: Fe  EG2: PLA | EG1: 100 mg/day of FeSO4 (2 x 50 mg)  EG2: 100 mg/day of PLA (2 x 50 mg) | 6 weeks (PGD) | 4-km TT (min, VO_2peak_, maximal work rate, HR, RER, gross energetic, total energy expenditure and BLA), hemoglobin, log Fe status, soluble transferrin, total body iron | ↑4-km TT (VO_2peak_ and total energy expenditure) and hemoglobin  ↓4-km TT (BLA)  Interaction time· intervention: 4-km TT (total energy expenditure) |
| Sandroni (2022) ^S20^ | 19 National competitive basketaball players (EG1: n=9, 19.9±1.6 years; EG2: n=10, 19.9±1.6 years) | Not reported | Fe was ingested with orange juice while symbiotic was ingested in the breakfast. Any other dietary supplement was avoided along the study | EG1: Fe + symbiotic  EG2: Fe + PLA | EG1: 140 mg of FeSO4 + synbiotic (5 g prebiotic fiber + 8 billion CFU probiotic B. Lactis)  EG2: 140 mg of FeSO4 + PLA | 8 weeks (PGD) | Hemoglobin, ferritin and log serum ferritin | ↑Log serum ferritin (EG1 and EG2)  Interaction time· intervention: Log serum ferritin |
| McClung (2009) ^S21^ | 171 soldiers (EG1: n=86, 20.4±4.2 years; EG2: n=85, 20.8±4.4 years) | Not reported | Not reported | EG1: Fe  EG2: PLA | EG1: 140 mg of FeSO4  EG2: 140 mg of PLA | 8 weeks (PGD) | After a basic combat training (BCT): 2 Miles running (time) / hemoglobin, RDW (%), ferritin, transferrin saturation, soluble transferrin receptor, POMS | ↑Hemoglobin, RDW (%), iron and soluble transferrin receptor (EG1 and EG2)  ↓Ferritin (EG2)  Interaction time· intervention: Hemoglobin, RDW (%) and soluble transferrin receptor |
| (Hoch 2009) ^S22^ | 13 Trained runners (EG1: n=8, 25.0±1.4 years; EG2: n=5, 22.4±0.9 years) | Eumenorrheics. Trials were performed during the follicular phase | Testing days, participants arrived after an overnight fast | EG1: Folic acid  EG2: PLA | EG1: 10 mg/day of folic acid  EG2: 10 mg/day of PLA | 6 weeks (PGD) | SBP, DBP, peak brachial artery diameter, flow-mediated dilation and Peak change in flow velocity (%) | Interaction time· intervention: Flow-mediated dilation |
| Taghiyar (2013a) ^S23^ | 64 Trained athletes (EG1: n=14, 33.9±1.5 years; EG2: n=16, 31.3±1.8 years; EG3: n=13, 38.5±1.6 years; EG4: n=15, 33.9±1.5 years) | Not reported | Participants were instructed to maintaining their habitual diet. 48-h dietary intake was recorded two times for dietary control | EG1: Vitamin C + vitamin E (VitC+E)  EG2: Vitamin C (VitC)  EG3: Vitamin E (VitE)  EG4: PLA | EG1: 250 mg/day of vitamin C + 400 IU/day of vitamin E  EG2: 250 mg/day of vitamin C  EG3: 400 IU/day of vitamin E  EG4: PLA | 4 weeks (PGD) | VO_2max_, fat mas (%) and myoglobin | ↑Myoglobin (EG2) |
| Taghiyar (2013b) ^S24^ | 64 Trained athletes (EG1: n=14, 33.9±1.5 years; EG2: n=16, 31.3±1.8 years; EG3: n=13, 38.5±1.6 years; EG4: n=15, 33.9±1.5 years) | Not reported | Participants were instructed to maintaining their habitual diet. 48-h dietary intake was recorded two times for dietary control | EG1: Vitamin C + vitamin E (VitC+E)  EG2: Vitamin C (VitC)  EG3: Vitamin E (VitE)  EG4: PLA | EG1: 250 mg/day of vitamin C + 400 IU/day of vitamin E  EG2: 250 mg/day of vitamin C  EG3: 400 IU/day of vitamin E  EG4: PLA | 4 weeks (PGD) | Aspartate transaminase, LDH, MDA and CK | ↑Aspartate transaminase (EG1) and CK (EG4)  ↓MDA (EG1, EG2, EG3 and EG4), CK (EG2 and EG3) and LDH (EG3)  ↓CK and MDA (1-2) |

BLA: blood lactate concentration; CK: creatin kinase; COD: crossover design; HR: heart rate; LDH: lactate dehydrogenase; MDA: malondialdehyde; MIN: minutes; PGD: parallel group design; PLA: placebo; RDW: red blood cell distribution width; RER: respiratory exchange ratio; TT: time trial tests; VO2: volume of oxygen; VO2max: maximum oxygen volume consumption

S19. DellaValle DM, Haas JD. Iron supplementation improves energetic efficiency in iron-depleted female rowers. Med Sci Sports Exerc. 2014;46(6):1204-1215. doi:10.1249/MSS.0000000000000208

S20. Sandroni A, House E, Howard L, DellaValle DM. Synbiotic Supplementation Improves Response to Iron Supplementation in Female Athletes during Training. J Diet Suppl. 2022;19(3):366-380. doi:10.1080/19390211.2021.1887423

S21. McClung JP, Karl JP, Cable SJ, et al. Randomized, double-blind, placebo-controlled trial of iron supplementation in female soldiers during military training: effects on iron status, physical performance, and mood. Am J Clin Nutr. 2009;90(1):124-131. doi:10.3945/ajcn.2009.27774

S22. Hoch AZ, Pajewski NM, Hoffmann RG, Schimke JE, Gutterman DD. Possible relationship of folic Acid supplementation and improved flow-mediated dilation in premenopausal, eumenorrheic athletic women. J Sports Sci Med. 2009;8(1):123-129.

S23. Taghiyar M, Darvishi L, Askari G, et al. The effect of vitamin C and e supplementation on muscle damage and oxidative stress in female athletes: a clinical trial. Int J Prev Med. 2013;4(Suppl 1):16-23.

S24. Taghiyar M, Ghiasvand R, Askari G, et al. The effect of vitamins C and e supplementation on muscle damage, performance, and body composition in athlete women: a clinical trial. Int J Prev Med. 2013;4(Suppl 1):24-30.
