# Supplementary material for: Nutritional Strategies for Optimizing Health, Sports Performance, and Recovery for Female Athletes and Other Physically Active Women: A Systematic Review
Source: Nutr Rev. 2024 Jul 12;83(3):e1068–89. doi: 10.1093/nutrit/nuae082 (PMC11819490; doi:10.1093/nutrit/nuae082)
Supplement: nuae082_Supplementary_Data [file nuae082_supplementary_data.zip › nuae082_Supplementary_Data/SupplementaryMaterialS5_new.docx]

**Supplementary Material S5***.* **Summarize of studies included in the systematic review focused on interventions based on manipulation of dietary supplements for providing a practical form of energy and nutrients**

| Reference | Population | Control of menstrual function | Dietary control | Intervention | | | Outcomes | Results |
| --- | --- | --- | --- | --- | --- | --- | --- | --- |
|  |  |  |  | Experimental group/conditions | Characteristics | Duration |  |  |
| Mock (2021) ^S25^ | 10 Competitive cyclists (25.7±5.0 years) | Not reported | Participants were asked to keep a 48-h food diary before their first trial. They replicated that diet before the second trial | EC1: CHO  EC2: Vitargo  EC3: PLA | EC1: 700 ml water with 1.2 g/kg of CHO (maltodextrin/dextrose)  EC2: 700 ml water with 1.2 g/kg of Vitargo  EC3: 700 ml waterr with 1.2 g/kg of PLA | Acute (COD) | Post-exercise for  Inducing muscle glycogen (TTE 75% VO_2max_): TT-15 min (power and distance cover, RER), salivary estradiol and total body water | Comparison EC1 and EC2 vs EC3: ↑Power and distance cover in TT-15 |
| McCleave (2011) ^S26^ | 14 Cyclist and triathletes trained (local level) (30.4±1.6 years) | Not reported | Before the first trial, 48-h dietary intake was recorded for replicating it prior to the next trials | EC1: CHO + PRO  EC2: CHO | EC1: 275 mL water with 3 g CHO + 1.2 g PRO every 20 min  EC2: 275 mL water with 3 g CHO every 20 min | Acute (COD) | Submaximal 3-h cycling test between 45-75% VO_2max_ + TTE at 75% VO_2max_: time, VO_2_, RER, rate of CHO and fat oxidation, RPE, BLA, glucose and insulin | Comparison EC1 vs EC2: ↑TTE (time, VO_2_,)  ↓Glucose post-exercise |
| Hida (2012) ^S27^ | 30 Athletes from the Japan College of Physical Education (EG1: n=15; EG2: n=15) | Eumenorrheic. The intervention started at the beginning of the follicular phase | Participants were instructed to maintaining their habitual diet avoiding dietary supplement | EG1: Egg protein (PRO)  EG2: PLA | EG1: 15 g /day of egg protein  EG2: 17.5 g/day of PLA (maltodextrin) | 8 weeks (PGD) | 1 RM in leg extension, leg curl, squat and bench press, body composition, blood glucose, insulin, triglyceride, albumin, creatine phosphokinase activity, aspartate aminotransferase activity, alanine aminotransferase activity, cortisol, growth hormone and myoglobin | ↑1-RM (leg extension, leg curl, squat and bench press), BLM and blood glucose (EG1 and EG2)  ↓Body fat (%), growth hormone and myoglobin (EG1 and EG2)  Not differences were reported for the interaction time·intervention |
| Taylor (2016) ^S28^ | 16 Resistance trained basketball players (EG1: n=8, 20±2 years; EG2: n=8, 21±3 years) | Not reported | Participants were asked to keep a 96-h food diary before their first trial. They replicated that diet before the second trial | EG1: Whey protein (PRO)  EG2: PLA | EG1: 24 g whey protein pre-exercise  EG2: 24 g/day of PLA (maltodextrin) | 8 weeks (PGD) | 1-RM in bench press and leg press, vertical jump, broad jump, 5-10-4 agility drill and body composition | ↑ 1-RM (bench press and leg press) vertical jump, broad jump, 5-10-4 agility drill and BLM (EG1 and EG2)  ↓Body fat (%)  Interaction time·intervention: 1-RM in bench press and BLM |
| Wilborn (2016) ^S29^ | 17 Resistance trained (EG1: n=8, 22±2 years; EG2: n=9, 20±3 years) | Not reported | 96-h dietary intake was recorded three times along the study for dietary control | EG1: PRO + creatine  EG2: PRO | EG1: 25 g whey protein + 5 g monohydrate creatine (4 days/week)  EG2: 25 g whey protein (4 days/week) | 8 weeks (PGD) | 1-RM in bench and leg press, RTF at 65% 1-RM in bench press and leg press, vertical jump, broad jump, Wingate test (mean and peak power) and body composition | ↑1-RM (bench and leg press), RTF in leg press, vertical jump, broad jump, Wingate test (peak power), body mass, BLM, appendicular lean mass and total arm and leg mass (EG1 and EG2) |
| Gratwicke (2023) ^S30^ | 16 semi-professional female rugby union players (EG1: n=8; EG2: n=8) | Not reported | Not reported | EG1: α-lactalbumin  EG2: Whey protein (PRO) | EG1: 40 g of α- lactalbumin protein powder  EG2: 40 g of whey protein powder | 3 weeks (PGD) | Total sleep time, wake after and sleep onset latency | ↓Sleep onset latency (EG1)  Interaction time· intervention: Sleep onset latency |
| Miles (2021) ^S31^ | 16 competitive athletes (10 team sport athletes, 4 middle distance runners and 2 weight-lifting) (27±7 years) | Eumenorrheic. It was not controlled MC phase in the study | Testing days participants ingested a standardized meal (66.2 g of CHO - 24.9 g of protein - 6.0 g of fat) | EG1: α-lactalbumin competition (LACT-C) and no competition days (LACT-NC)  EG2: Whey protein (PRO) (PRO-C) and no competition days (PRO-NC)  EG3: control (C) and no competition days (NC) | 120 min before bed-time:  EG1: 400 ml water with 40 g of α-lactalbumin protein powder  EG2: 400 ml water with 40 g of whey protein powder  EG3: 400 ml water | Acute (COD) | CMJ, YYRR1, total sleep time, wake after sleep onset, time spent in wake, rapid eye movement and non–rapid eye movement | Interaction time·intervention: YYRR1 and non–rapid eye movement |

BLA: blood lactate concentration; BLM: body lean mass; CMJ: counter movement jump; COD: crossover design; MIN: minutes; PGD: parallel group design; PLA: placebo; RER: respiratory exchange ratio; RM: repetition maximum; RPE: rate of perceived exertion; RTF: repetition to failure; TT: time trial tests; TTE: time-to-exhaustion test; VO2: volume of oxygen; VO2max: maximum oxygen volume consumption;

S25. Mock MG, Hirsch KR, Blue MNM, Trexler ET, Roelofs EJ, Smith-Ryan AE. Post-Exercise Ingestion of Low or High Molecular Weight Glucose Polymer Solution Does Not Improve Cycle Performance in Female Athletes. J Strength Cond Res. 2021;35(1):124-131. doi:10.1519/JSC.0000000000002560

S26. McCleave EL, Ferguson-Stegall L, Ding Z, et al. A low carbohydrate-protein supplement improves endurance performance in female athletes. J Strength Cond Res. 2011;25(4):879-888. doi:10.1519/JSC.0b013e318207e98c

S27. Hida A, Hasegawa Y, Mekata Y, et al. Effects of egg white protein supplementation on muscle strength and serum free amino acid concentrations. Nutrients. 2012;4(10):1504-1517. Published 2012 Oct 19. doi:10.3390/nu4101504

S28. Taylor LW, Wilborn C, Roberts MD, White A, Dugan K. Eight weeks of pre- and postexercise whey protein supplementation increases lean body mass and improves performance in Division III collegiate female basketball players. Appl Physiol Nutr Metab. 2016;41(3):249-254. doi:10.1139/apnm-2015-0463

S29. Wilborn CD, Outlaw JJ, Mumford PW, et al. A Pilot Study Examining the Effects of 8-Week Whey Protein versus Whey Protein Plus Creatine Supplementation on Body Composition and Performance Variables in Resistance-Trained Women. Ann Nutr Metab. 2016;69(3-4):190-199. doi:10.1159/000452845

S30. Gratwicke M, Miles KH, Clark B, Pumpa KL. The effect of α-lactalbumin consumption on sleep quality and quantity in female rugby union athletes: a field-based study. Biol Sport. 2023;40(2):449-455. doi:10.5114/biolsport.2023.116002

S31. Miles KH, Clark B, Fowler PM, et al. ɑ-Lactalbumin Improves Sleep and Recovery after Simulated Evening Competition in Female Athletes. Med Sci Sports Exerc. 2021;53(12):2618-2627. doi:10.1249/MSS.0000000000002743
