# Supplementary material for: Nutritional Strategies for Optimizing Health, Sports Performance, and Recovery for Female Athletes and Other Physically Active Women: A Systematic Review
Source: Nutr Rev. 2024 Jul 12;83(3):e1068–89. doi: 10.1093/nutrit/nuae082 (PMC11819490; doi:10.1093/nutrit/nuae082)
Supplement: nuae082_Supplementary_Data [file nuae082_supplementary_data.zip › nuae082_Supplementary_Data/SupplementaryMaterialS6_new.docx]

**Supplementary Material S6***.* **Summarize of studies included in the systematic review focused on interventions based on manipulation of dietary supplements for improving performance**

| Reference | Population | Control of menstrual function | Dietary control | Intervention | | | Outcomes | Results |
| --- | --- | --- | --- | --- | --- | --- | --- | --- |
|  |  |  |  | Experimental group/conditions | Characteristics | Duration |  |  |
| Lara (2014) ^S32^ | 18 Competitive soccer players (21±2 years) | Not reported | Not reported | EC1: Caffeine  EC2: PLA | 60 min pre-exercise: EC1: 3 mg/kg of powdered caffeine  EC2: 3 mg/kg of PLA | Acute (COD) | CMJ, 7x30-m sprint test and in a 2 x 40 min simulated match: Total distance covered (m), between 0.5-3.0 km/h, 3.1-8.0 km/h, 8.1-13.0 km/h, 13.1-18.0 km/h and > 18.0 km/h | Comparison EC1 vs EC2:  ↑CMJ, 7x30-m sprint test and Total distance covered (m), distance between 3.1-8.0 km/h, 8.1-13.0 and >18.0 km/h |
| Lara (2020) ^S33^ | 13 Competitive triathletes (31±6 years) | Eumenorrheic. Trials were performed in the early and late follicular phase and mid luteal phase | Participants ingested a standardised precompetitive diet/fluid routine 24-h before trials | EC1: Caffeine in the early follicular phase  EC2: PLA in the early follicular phase  EC3: Caffeine in the late follicular phase  EC4: PLA in the late follicular phase  EC5: Caffeine in the mid luteal phase  EC6: PLA in the mid luteal phase | 60 min pre-exercise: EC1: 3 mg/kg of powdered caffeine in the early follicular phase  EC2: 3 mg/kg of PLA (cellulose) (60 min pre-exercise) in the early follicular phase  EC3: idem EC1 (in the late follicular phase)  EC4: idem EC2 (in the late follicular phase)  EC5: idem EC1 (in the mid luteal phase)  EC6: idem EC2 (in the mid luteal phase) | Acute (COD) | In a 15-s Wingate test: Peak and mean power, fatigue index, BLA, RPE and perceived muscle power | Comparison EC1 vs EC2:  ↑15-s Wingate test (peak and mean power) and BLA  Comparison EC3 vs EC4:  ↑15-s Wingate test (peak and mean power)  Comparison EC5 vs EC6:  ↑ 15-s Wingate test (peak and mean power) |
| Romero-Moraleda (2019) ^S34^ | 13 Physically active (31±6 years) | Eumenorrheic. Trials were performed in the early and late follicular phase and mid luteal phase | To avoid nutritional supplements and dietary caffeine sources 24-h before trials | EC1: Caffeine in the early follicular phase  EC2: PLA in the early follicular phase  EC3: Caffeine in the late follicular phase  EC4: PLA in the late follicular phase  EC5: Caffeine in the mid luteal phase  EC6: PLA in the mid luteal phase | 60 min pre-exercise: EC1: 3 mg/kg of caffeine in the early follicular phase  EC2: 3 mg/kg of PLA (cellulose) in the early follicular phase  EC3: idem EC1 (in the late follicular phase)  EC4: idem EC2 (in the late follicular phase)  EC5: idem EC1 (in the mid luteal phase)  EC6: idem EC2 (in the mid luteal phase) | Acute (COD) | Mean and peak velocity at 20%, 40%, 60% and 80% 1-RM in half squat | Comparison EC1 vs EC2:  ↑Mean velocity (60% 1-RM)  Comparison EC3 vs EC4:  ↑Mean (60% 1-RM) and peak velocity (20% 1-RM)  Comparison EC5 vs EC6: Not differences were reported |
| Filip-Stachnik (2020) ^S35^ | 13 Resistance training athletes (23.0±0.8 years) | Not reported | Participants were instructed to maintaining their habitual diet. However, it was avoided any dietary caffeine sources 12 hour before trials | EC1: Caffeine  EC2: PLA | 60 mins pre-exercise: EC1: 3 mg/kg of caffeine EC2: EC1: 3 mg/kg of PLA | Acute (COD) | 1-RM and RTF at 50% 1-RM (number of repetitions, time under tension, mean and peak power and mean and peak velocity) in bench press | Not differences were reported between ECs |
| Ali (2015) ^S36^ | 10 Trained sport team players (24±4 years) | Eumenorrheic. Participants taking monophasic oral contraceptive | Participants were asked to keep a 48-h food diary before their first trial. They replicated that diet before the second trial. In this period, dietary caffeine sources were avoided | EC1: caffeine  EC2: PLA | 60 min pre-exercise: EC1: 6 mg/kg of anhydrous caffeine  EC2: 6 mg/kg of PLA (artificial sweetener) | Acute (COD) | Post-exercise (6x15 min): Sleep quality | Comparison EC1 vs EC2:  ↑Sleep latency, to get to sleep, restless sleep and periods of wakefulness |
| Ali (2016) ^S37^ | 10 Trained sport team players (24±4 years) | Eumenorrheic. Participants taking monophasic oral contraceptive. Trials were performed 5–8 and 18–22 days of the one pill-cycle | Participants were asked to keep a 48-h food diary before their first trial. They replicated that diet before the second trial. In this period, dietary caffeine sources were avoided | EC1: caffeine  EC2: PLA | 45 min pre-exercise: EC1: 6 mg/kg of anhydrous caffeine EC2: 6 mg/kg of PLA (artificial sweetener) | Acute (COD) | Pre-, during and post-exercise (6 x 15 min): CMJ, concentric and eccentric isokinetic torque and power on knee flexion and extension , VO_2_, glucose, insulin, FFA / O15- RPE | Comparison EC1 vs EC2:  During-exercise:  ↑Knee flexor eccentric torque and knee extensor power  Post-exercise:  ↑Knee flexor eccentric power |
| Norum (2020) ^S38^ | 15 Resistance training athletes (29.8±5 years) | Eumenorrheics. Trials were performed in the early follicular phase | Testing days participants ingested a standardized meal (0.35 g/kg of CHO – 0.36 g of protein) 45 min pre-exercise  Participants were asked to keep a 24-h food diary before their first trial. They replicated that diet before the second trial. Additionally, it was given a list of products enriched in caffeine for avoiding its consumption along the 24-h before each trial | EC1: caffeine  EC2: PLA | 60 min pre-exercise: EC1: 4 mg/kg of anhydrous caffeine  EC2: 4 mg/kg of PLA | Acute (COD) | CMJ, peak torque and RFD on knee extensors, 1-RM and RTF at 60% 1-RM in squat and bench press, and RPE | Comparison EC1 vs EC2: ↑ Peak torque on knee extensors, 1-RM and RTF in squat and bench press, |
| Bougrine (2023) ^S39^ | 13 Elite handball players (16.6±0.5 years) | Not reported | Before the first trial, 24-h dietary intake was recorded for replicating it prior to the next trials | EC1: caffeine before Ramadan in the morning (CAF-BR-M)  EC2: PLA before Ramadan in the morning (PLA-BR-M)  EC3: caffeine before Ramadan in the evening (CAF-BR-M)  EC4: PLA before Ramadan in the evening (PLA-BR-E)  EC5: caffeine post-Ramadan in the morning (CAF-PR-M)  EC6: PLA post-Ramadan in the morning (PLA-PR-M)  EC7: caffeine post-Ramadan in the evening (CAF-PR-E)  EC8: PLA post-Ramadan in the evening (PLA-PR-E) | 60 min pre-exercise: EC1: 6 mg/kg of caffeine  EC2: 6 mg/kg of PLA (cellulose) | Acute (COD) | SJ, agility test and 6x30 second running | Comparison EC1 vs EC2: ↑SJ, agility test and 6x30 second running  Comparison EC3 vs EC4: ↑ Agility test and 6x30 second running  Comparison EC5 vs EC6: ↑ Agility test and 6x30 second running  Comparison EC7 vs EC8: ↑Agility test and 6x30 second running |
| Karayigit (2020) ^S40^ | 29 Physically active (23±2 years) | Not reported | Not reported | EC1: caffeine 6 mg (CAF-6)  EC2: caffeine 3 mg (CAF-3)  EC3: PLA | 60 min pre-exercise: EC1: 6 mg/kg of caffeine provided from coffee  EC2: 3 mg/kg of caffeine provided from coffee  E3: decaffeinated coffee | Acute (COD) | 3 x RTF at 40% 1-RM in squat and bench press, glucose, BLA, pain perception, HRV, reaction time and arousal | Comparison EC1 vs EC3: ↑RTF in squat, reaction time, BLA and arousal  ↓Pain perception  Comparison C2 vs EC3: ↑BLA and arousal  Comparison EC1 vs EC2: Not differences were reported |
| Pereira (2021) ^S41^ | 17 Sport team players (24±4 years) | Not reported | Not reported | EC1: caffeine + mouth rinsing with CHO (CAF+CHO)  EC2: caffeine + mouth rinsing with PLA (CAF+PLA)  EC3: PLA + mouth rinsing with CHO (PLA+CHO)  EC4: PLA + mouth rinsing with PLA (PLA+ PLA) | EC1: 6.5 mg/kg of caffeine (60 min pre-exercise) + mouth rinsing (10 s with 6 g of CHO, maltodextrin)  EC2: 6.5 mg/kg of caffeine (60 min pre-exercise) + mouth rinsing (10 s with 6 g of PLA)  EC3: 6.5 mg/kg of PLA (cellulose) (60 min pre-exercise) + mouth rinsing (10 s with 6 g of CHO, maltodextrin)  EC4: 6.5 mg/kg of PLA (cellulose) (60 min pre-exercise) + mouth rinsing (10 s with PLA) | Acute (COD) | 3 x RTF at 10-RM: in squat, leg press, bench press, shoulder press, row and RPE | Comparison EC1 – EC2 – EC3 vs EC4: ↑ RTF in squat, leg press, bench press, shoulder press and row |
| Gutiérrez-Hellín (2022) ^S42^ | 19 Physically active (26.9±8.7 years) | Eumenorrheic. Trials were performed in the same phase of the MC | Before the first trial, 24-h dietary intake was recorded for replicating it prior the second trial. Two hours before trials, participants ingested 7 ml/kg of water | EC1: p-synephrine  EC2: PLA | 60 min pre-exercise: EC1: 3 mg/kg of p-synephrine  EC2: 4 mg/kg of PLA (cellulose) | Acute (COD) | At the rest: SBP, SBP, tympanic temperature and urine specific gravity  During a gradual cycling test: CHO and fat oxidation rate, maximal fat oxidation, Fatmax energy expenditure and RPE | Comparison EC1 vs EC2: ↑Tympanic temperature at rest |
| Cox (2002) ^S43^ | 12 Elite soccer players (22.1±5.4) | Not reported | Participants ingested a standardised diet one week before trials (7 g/kg of CHO) | EC1: Creatine  EC2: PLA | EC1: 20 g/day (4 x 5 g) of creatine monohydrate  EC2: 20 g/day (4 x 5 g) of PLA | 7 days (COD) | Post-exercise (60 min simulated match): 20-m sprint time, agility run time, precision ball-kicking, RPE, BLA, blood, pH, HR, and body mass | Comparison EC1 vs EC2: ↑20-m sprint time and agility run time and body mass  ↓ HR |
| Ramirez-Campillo (2016) ^S44^ | 30 Soccer players (EG1: n=10, 23.1±3.4; EG2: n=10, 22.9±1.7; EG3: n=10, 22.5±2.1) | Not reported | 24-h dietary intake was recorded prior to the trials for dietary control | EG1: Creatine + plyometric training (CR+PT)  EG2: PLA + plyometric training (PLA+PT)  EG3: PLA | EG1: 20 g/day (4 x 5 g: breakfast - lunch - dinner - before bedtime) 1 week + 5 g/day (lunch) of creatine monohydrate  EG2: 20 g/day (4 x 5 g: breakfast - lunch - dinner - before bedtime) 1 week + 5 g/day (lunch) of PLA EG3: 20 g/day (4 x 5 g: breakfast - lunch - dinner - before bedtime) 1 week + 5 g/day (lunch) of PLA | 6 weeks (PGD) | BM, CMJ, SJ, 20 and 40 cm reactive strength index, RAST, 20-m sprint, change of direction speed and 20-m multi stage shuttle run test | Comparison EG1 EG3: ↑SJ, 20 and 40 cm reactive strength index, RAST and 20-m sprint and BM  Comparison EG1 EG2: ↑40 cm reactive strength index and RAST |
| Gordon (2023) ^S45^ | 39 Physically active (EG1: n=19, 25.5±7.2 years; EG2: n=19, 23.8±4.3 years) | Eumenorrheic. Trials were performed in the follicular and luteal phase | 72-h dietary intake was recorded prior to the trials for dietary control | EG1: Creatine in the follicular (CRF) and luteal phase (CRL)  EG2: PLA in the follicular (CRF) and luteal phase (CRL) | EG1: 20 g/day (4 x 5 g: breakfast - lunch - dinner - before bedtime) of creatine monohydrate in the follicular phase (days 2-8 of the MC) and the luteal phase (days 14-18 of the MC)  EG2: 20 g/day (4 x 5 g: breakfast - lunch - dinner - before bedtime) of PLA in the follicular (days 2-8 of the MC) the luteal phase (days 14-18 of the MC) | 5 days  (COD) | 10 x 10 seconds (30 s recovery) cycling sprint test (mean and peak power, time to reach peak power and fatigue index ), RPE and HRV (pre- and pot-exercise) | ↓Fatigue index in EG1 (CRL)  Not differences were reported for the interaction time·intervention |
| Brooks (2023) ^S46^ | 40 Physically active (EG1: n= 20, 21±1 years; EG2: n= 20, 20±1 years) | Not reported | To avoid nutritional supplements and dietary caffeine sources 12-h before trials | EG1: Creatine  EG2: PLA | EG1: 0.1 g/kg /day of creatine + 0.1 g/kg/day corn-starch  EG2: | 6 weeks  (PGD) | 5 maximal isokinetic hip flexion and extension, vertical jump, medicine ball throw, body composition, depression, anxiety and stress | ↑Total body water, LBM (kg), lower appendicular lean mass (EG1)  ↓BM (EG2)  Interaction time·intervention: LBM (%) |
| Hemmatinafar (2023) ^S47^ | 12 Trained voleiball players (26.0±3.0 years) | Eumenorrheic. Trials were performed in the late follicular phase (day 10 of the MC) | Not reported | EC1: Beetroot juice (BRJ)  EC2: PLA | 120 min pre-exercise:  EC1: 200 ml of BRJ (4 x 50 ml) (2.1 mmol NO_3_^-^)  EC2: 200 ml of PLA (4 x 50 ml) | Acute (COD) | Pree- and post-exercise-induce muscle damage protocol (200 vertical jumps with weighted vests):  Muscle soreness, pressure pain threshold, wall-sit, V-Sit reach flexibility test, vertical jump and swelling around the thigh | Comparison EC1 vs EC2: ↑Wall-sit, V-Sit reach flexibility test, vertical jump and swelling around the thigh (post-exercise)  ↓Muscle soreness (post-exercise) |
| Jurado-Castro (2022) ^S48^ | 14 Physically active (25.4±4.0 years) | Eumenorrheic. It was not controlled MC phase in the study | 24-h prior testing days participants ingested a standardized (60% CHO - 30% fat - 10% proteins). Additionally, it was given a list of products enriched in NO_3_^-^ and caffeine for avoiding its consumption along the 48-h and 24-h before each trial while 24-h prior testing, participants were encouraged to avoid brushing their teeth or using product that could alter their oral microbiota | EC1: Beetroot juice (BRJ)  EC2: PLA | 120 min pre-exercise:  EC1: 70 ml of BRJ (6.4 mmol NO_3_^-^)  EC2: 70 ml of PLA (blackcurrant beverage) | Acute (COD) | CMJ, mean velocity and power at 50% and 75% 1-RM in back squat and 3 x RTF at 75% 1-RM in back, leg press and leg extension | Comparison EC1 vs EC2: ↑CMJ, mean velocity and power at 50% 1-RM in back squat and 3 x RTF in back, leg press and leg extension |
| López-Samanes (2023) ^S49^ | 11 Elite hockey players (22.8±5.1 years) | Eumenorrheic. 11 participants were assessed along the follicular and 6 in the luteal phase | 24-h prior testing days participants ingested a standardized (60% CHO - 30% fat - 10% proteins). Additionally, it was given a list of products enriched in NO_3_^-^ and caffeine for avoiding its consumption along the 48-h and 24-h before each trial while 24-h prior testing, participants were encouraged to avoid brushing their teeth or using product that could alter their oral microbiota | EC1: Beetroot juice (BRJ)  EC2: PLA | 180 min pre-exercise:  EC1: 70 ml of BRJ (6.4 mmol NO_3_^-^)  EC2: 70 ml of PLA (BRJ depletes in NO_3_^-^) | Acute (COD) | CMJ, Isometric handgrip strength, 20-m sprint, repeated sprint ability and 2 x 12.5 min of simulated match: total distance, peak velocity, accelerations and decelerations (number) | Not differences were reported between ECs |
| Glenn (2016) ^S50^ | 17 Masters-aged tennis players (51±9 years) | Menopause | Before the first trial, 24-h dietary intake was recorded for replicating it prior to the next trials | EC1: Citrulline-malate  EC2: PLA | 60 min pre-exercise:  EC1: 8 g of CM + 12 g dextrose  EC2: 12 g dextrose | Acute (COD) | CMJ, handgrip and Wingate test (average, peak and explosive power) | Comparison EC1 vs EC2: ↑Handgrip and Wingate test (peak and explosive power) |
| Gills (2023) ^S51^ | 29 Physically active (23.5±3.1 years) | Not reported | Before the first trial, 24-h dietary intake was recorded for replicating it prior to the next trials | EC1: Citrulline-malate  EC2: PLA | 60 min pre-exercise:  EC1: 8 g of CM + 12 g dextrose  EC2: 12 g dextrose | Acute (COD) | 5 and 50 x maximal isokinetic leg extension: peak torque, total work, torque and RPE | Comparison EC1 vs EC2: ↑total work in the maximum repetition, the first and last third of the test in the 5 x maximal isokinetic leg extension |
| Glenn (2015) ^S52^ | 33 Competitive cyclist master (EG1: n=10, 54±2 years; EG2: n=11, 53±1 years) | Not reported | Testing days, participants arrived after 3-h fasted and dietary caffeine sources 24-h before | EG1: BA  EG2: PLA | EG1: 3.2 g/day BA (4 x 0.8 g)  EG2: 3.2 g/day PLA (4 x 0.8 g) | 4 weeks  (PGD) | 3 x Wingate test: Absolute and relative mean and peak power, BLA and RPE | Interaction time·intervention: RPE |
| Smith (2012) ^S53^ | 24 Physically active (EG1: n=13, 22.0±2.5 years; EG2: n=11, 21.4±1.4 years) | Not reported | Participants were asked to keep a 72-h food diary before their first trial. They replicated that diet before the second trial | EG1: BA  EG2: PLA | EG1: 4.8 g/day BA (3 x 1.6 g)  EG2: 4.8 g/day PLA (3 x 1.6 g) | 4 weeks  (PGD) | Gradual running exercise (VO_2max_ and ventilatory thresholds) and 40 min running at 70-75% VO_2max_: RPE, TAC, SOD, glutathione and 8-isoprostane | ↑VO_2max_ (EG1 and EG2)  ↓TAC and SD (EG1)  Interaction time·intervention: RPE, TAC, SOD, LBM (EG1) and fat mass (kg and %) (EG1) |
| Glenn (2015) ^S54^ | 12 Competitive cyclists (26±1.3 years) | Eumenorrheic. Trials were performed in the luteal phase | Testing days, participants arrived after 6-h fasted and dietary caffeine sources 24-h before | EC1: BA  EC2: PLA | 30 min pre-exercise: EC1: 1.6 g BA + 34 g dextrose  EC2: 34 g dextrose | Acute (COD) | Gradual cycling test + TTE at 120% VO_2max_ and BLA | Comparison EC1 vs EC2: ↑TTE (time and total work)  ↓BLA |
| Rosas (2017) ^S55^ | 25 Soccer players (EG1: n=8, 24.3±2.5 years; EG2: n=8, 22.8±2.1 years; EG3: n=9, 24.0±2.7 years) | Not reported | 24-h dietary intake was recorded prior to the trials for dietary control | EG1: BA + plyometric training (BA+PT)  EG2: PLA + plyometric training (PLA+PT)  EG3: PLA | EG1: 4.8 g/day of BA (6 x 0.8 g interspersed 2 hours)  EG2: 4.8 g/day of PLA (cellulose) (6 x 0.8 g interspersed 2 hours)  EG3: 4.8 g/day of PLA (cellulose) (6 x 0.8 g interspersed 2 hours) | 6 weeks  (PGD) | CMJ, 60 s CMJ, SJ, 20 and 40 cm reactive strength index, RAST, 20-m sprint, change of direction speed and 20-m multistage shuttle run test | Comparison EG1 vs EG3: ↑60 s CMJ, 20-m sprint and RAST  Comparison EG2 vs EG3: ↑20-m sprint |
| Glenn (2016) ^S56^ | 22 Masters-aged cyclists competitors (EG1: n=11, 54±2 years; EG2: n=11, 53±1 years) | Menopause | To avoid dietary caffeine sources 24-h before trials | EG1: BA  EG2: PLA | EG1: 3.2 g/day BA (4 x 0.8 g)  EG2: 3.2 g/day PLA (4 x 0.8 g) | 4 weeks  (PGD) | 50 x maximal isokinetic leg flexion and extension (mean power, time to peak power, total work and fatigue index), isometric strength, isometric grip strength and body composition | Interaction time·intervention: 50 x maximal isokinetic leg flexion (mean power and total work in the third last) and extension (time to peak power and total work and in the third last) |
| Tan (2010) ^S57^ | 12 Elite water polo squad (23.7±3.0 years) | Not reported | Testing days participants ingested a standardized meal (2.0 g/kg of CHO) 120 min pre-exercise | EC1: NaHCO_3_^-^  EC2: PLA | 90 min pre-exercise:  EC1: 0.3 g/kg of NaHCO_3_^-^ with 600 ml of water  EC2: | Acute (COD) | 59 min simulated match (mean sprint times) and BLA, pH and NaHCO_3_^-^ pre- and during-exercise | Comparison EC1 vs EC2: ↑ BLA (during-exercise) and pH and NaHCO_3_^-^ (pre- and during-exercise) |
| Köhne (20116) ^S58^ | 8 Trained runners (EG1: n=4, 28.5±6.5 years; EG2: n=4, 29.5±5.1 years) | Not reported | Participants were asked to keep a 24-h food diary before their first trial. They replicated that diet before the second trial. Any other dietary supplement was avoided since 4 months prior to the start of the study | EG1: Multi-ingredient  EG2: PLA | EG1: 21 g/day of Vital Pharmaceuticals, NO-Shotgun® (18 g protein + 3. 17 g of multiple forms of creatine, BA and caffeine)  EG2: 28 g/day of maltodextrin | 4 weeks (PGD) | Pre- and post-exercise (60 min running at 75% VO_2max_ downhill): CMJ, hamstring flexibility, VAS, pressure pain threshold, IL-6, CK and lower limb circumferences | Not differences were reported for intervention or the interaction time·intervention |
| Cameron (2018) ^S59^ | 15 Recreationally active college-aged (21.5±1.7 years) | Not reported | 48-h dietary intake was recorded prior to the 2 trials for dietary control | EC1: Multi-ingredient  EC2: PLA | 60 min pre-exercise:  EC1: 5.7 g/day of MusclePharm, Fitmiss™pre-workout (BA, choline, L-Tyrosine, L-Glycine, Taurine, L-Carnitine, beetroot extract, hawthorn berry powder, agmatine sulfate and caffeine anhydrous)  EC2:5.7 g of PLA | Acute (COD) | Resting energy expenditure, SBP, DBP, CMJ, RTF 85%1RM in bench press and back squat and during a 25-s treadmill (total work) | Comparison EC1 vs EC2: ↑ Resting energy expenditure, DBP, RTF in bench press |

BA: β-alanina; BLA: blood lactate concentration; CK: creatin kinase; CMJ: counter movement jump; COD: crossover design; ; FFA: plasma free fatty acid; HR: heart rate; HRV: heart rate variability; MC: menstrual cycle; MIN: minutes; NaHCO_3_^-^: sodium bicarbonate; PGD: parallel group design; PLA: placebo; RM: repetition maximum; RPE: rate of perceived exertion; RTF: repetition to failure; SBP: systolic blood pressure; SOD: Superoxide dismutase; SJ: squat jump; TAC: total antioxidant capacity; TT: time trial tests; TTE: time-to-exhaustion test; VO2: volume of oxygen; VO2max: maximum oxygen volume consumption;

S32. Lara B, Gonzalez-Millán C, Salinero JJ, et al. Caffeine-containing energy drink improves physical performance in female soccer players. Amino Acids. 2014;46(5):1385-1392. doi:10.1007/s00726-014-1709-z

S33. Lara B, Gutiérrez Hellín J, Ruíz-Moreno C, Romero-Moraleda B, Del Coso J. Acute caffeine intake increases performance in the 15-s Wingate test during the menstrual cycle. Br J Clin Pharmacol. 2020;86(4):745-752. doi:10.1111/bcp.14175

S34. Romero-Moraleda B, Del Coso J, Gutiérrez-Hellín J, Lara B. The Effect of Caffeine on the Velocity of Half-Squat Exercise during the Menstrual Cycle: A Randomized Controlled Trial. Nutrients. 2019;11(11):2662. Published 2019 Nov 4. doi:10.3390/nu11112662

S35. Filip-Stachnik A, Krzysztofik M, Kaszuba M, et al. Placebo Effect of Caffeine on Maximal Strength and Strength Endurance in Healthy Recreationally Trained Women Habituated to Caffeine. Nutrients. 2020;12(12):3813. Published 2020 Dec 13. doi:10.3390/nu12123813

S36. Ali A, O'Donnell JM, Starck C, Rutherfurd-Markwick KJ. The Effect of Caffeine Ingestion during Evening Exercise on Subsequent Sleep Quality in Females. Int J Sports Med. 2015;36(6):433-439. doi:10.1055/s-0034-1398580

S37. Ali A, O'Donnell J, Foskett A, Rutherfurd-Markwick K. The influence of caffeine ingestion on strength and power performance in female team-sport players. J Int Soc Sports Nutr. 2016;13:46. Published 2016 Dec 5. doi:10.1186/s12970-016-0157-4

S38. Norum M, Risvang LC, Bjørnsen T, et al. Caffeine increases strength and power performance in resistance-trained females during early follicular phase. Scand J Med Sci Sports. 2020;30(11):2116-2129. doi:10.1111/sms.13776

S39. Bougrine H, Nasser N, Abdessalem R, Ammar A, Chtourou H, Souissi N. Pre-Exercise Caffeine Intake Attenuates the Negative Effects of Ramadan Fasting on Several Aspects of High-Intensity Short-Term Maximal Performances in Adolescent Female Handball Players. Nutrients. 2023;15(15):3432. Published 2023 Aug 3. doi:10.3390/nu15153432

S40. Karayigit R, Naderi A, Akca F, et al. Effects of Different Doses of Caffeinated Coffee on Muscular Endurance, Cognitive Performance, and Cardiac Autonomic Modulation in Caffeine Naive Female Athletes. Nutrients. 2020;13(1):2. Published 2020 Dec 22. doi:10.3390/nu13010002

S41. Pereira PEA, Azevedo P, Azevedo K, Azevedo W, Machado M. Caffeine Supplementation or Carbohydrate Mouth Rinse Improves Performance. Int J Sports Med. 2021;42(2):147-152. doi:10.1055/a-1212-0742

S42. Gutiérrez-Hellín J, Aguilar-Navarro M, Ruiz-Moreno C, et al. Effect of p-Synephrine on Fat Oxidation Rate during Exercise of Increasing Intensity in Healthy Active Women. Nutrients. 2022;14(20):4352. Published 2022 Oct 17. doi:10.3390/nu14204352

S43. Cox G, Mujika I, Tumilty D, Burke L. Acute creatine supplementation and performance during a field test simulating match play in elite female soccer players. Int J Sport Nutr Exerc Metab. 2002;12(1):33-46. doi:10.1123/ijsnem.12.1.33

S44. Ramírez-Campillo R, González-Jurado JA, Martínez C, et al. Effects of plyometric training and creatine supplementation on maximal-intensity exercise and endurance in female soccer players. J Sci Med Sport. 2016;19(8):682-687. doi:10.1016/j.jsams.2015.10.005

S45. Gordon AN, Moore SR, Patterson ND, et al. The Effects of Creatine Monohydrate Loading on Exercise Recovery in Active Women throughout the Menstrual Cycle. Nutrients. 2023;15(16):3567. Published 2023 Aug 13. doi:10.3390/nu15163567

S46. Brooks SJ, Candow DG, Roe AJ, et al. Creatine monohydrate supplementation changes total body water and DXA lean mass estimates in female collegiate dancers. J Int Soc Sports Nutr. 2023;20(1):2193556. doi:10.1080/15502783.2023.2193556

S47. Hemmatinafar M, Zaremoayedi L, Koushkie Jahromi M, et al. Effect of Beetroot Juice Supplementation on Muscle Soreness and Performance Recovery after Exercise-Induced Muscle Damage in Female Volleyball Players. Nutrients. 2023;15(17):3763. Published 2023 Aug 28. doi:10.3390/nu15173763

S48. Jurado-Castro JM, Campos-Perez J, Ranchal-Sanchez A, Durán-López N, Domínguez R. Acute Effects of Beetroot Juice Supplements on Lower-Body Strength in Female Athletes: Double-Blind Crossover Randomized Trial. Sports Health. 2022;14(6):812-821. doi:10.1177/19417381221083590

S49. López-Samanes Á, Pérez-Lopez A, Morencos E, et al. Beetroot juice ingestion does not improve neuromuscular performance and match-play demands in elite female hockey players: a randomized, double-blind, placebo-controlled study. Eur J Nutr. 2023;62(3):1123-1130. doi:10.1007/s00394-022-03052-1

S50. Glenn JM, Gray M, Jensen A, Stone MS, Vincenzo JL. Acute citrulline-malate supplementation improves maximal strength and anaerobic power in female, masters athletes tennis players. Eur J Sport Sci. 2016;16(8):1095-1103. doi:10.1080/17461391.2016.1158321

S51. Gills JL, Spliker B, Glenn JM, et al. Acute Citrulline-Malate Supplementation Increases Total Work in Short Lower-Body Isokinetic Tasks for Recreationally Active Females During Menstruation. J Strength Cond Res. 2023;37(6):1225-1230. doi:10.1519/JSC.0000000000004095

S52. Glenn JM, Gray M, Stewart R, et al. Incremental effects of 28 days of beta-alanine supplementation on high-intensity cycling performance and blood lactate in masters female cyclists. Amino Acids. 2015;47(12):2593-2600. doi:10.1007/s00726-015-2050-x

S53. Smith AE, Stout JR, Kendall KL, Fukuda DH, Cramer JT. Exercise-induced oxidative stress: the effects of β-alanine supplementation in women. Amino Acids. 2012;43(1):77-90. doi:10.1007/s00726-011-1158-x

S54. Glenn JM, Smith K, Moyen NE, Binns A, Gray M. Effects of Acute Beta-Alanine Supplementation on Anaerobic Performance in Trained Female Cyclists. J Nutr Sci Vitaminol (Tokyo). 2015;61(2):161-166. doi:10.3177/jnsv.61.161

S55. Rosas F, Ramírez-Campillo R, Martínez C, et al. Effects of Plyometric Training and Beta-Alanine Supplementation on Maximal-Intensity Exercise and Endurance in Female Soccer Players. J Hum Kinet. 2017;58:99-109. Published 2017 Aug 1. doi:10.1515/hukin-2017-0072

S56. Glenn JM, Gray M, Stewart RW Jr, et al. Effects of 28-Day Beta-Alanine Supplementation on Isokinetic Exercise Performance and Body Composition in Female Masters Athletes. J Strength Cond Res. 2016;30(1):200-207.

S57. Tan F, Polglaze T, Cox G, Dawson B, Mujika I, Clark S. Effects of induced alkalosis on simulated match performance in elite female water polo players. Int J Sport Nutr Exerc Metab. 2010;20(3):198-205. doi:10.1123/ijsnem.20.3.198

S58. Köhne JL, Ormsbee MJ, McKune AJ. The effects of a multi-ingredient supplement on markers of muscle damage and inflammation following downhill running in females. J Int Soc Sports Nutr. 2016;13:44. Published 2016 Nov 25. doi:10.1186/s12970-016-0156-5

S59. Cameron M, Camic CL, Doberstein S, Erickson JL, Jagim AR. The acute effects of a multi-ingredient pre-workout supplement on resting energy expenditure and exercise performance in recreationally active females. J Int Soc Sports Nutr. 2018;15:1. Published 2018 Jan 5. doi:10.1186/s12970-017-0206-7
