# Supplementary material for: Nutritional Strategies for Optimizing Health, Sports Performance, and Recovery for Female Athletes and Other Physically Active Women: A Systematic Review
Source: Nutr Rev. 2024 Jul 12;83(3):e1068–89. doi: 10.1093/nutrit/nuae082 (PMC11819490; doi:10.1093/nutrit/nuae082)
Supplement: nuae082_Supplementary_Data [file nuae082_supplementary_data.zip › nuae082_Supplementary_Data/SupplementaryMaterialS7_new.docx]

**Supplementary Material S7***.* **Summarize of studies included in the systematic review focused on interventions based on the manipulation of dietary supplements for improving performance indirectly**

| Reference | Population | Control of menstrual function | Dietary control | Intervention | | | Outcomes | Results |
| --- | --- | --- | --- | --- | --- | --- | --- | --- |
|  |  |  |  | Experimental group/conditions | Characteristics | Duration |  |  |
| Cieślicka (2022) ^S60^ | 20 National level basketball players (EG1: n=11, 17.1±1.2 years; EG2: n=9, 16.0±0.7 years) | Not reported | Not reported | EG1: Bovine colostrum (BC)  EG2: PLA | EC1: 3.2 g/day of CB (1.6 in the morning + 1.6 in the evening)  EC2: 3.2 g/day of PLA (powered milk) (1.6 in the morning + 1.6 in the evening) | 24 weeks (parallel group design) | Running gradual exercise test and pre- and post-exercise: hepcidin, hemopexin, ferritin, UIBC, Fe, IL-6, lactoferrin, transferrin, TIBC, TAC, unsatured iron-binding capacity, Protein carbonyl, TBARS and SOD | ↓IL-6 at rest (EG1 vs EG2) |
| Skarpanska-Stejnbor (2021) ^S61^ | 20 Competitive basketball players (EG1: n=11, 17.1±1.2 years; EG2: n=9, 16.0±0.7 years) | Not reported | Not reported | EG1: Bovine colostrum (BC)  EG2: PLA | EC1: 6.4 g/day of CB (3.2 in the morning + 3.2 in the evening)  EC2: 6.4 g/day of PLA (powered milk) (3.2 in the morning + 3.2 in the evening) | 24 weeks (parallel group design) | Pre and post a gradual running test: IL-1, IL-2, IL-13, IL-10, TNF-alfa, IgG, IGF-1, creatine kinase, white blood cell, lymphocytes, monocytes and granulocytes | Interaction time·intervention· exercise: IL-10 and creatin kinase  ↑ ↓ |
| Brinkworth (2002) ^S62^ | 13 Elite rowers (EG1: n=6, 20.7±4.2, years; EG2: n=7, 20.6±2.3 years) | Not reported | Participants were instructed to maintaining their habitual diet avoiding dietary supplement | EG1: Bovine colostrum (BC)  EG2: Whey protein (PRO) | EC1: 20 g/day of CB (20 g in the morning + 20 g in the evening)  EC2: 6.4 g/day of PRO (powered milk) (3.2 g in the morning + 3.2 g in the evening) | 9 weeks (parallel group design) | Pre- and post-exercise (submaximal and maximal rowing test): Haemoglobin,  plasma buffer capacity and HCO_3_^-^ | ↑Plasma buffer capacity after exercise (EG1 and EG2)  No differences were reported for the interaction time·intervention |
| Brinkworth (2004) ^S63^ | 13 Elite rowers (EG1: n=6, 20.7±4.2, years; EG2: n=7, 20.6±2.6 years) | Not reported | Participants were instructed to maintaining their habitual diet avoiding dietary supplement | EG1: Bovine colostrum (BC)  EG2: Whey protein (PRO) | EC1: 20 g/day of CB (20 g in the morning + 20 g in the evening)  EC2: 6.4 g/day of PRO (powered milk) (3.2 g in the morning + 3.2 g in the evening) | 9 weeks (parallel group design) | 3 x 4 min submaximal test + 4-min rowing TT: distance, VO_2_, mechanical work, BLA, pH and plasma buffer capacity | ↑ Mechanical work in 4-min rowing TT and VO_2_ in submaximal test + 4-min rowing TT (EG1 and EG2) |
| Toohey (2020) ^S64^ | 23 National competitive level of sport team players (EG1: n=11; EG2: n=12) | Not reported | All participants were instructed to avoid any other dietary supplement and special dietary programs | EG1: probiotic (DE111)  EG2: PLA | Post-exercise:  EG1: 45 g CHO - 20 g protein - 2 g fat with probiotic  EG2: 45 g CHO - 20 g protein - 2 g fat PLA | 10 weeks (PGD) | CMJ, 1-RM in squat, deadlift, bench press, isometric midthigh pull (IMTP) strength and RFD, pro-agility, body composition | ↑CMJ, Rectus 1-RM (squat, deadlift and bench press) and femoris thickness (EG1 and EG2)  Interaction group· intervention: body fat (%) |
| McKinley-Barnard (2018) ^S65^ | 22 Physically active (EG1: n=11, 20.7±1.6 years; EG2: n=11, 21.0±1.1 years) | Eumenorrheic | All participants were instructed to avoid fish consumption and any other 6 food which contains EPA and DHA | EG1: Fish oil in the midfollicular (FOF) and midluteal phase (FOL)  EG2: PLA in the midfollicular (PLAF) and midluteal phase (PLAL) | EG1: 6 g/day of fish oil (2.4 g EPA and 1.8 DHA) since the first day of the MC  EG2: 6 g/day of PLA (safflower oil) since the first day of the MC | 3 weeks  (PGD) | 5 x maximal isokinetic knee extension, perceived muscle soreness , myoglobin, TNF-alfa and skeletal muscle variables (NF-κB p50 and NF-κB p65) | Comparison EG1 vs EG2: ↑Perceived muscle soreness  ↓Myoglobin  Inreraction group·cycle of MC: SOD, myoglobin and TNF-alfa |
| Sousa (2020) ^S66^ | 12 Physically active (20.8±1.7 years) | Not reported | To avoid dietary caffeine sources 8-h before trials | EC1: Avocado pulp  EC2: PLA | 60 min pre-exercise:  EC1: 600 mg of avocado pulp  EC2: 600 mg og PLA (starch) | Acute (COD) | Post-exercise (25 min running at 65-70% HR_max_): SBP, DBP, HR and HRV | Comparison EC1 vs EC2: ↑HRV  ↓SBP and HR |
| Bellafiore (2021) ^S67^ | 8 Physically active (23.2±3.0 years) | Eumenorrheic. Trials were performed in the luteal phase | Participants were invited to follow the same dietary plan 1 week before the 2 trials | EC1: Opuntia ficus indica  EC2: PLA | EC1: 50 ml of cold-concentrated OFI diluted with water to 170 ml (in the breakfast)  EC2: 170 ml of a beverage containing the same ingredients of the OFI juice, except for the vitamin C and the bioactive phytochemical (in the breakfast) | 5 days (3 days pre- and 2 days post-trials) (COD) | Gradual cycling test (VO2_max_, power) and hydroperoxides TAC, skin carotenoid score and post-exercise | Comparison EC1 vs EC2: ↓Hydroperoxides, TAC, skin carotenoid score and HRV (pre- and post-exercise) |
| Salehi (2021) ^S68^ | 65 Physically active (EG1: n=32; EG2: n=323) | Not reported | Not reported | EG1: Curcumin  EG2: PLA | EG1: 500 mg/day of curcumin (Curcuma longa L)  EG2: 500 mg/day of PLA | 8 weeks (PGD) | VO_2max_, CRP, TAC, MDA, LDH and body composition | ↑VO_2max_ (EG1)  ↓ CRP, MDA and LDH (EG1)  Interaction time·intervention: VO_2max_, CRP, MDA and LDH |
| Brown (2019) ^S69^ | 20 Physically active (EG1: n=10; EG2: n=10) | Not reported | Participants were asked to keep a 24-h food diary before their first trial. They replicated that diet before the second trial. In this period, any other dietary supplements and dietary caffeine sources were avoided | EG1: Tart cherry  EG2: PLA | EG1: 100 mL of water with 30 mL of concentrate montmorency tart cherry  EG2: 100 ml water with 25 ml of PLA (derived fruit flavoured concentrate with negligible phytochemical content) | 8 days (PGD) | Pre- and post-exercise (15 x 30-m sprints with 10-m deceleration phase): CMJ, DJ, isometric strength, 30-m sprint, flexibility, DOMS, pain pressure threshold, thigh and calf girths, and creatin kinase | Interaction time·intervention: CMJ post-exercise |
| Livolsi (2021) ^S70^ | 15 Competitive softball players (EG1: n=8; EG2: n=7) | Not reported | 72-h dietary intake was recorded prior to the trials for dietary control | EG1: CrPic  EG2: PLA | EG1: 500 ug/day of CrPic  EG2: 500 ug/day of PLA | 6 weeks (PGD) | Muscular strength (1-RM in leg press, leg curl, lat pull-down, incline press and seated dumbbell) curl and body composition | ↑Muscular strength (EG1 and EG2)  Not differences were reported for the interaction time·intervention |
| Farjallah (2018) ^S71^ | 15 Handball national team (20.8±1.7years) | Not reported | Not reported | EC1: Melatonin  EC2: PLA | 30 min pre-exercise: EC1: 6 mg of melatonin  EC2: 6 mg of PLA (cellulose) | Acute (COD) | Pre- and post-exercise (HIIT):  Oral temperature, vigilance test, glucose, BLA, CMJ, modified agility t-test, maximum standing ball-throw velocity test, 20-m sprint and RPE | Comparison EC1 vs EC2: ↓Glucose and BLA |

BLA: blood lactate concentration; CK: creatin kinase; CMJ: counter movement jump; CrPic: chromium Picolinate; COD: crossover design; CRP: C‑reactive protein;; DJ: drop jump; HIIT: High-intensity interval training; HR: heart rate; HRV: heart rate variability; LDH: lactate dehydrogenase; MC: menstrual cycle; MDA: malondialdehyde; MIN: minutes; PGD: parallel group design; PLA: placebo; RM: repetition maximum; RPE: rate of perceived exertion; SBP: systolic blood pressure; SDP: diastolic blood pressure; TAC: total antioxidant capacity; TBARS: thiobarbituric acid reactive substances; TT: time trial tests; TTE: time-to-exhaustion test; UIBC: unsatured iron-binding capacity; VO2: volume of oxygen; VO2max: maximum oxygen volume consumption.

S60. Cieślicka M, Ostapiuk-Karolczuk J, Buttar HS, Dziewiecka H, Kasperska A, Skarpańska-Stejnborn A, Cieślicka M, Dziewiecka H, et al. Effects of Long-Term Supplementation of Bovine Colostrum on the Immune System in Young Female Basketball Players. Randomized Trial. Nutrients. 2020;13(1):118. Published 2020 Dec 30. doi:10.3390/nu13010118

S61. Skarpańska-Stejnborn A. Effects of Long-Term Supplementation of Bovine Colostrum on Iron Homeostasis, Oxidative Stress, and Inflammation in Female Athletes: A Placebo-Controlled Clinical Trial. Nutrients. 2022;15(1):186. Published 2022 Dec 30. doi:10.3390/nu15010186

S62. Brinkworth GD, Buckley JD, Bourdon PC, Gulbin JP, David A. Oral bovine colostrum supplementation enhances buffer capacity but not rowing performance in elite female rowers. Int J Sport Nutr Exerc Metab. 2002;12(3):349-365. doi:10.1123/ijsnem.12.3.349

S63. Brinkworth GD, Buckley JD. Bovine colostrum supplementation does not affect plasma buffer capacity or haemoglobin content in elite female rowers. Eur J Appl Physiol. 2004;91(2-3):353-356. doi:10.1007/s00421-003-1023-z

S64. Toohey JC, Townsend JR, Johnson SB, et al. Effects of Probiotic (Bacillus subtilis) Supplementation During Offseason Resistance Training in Female Division I Athletes. J Strength Cond Res. 2020;34(11):3173-3181. doi:10.1519/JSC.0000000000002675

S65. McKinley-Barnard SK, Andre TL, Gann JJ, Hwang PS, Willoughby DS. Effectiveness of Fish Oil Supplementation in Attenuating Exercise-Induced Muscle Damage in Women During Midfollicular and Midluteal Menstrual Phases. J Strength Cond Res. 2018;32(6):1601-1612. doi:10.1519/JSC.0000000000002247

S66. Sousa FH, Valenti VE, Pereira LC, et al. Avocado (Persea americana) pulp improves cardiovascular and autonomic recovery following submaximal running: a crossover, randomized, double-blind and placebo-controlled trial. Sci Rep. 2020;10(1):10703. Published 2020 Jul 1. doi:10.1038/s41598-020-67577-3

S67. Bellafiore M, Pintaudi AM, Thomas E, et al. Redox and autonomic responses to acute exercise-post recovery following Opuntia ficus-indica juice intake in physically active women. J Int Soc Sports Nutr. 2021;18(1):43. Published 2021 Jun 7. doi:10.1186/s12970-021-00444-2

S68. Salehi M, Mashhadi NS, Esfahani PS, Feizi A, Hadi A, Askari G. The Effects of Curcumin Supplementation on Muscle Damage, Oxidative Stress, and Inflammatory Markers in Healthy Females with Moderate Physical Activity: A Randomized, Double-Blind, Placebo-Controlled Clinical Trial. Int J Prev Med. 2021;12:94. Published 2021 Jul 29. doi:10.4103/ijpvm.IJPVM_138_20

S69. Brown MA, Stevenson EJ, Howatson G. Montmorency tart cherry (Prunus cerasus L.) supplementation accelerates recovery from exercise-induced muscle damage in females. Eur J Sport Sci. 2019;19(1):95-102. doi:10.1080/17461391.2018.1502360

S70. Livolsi JM, Adams GM, Laguna PL. The effect of chromium picolinate on muscular strength and body composition in women athletes. J Strength Cond Res. 2001;15(2):161-166.

S71. Farjallah MA, Hammouda O, Zouch M, et al. Effect of melatonin ingestion on physical performance, metabolic responses, and recovery after an intermittent training session. Physiol Int. 2018;105(4):358-370. doi:10.1556/2060.105.2018.4.24
