# Supplementary material for: Nutritional Strategies for Optimizing Health, Sports Performance, and Recovery for Female Athletes and Other Physically Active Women: A Systematic Review
Source: Nutr Rev. 2024 Jul 12;83(3):e1068–89. doi: 10.1093/nutrit/nuae082 (PMC11819490; doi:10.1093/nutrit/nuae082)
Supplement: nuae082_Supplementary_Data [file nuae082_supplementary_data.zip › nuae082_Supplementary_Data/ReviewersComments.docx]

**Editor**

Dear editor,

First of all, in representation of the authors of this study, I want to excuse us for the delay in my response. As I wrote you, the main reason for this delayed response were family problems. Therefore, thank you very much for your consideration.

I attached a new version of our manuscript and a rebuttal to the reviewers comments. We have revised each one of the reviewers suggestions. We believe that this new version has increased the quality of the manuscript.

Thank you very much,

Ángel Gil-Izquierdo

**Reviewer: 1**

Thank you for taking the time to review our article. We appreciate very much your feedback and constructive revision. We have revised each one of your suggestions. Your insights are valuable to us, and we are committed to addressing this issue to enhance the overall quality and coherence of the manuscript.

Congratulations on the article, it is very well written but I think it is necessary to fix some points:

TITLE - The title talks about recovery, but in the objective is only health performance; even in the conclusion nothing is said about recovery, the authors should define whether they are going to continue with recovery or not;

Regarding the title discrepancy you noted, we understand your concern. While the title emphasizes recovery, we acknowledge that the objectives primarily focus on health and performance. We recognize the importance of clarity and consistency in our messaging. Therefore, we have revisited the title to ensure alignment with the objectives and conclusions of the article.

B- METHODOLOGY - It is important to define the nutritional strategies according to the phases of the menstrual cycle since the introduction talks about the cycle, but there is no search strategy according to the phases of the cycle;

We agree with you. As it could be checked in the results and discussion sections, it was considered the different phases of the menstrual cycle. This information has been included in the methodology section. Therefore, we have added the next information in the section of data extraction: information about possible control of the menstrual phase (follicular and luteal phases as possible subdivisions in the early, mid or late phase in both, follicular and luteal phases) for the trials or assessments sessions.

Regarding to the search strategy, if we include terms relative to the menstrual cycle, the total results is reduced. This reduction is caused because in the 100% of the studies that have assessed and/or controlled the menstrual cycle phases presents the terms “women” or “females” (the terms of the concept 1 of our search strategy), too.

C-I think that a division should be made in the search strategy according to the type of physical activity, for example: in an Interval training, are the nutritional responses in women the same as in an endurance training?

After careful consideration and consultation with our research team, we have decided not to proceed with this specific division in our search strategy. While we acknowledge the merit of your suggestion and recognize the potential value in investigating differences in nutritional responses across various exercise modalities, we have chosen to maintain a broader approach in our search methodology for several reasons:

- Scope of the Study: Our study aims to provide a comprehensive review of nutritional responses in women across different types of physical activity. By adopting a more inclusive search strategy, we aim to capture a wide range of relevant studies that may contribute to a holistic understanding of the topic.
- Limitations of Available Data: We have assessed the availability and scope of existing literature on this topic and determined that the current body of evidence may not sufficiently support a division based solely on the type of physical activity. While there may be studies specifically addressing nutritional responses in interval training versus endurance training, the overall volume and quality of literature in this area may be limited, potentially compromising the comprehensiveness of our review.
- Practical Considerations: Given the constraints of time and resources, implementing a detailed division in the search strategy based on specific exercise modalities may significantly extend the duration of the literature review process without necessarily yielding substantial additional insights.

D- The description of the results and the discussion would be important to divide them into: Macronutrients, Micronutrients, and supplements. And thus, presented and discussed according to the objectives: Health and Performance.

Thank you for your valuable feedback regarding the organization of the results and discussion sections in our manuscript. We appreciate your suggestion to divide them into categories such as macronutrients, micronutrients, and supplements, according to the objectives of the study.

After a carefully consideration and thorough discussion among our research team, we have opted for an alternative approach to improve the clarity and coherence of the results and discussion sections. We have divided these sections into the following categories:

1. Summarized results of the studies included in the systematic review based on the manipulation of the diet.
2. Summarized results of the studies included in the systematic review based on the manipulation of dietary supplements.

We believe that this division better aligns with the objectives of our study and provides a more focused and streamlined presentation of the findings. By organizing the results and discussion according to the manipulation of the diet and dietary supplements, we can effectively address the different aspects of nutrition relevant to both health and performance outcomes.

This approach allows us to explore the impact of dietary interventions on both macronutrient and micronutrient intake, as well as the use of supplements, while maintaining a clear and structured presentation of the results.

We are confident that this revised organization will enhance the readability and utility of our manuscript, facilitating a more comprehensive understanding of the research findings.

Once again, thank you for your constructive feedback. We will make the necessary revisions accordingly.

**Reviewer: 2**

Dear authors,

here are my comments and suggestions for your manuscript.

Thank you for taking the time to review our article. We appreciate very much your constructive feedback. We have taken each one of your suggestions into consideration.

Your insights are valuable to us, and we are committed to addressing this issue to enhance the overall quality and coherence of the manuscript.

GENERAL

The review is comprehensive in addressing both diet manipulation and supplement effects, providing a holistic view of nutritional strategies for female athletes. Identification of bias risks adds transparency to the assessment of the included studies' quality. Conclusions are clear and supported by compiled findings, emphasizing the importance of carbohydrate-rich diets and the effectiveness of specific supplements.

Thank you very much for the commentaries

INTRODUCTION

The introduction skilfully addresses the performance disparities between male and female athletes, covering not only hormonal differences but also disparities in various physiological aspects. The acknowledgment of the menstrual cycle's influence on hormonal variations and its potential impact on metabolism provides a nuanced approach to understanding the unique needs of women athletes. By incorporating these recommendations, the introduction can further strengthen its foundation, ensuring that readers grasp the significance of the systematic review and its potential contributions to the field of women's sports nutrition.

Thank you very much for the commentaries

Recommendations for the Introduction:

• While the introduction mentions the aim of the study, a more explicit statement regarding the specific objectives and contributions of the systematic review would enhance clarity for readers.

In response to your suggestion, we have revised the objectives of the study to include "recovery" as a key aspect. By incorporating "recovery" into the stated objectives, we aim to provide a comprehensive overview that encompasses all relevant aspects considered in the review.

• To strengthen the relevance of the study, consider incorporating any recent research findings or developments in the field of women's sports nutrition (during these months). This could provide additional context.

After thorough consideration and review of the current literature, we regret to inform you that there have been no significant recent research findings or developments in the field that would warrant inclusion in the introduction of our systematic review.

While we continuously monitor the latest research in women's sports nutrition, we have found that the existing body of literature remains relatively stable within the timeframe of our review. However, there is a new important review that analyse differences based on sex in physical performance, and we have included it in the introduction section:

Nuzzo JL. Narrative review of sex differences in muscle strength, endurance, activation, size, fiber type, and strength training participation rates, preferences, motivations, injuries, and neuromuscular adaptations. *J Strength Cond Res.* 2023;37(2):494–536

• Consider reorganizing or breaking down lengthy sentences for better readability and comprehension. This will enhance the flow of information and aid in capturing the reader's attention.

We have carefully considered your comment and have taken steps to address it in our revision.

METHODOLOGY

The methodology section appropriately states adherence to the Preferred Reporting Items for Systematic Reviews and Meta-Analyses (PRISMA) guidelines, ensuring transparency and methodological rigor. Furthermore, the registration of the current protocol in the International Prospective Register of Systematic Reviews (PROSPERO) adds credibility to the study. Moreover, the inclusion of a detailed description of the literature search strategy, including databases, time frame, and language restrictions, enhances transparency and replicability. The use of PICOS criteria for establishing eligibility criteria is well-structured and aligns with best practices in systematic reviews, providing clarity on population, intervention, comparison, outcomes, and study design. Additionally, the use of Covidence systematic review software for screening and decision recording is a sound approach. The involvement of multiple reviewers in the screening and review phases, along with a third reviewer for conflict resolution, strengthens the reliability of the study selection process. The use of Covidence for data extraction and the clear outline of the information collected from each study enhances the systematic and standardized approach to data synthesis. The adoption of the modified Cochrane risk-of-bias tool (RoB 2) for assessing the quality of included studies is appropriate. The categorization of risks as "low," "unclear," or "high" adds transparency to the evaluation process.

To improve the article, I suggest different recommendations. Addressing these recommendations will contribute to the overall clarity, transparency, and rigor of the methodology, ensuring a robust foundation for the systematic review.

Thank you very much for the commentaries.

Recommendations for the Methodology:

• For the eligibility criteria related to the population, consider providing a clear and standardized definition of what constitutes "physically active" to avoid potential ambiguity in study inclusion.

In response to your comment, we want to assure you that we have taken into account the recommendations of the American College of Sports Medicine (ACSM) for defining physically active individuals. We have carefully reviewed and incorporated these guidelines to establish a clear and standardized definition of what constitutes "physically active" for the purpose of our study.

By adhering to recognized definitions and guidelines, we aim to minimize ambiguity and ensure consistency in the identification and inclusion of physically active individuals across the studies included in our review.

• While it is mentioned that studies published in English, Portuguese, and Spanish were included, providing a brief rationale for this language selection would enhance the transparency of the methodology.

We want to assure you that the decision to include studies published in English, Portuguese, and Spanish was made intentionally to ensure inclusivity and comprehensiveness in our literature search. These three languages were selected based on the prevalence of scientific publications in the field of [your field of study] and to minimize the risk of excluding relevant studies due to language barriers.

English, Portuguese, and Spanish are widely recognized as the primary languages of scientific communication, and including studies published in these languages enables us to capture a diverse range of research findings and perspectives from different geographical regions.

• Incorporate a brief rationale for selecting the time frame (January 2000 to July 3, 2023) to justify the inclusion or exclusion of studies within this period.

The decision to establish the time frame from January 2000 to July 3, 2023, was based on several factors aimed at ensuring the comprehensiveness and relevance of our literature search. By selecting this time frame, we aimed to capture all relevant studies conducted since the beginning of the century up to the most recent available data.

This time frame allows us to encompass a significant period of research and to provide a comprehensive overview of the literature. Additionally, it ensures that we include the latest developments and findings in the field while still covering a sufficiently broad range of studies to provide meaningful insights.

• While Table 1 mentions the PICOS criteria, consider providing a brief narrative or explanation of how the table was used in the study selection process.

Upon reviewing your comment and revisiting the manuscript, we believe there may be a misunderstanding. The purpose of Table 1 is to provide a summary of the PICOS criteria used in our study selection process. Each row of the table corresponds to one aspect of the PICOS criteria: Population, Intervention, Comparison, Outcome, and Study design. We have ensured that the PICOS criteria are clearly described in the manuscript, and Table 1 serves as a visual representation of how these criteria were applied during the study selection process.

While we acknowledge the importance of providing a narrative or explanation of how the table was used in the study selection process, we believe that the information presented in Table 1 adequately fulfils this purpose. However, if there are specific aspects of the table or the study selection process that require further clarification, we would be more than happy to address them.

• Mention the version of the Covidence tool used for screening and recording decisions, as tool versions may be updated over time.

In response to your comment, we have updated the manuscript to include the website of the software used for screening and recording decisions.

RESULTS

This section presents a clear presentation of the selection process, a transparent reporting of results, and detailed characteristics of included studies. Moreover, it has a comprehensive overview of dietary interventions and a thorough overview of dietary supplements.

The inclusion of a risk-of-bias assessment enhances the transparency of the systematic review. The identification of potential biases, such as the lack of reporting and protocols, provides valuable insights into the quality of the included studies.

However, below are some recommendations to improve this section.

Thank you very much for the commentaries.

Recommendations for the Results Section:

• Provide a brief explanation of the criteria used for removing three articles based on statistical analysis. This clarification would enhance transparency regarding the decision-making process.

The decision to exclude these three articles was based on specific criteria related to statistical analysis errors identified during the screening process. As outlined in the PRISMA flow diagram (Figure 1), these errors rendered the statistical analyses of these studies invalid and compromised their reliability and validity.

• If applicable, include definitions or explanations of abbreviations and terms used in the results section to facilitate understanding for readers who may not be familiar with specific terms.

Throughout the manuscript, we have made efforts to explain and clarify any abbreviations and terms used, particularly those that may not be commonly known. These explanations have been provided within the text as the abbreviations are introduced, aiming to enhance readability and comprehension for all readers.

However, we acknowledge the importance of ensuring clarity and accessibility for all readers. If the editor deems it necessary, we have no objection to including a separate section with the abbreviations used throughout the article along with their definitions or explanations. This additional section could serve as a quick reference guide for readers seeking clarification on specific terms.

• Briefly discuss any limitations of the included studies or potential sources of bias, acknowledging the challenges and constraints that may affect the overall interpretation of the results.

We want to assure you that the aspect you mentioned, regarding the limitations of the included studies and potential sources of bias, has been addressed in the conclusions section of our manuscript. In this section, we have provided a brief discussion of the challenges and constraints that may affect the overall interpretation of the results, including any limitations inherent in the studies included in our systematic review.

However, we understand the importance of ensuring that this discussion is prominently featured and easily accessible to readers. If you believe it would be more appropriate to include a separate section specifically dedicated to discussing the limitations and potential sources of bias, we are open to making this adjustment. Nevertheless, we believe that concluding the article in this manner provides a comprehensive summary that highlights the significance of our findings and underscores the need for further research in this area.

• For studies with specific sample characteristics, such as eumenorrheic participants or those in menopause, consider providing additional context or implications for the interpretation of results.

Throughout the text, we have strived to provide context and implications for each situation or article, including those involving participants with specific sample characteristics. Examples of this can be found on pages 12, 13, 20, and 30, where we have elaborated on the implications of the findings in relation to the characteristics of the study participants.

However, we understand the importance of ensuring that this information is clearly conveyed and readily accessible to readers. If you believe that further clarification or elaboration is necessary for specific situations, we are more than willing to provide additional context as requested. Nevertheless, we believe that the implications for each situation have been sufficiently explained throughout the text.

DISCUSSION

The discussion section offers valuable insights into sport nutrition on women athletes. However, below are some recommendations to improve this section. Incorporating these recommendations into the discussion section will strengthen the overall research narrative and contribute to the advancement of knowledge in the field of nutrition for women athletes. these recommendations are meant to further refine an already comprehensive and informative discussion section.

Recommendations for the Discussion Section:

• Emphasize the importance of increasing research efforts specifically focused on women athletes to address the current gender disparity in literature.

We want to assure you that the aspect you mentioned has been addressed in the conclusions section of our manuscript to emphasize this importance.

• Encourage future studies to consider the menstrual cycle in trial designs to enhance the validity and relevance of dietary recommendations for female athletes.

We want to assure you that the consideration of the menstrual cycle in trial designs, as well as its implications for the interpretation of results, is addressed in several sections of our manuscript. Specifically, the Risk-of-bias assessment section provides insights into the methodological quality of the included studies, including any considerations related to the menstrual cycle. Additionally, the Conclusion section highlights the key findings and implications of our systematic review, which may include recommendations for future research regarding trial designs that account for the menstrual cycle.

Furthermore, as you mentioned, the contextualization of this topic can also be found in the Discussion section, as exemplified by the specific reference you provided ("Only one study considers the phase of the menstrual cycle in terms of the effect of CR supplementation in women" on page 40). This discussion underscores the importance of considering the menstrual cycle in research designs and offers recommendations for future studies.

• Highlight the need for further research on the nuanced effects of carbohydrate manipulation strategies, considering exercise intensity and duration.

Throughout the manuscript, both exercise intensity and duration have been carefully considered and discussed in various contexts. Numerous instances can be found where either or both of these factors are cited and analyzed in relation to carbohydrate manipulation strategies (for example). These references are scattered throughout the text and are particularly prominent in sections discussing study methodologies, results interpretation, and implications for practice.

In the Discussion section, we have also addressed the implications of exercise intensity and duration on the effectiveness of carbohydrate manipulation strategies, highlighting the need for future research to delve deeper into these nuances. For example, specific discussions on the potential impact of exercise intensity and duration can be found in the paragraph discussing the limitations of the current body of evidence and suggestions for future research directions.

However, we acknowledge the importance of explicitly highlighting this aspect to underscore its significance. To address this, we could consider emphasizing the discussion points related to exercise intensity and duration in the Conclusion section, providing a succinct summary of the need for further research in this area.

If you believe there are specific sections where this aspect could be further emphasized or if there are suggestions on how we can improve the manuscript in relation to this comment, we welcome your guidance. Your insights are invaluable in ensuring the quality and relevance of our work.

• Encourage additional studies assessing the impact of calcium intake, especially from dairy sources, on bone health, exercise recovery, and overall performance in female athletes.

We would like to direct your attention to page 23 of our manuscript, where an extended discussion on the impact of calcium intake is provided. In this section, we delve into the potential effects of calcium intake on bone health, exercise recovery, and overall performance in female athletes. We believe that this comprehensive discussion offers valuable insights into the role of calcium in supporting athletic performance and recovery, particularly in female athletes.

Furthermore, we fully endorse your encouragement for additional studies to further investigate the impact of calcium intake, particularly from dairy sources, on various aspects of female athletes' health and performance. The importance of this research cannot be overstated, as it has significant implications for optimizing the nutritional strategies of female athletes to support their overall well-being and athletic endeavors.

• Recommend further investigations into optimal protein intake distribution strategies to maximize the anabolic effects of protein on muscle synthesis and functional changes.

• Emphasize the importance of considering and controlling for confounding factors in studies, such as dietary intake variations, hormonal fluctuations, and use of contraceptives.

We have carefully reviewed your comment and have added additional text in the discussion section to address this topic more comprehensively. We believe that this addition significantly enhances the quality and relevance of our discussion by highlighting the need to account for these confounding factors in studies related to our research.

• Clearly articulate the practical implications of the findings for women athletes, coaches, and practitioners. How can this information guide dietary and supplementation practices in real-world athletic settings?

The aspect you mentioned has been addressed in the conclusions section of our manuscript to emphasize this importance.

• Expand on suggestions for future research directions. Identify gaps in the current literature and propose areas where further investigation is needed to advance the understanding of dietary supplementation for women athletes.

The aspect you mentioned has been addressed in the conclusions section of our manuscript to emphasize this importance.

• Emphasize the importance of conducting more gender-specific research to address the existing gaps in understanding how these supplements affect women differently from men.

We have added additional text in the conclusion section to address this topic more comprehensively.

• Highlight the potential variation in responses to supplements among women due to factors such as age, fitness level, and individual differences.

We have added additional text in the discussion section to address this topic more comprehensively.

• Provide clear recommendations for future research directions, including the need for larger sample sizes, well-controlled studies, and investigations into the combined effects of multiple supplements.

We have added additional text in the discussion and conclusion sections to address this topic more comprehensively.

• Address safety concerns and potential side effects associated with supplement use, emphasizing the importance of considering the overall well-being of athletes.

While addressing safety concerns and potential side effects associated with supplement use is important, we believe that emphasizing this aspect may detract from the primary focus of our review, which is to explore dietary strategies, including dietary and supplementation approaches, that can enhance performance, recovery, and/or health status in women athletes. However, we acknowledge the significance of considering the overall well-being of athletes and ensure to discuss safety concerns within the scope of our review.

• Emphasize the importance of including menstrual and menopausal athletes in research studies to better understand their unique nutritional needs.

We have added additional text in the discussion and conclusion sections to address this topic more comprehensively.

• Recommend more longitudinal studies to assess the long-term impact of dietary strategies on the performance and health of women athletes.

We have added additional text in the conclusion section to address this topic more comprehensively. We believe that this addition significantly enhances the quality and relevance of our discussion by highlighting the need to account for these confounding factors in studies related to our research.

• Promote educational initiatives to raise awareness among women athletes about the importance of nutritional intake and its impact on performance and health.

We have added additional text in the conclusion section to address this topic more comprehensively. We believe that this addition significantly enhances the quality and relevance of our discussion by highlighting the need to account for these confounding factors in studies related to our research.

• Further emphasize the scarcity of studies involving women in the context of carbohydrate (CHO) effects and dietary supplement assessments. This would reinforce the necessity of the systematic review.

The aspect you mentioned has been addressed in the conclusions section of our manuscript to emphasize this importance.

CONCLUSIONS

The conclusions section effectively summarizes the limitations in current research, emphasizes the importance of increased focus on women athletes, and provides valuable recommendations for future studies and practical applications. Overall, the article makes a valuable contribution to the field of female sports nutrition, offering relevant information for athletes and healthcare professionals. However, below is one recommendation to improve this section.

• Addressing identified limitations, such as the lack of access to study protocols and improving the description of hormonal control during the menstrual cycle, is suggested to enhance the internal validity of future studies.

We have added additional text in the conclusion section to address this topic more comprehensively.
